# Agglomerative Clustering of Enteric Infections and Weather Parameters to Identify Seasonal Outbreaks in Cold Climates

**DOI:** 10.3390/ijerph16122083

**Published:** 2019-06-12

**Authors:** Pavel S. Stashevsky, Irina N. Yakovina, Tania M. Alarcon Falconi, Elena N. Naumova

**Affiliations:** 1 Novosibirsk State Technical University, Novosibirsk 630087, Russia; stashpavel@gmail.com (P.S.S.); irina.nir@gmail.com (I.N.Y.); 2 Friedman School of Nutrition Science and Policy, Tufts University, Boston, MA 02111, USA; tania.alarcon_falconi@tufts.edu; 3 Department of Civil and Environmental Engineering, Tufts University School of Engineering, Medford, MA 02155, USA

**Keywords:** machine learning, agglomerative clustering, *t*-SNE method, harmonic regression models, salmonellosis, non-specific enteric infections, seasonality, meteorological parameters, climate change

## Abstract

The utility of agglomerative clustering methods for understanding dynamic systems that do not have a well-defined periodic structure has not yet been explored. We propose using this approach to examine the association between disease and weather parameters, to compliment the traditional harmonic regression models, and to determine specific meteorological conditions favoring high disease incidence. We utilized daily records on reported salmonellosis and non-specific enteritis, and four meteorological parameters (ambient temperature, dew point, humidity, and barometric pressure) in Barnaul, Russia in 2004–2011, maintained by the CliWaDIn database. The data structure was examined using the *t*-distributed stochastic neighbor embedding (*t*-SNE) method. The optimal number of clusters was selected based on Ward distance using the silhouette metric. The selected clusters were assessed with respect to their density and homogeneity. We detected that a well-defined cluster with high counts of salmonellosis occurred during warm summer days and unseasonably warm days in spring. We also detected a cluster with high counts of non-specific enteritis that occurred during unusually “very warm” winter days. The main advantage offered by the proposed technique is its ability to create a composite of meteorological conditions—a rule of thumb—to detect days favoring infectious outbreaks for a given location. These findings have major implications for understanding potential health impacts of climate change.

## 1. Introduction

In health-related fields, including life sciences and epidemiology, the relationship between weather patterns and diseases represents a well-documented example of temporal interactions with highly anticipated predictive capacity [1,2]. Enteric infections caused by *Salmonella*, *Cryptosporidium*, *Giardia*, and many other pathogens are well known for their strong associations with ambient temperature and precipitation [3,4,5,6]. These infections are notorious for seasonal outbreaks which are difficult to track and control. The link between meteorological factors and water- and foodborne infections has been an important subject of biomedical research aiming to identify proxies to predict potential outbreaks and ultimately to develop strategies that minimize risks [7,8]. In temperate climates, bacterial enteric infections such as salmonellosis have been shown to peak in summer months due to an increased probability of exposure and transmission [6,9]. However, the temporal patterns of unspecified infections (i.e., infections for which the etiological cause is not determined) could vary substantially, making disease forecasting difficult. As we demonstrated in our previous work [3], the seasonal increases in unspecified infections matched to the peaks of well-established infections can provide insights into the etiology, risk factors, and transmission patterns of unspecified infections, and help recommend strategies to fine tune disease diagnostics and forecasting. 

Harmonic regression models (HRMs) adapted to time-referenced data are commonly used to describe temporal patterns in epidemiology of infectious diseases, focusing on defining trends, seasonal peaks, effects of social events, and other cyclic behaviors. This class of models has also been applied to determine the associations between the incidence of infection and weather parameters, assuming a specific form of relationship is known or at least correctly specified [6,10,11]. HRMs show the best performance in explaining the relationship between health outcomes and weather parameters when the correlation structure among predictors is weak, which is rare in temperate climates. HRMs assume a well-defined periodic structure of the data, which may not be warranted for some diseases and weather parameters. With a continuous change in climatic conditions and extreme weather profiles, rapid and unusual aperiodic swings in the weather have been observed worldwide [12,13]. Climate change affects local weather, influences the dynamics of infectious pathogen transmission, and ultimately triggers changes in infectious diseases morbidity and mortality. In these dynamic situations, the descriptive, explanatory and predictive capacities of traditional HRMs are limited and alternative solutions need to be explored. 

Methods involving clustering of time-series data are expanding in many scientific fields due to a potential versatility in creating a complete set of nested cluster solutions [14,15,16,17]. These methods aim to extract valuable information from complex datasets and to discover temporal patterns, adding new tools to an array of classic statistical approaches operating on time or frequency domains. Hierarchical clustering is gaining popularity in many fields of life sciences as an effective technique for identifying distinct homogeneous clusters [14,15]. These methods may reduce the burden of initial assumptions on the underlying data structure. Hierarchical clustering involves creating clusters that have a predetermined ordering from top to bottom, which makes them suitable for building taxonomies. In agglomerative clustering, each observation is assigned to its own cluster, and then the two most similar clusters are merged based on a similarity metric (e.g., distance) between each of the clusters. The process is repeated until there is only a single cluster left. Each of the clusters can be further explored to characterize their typical features, to build a classification system, and to discover anomalies. Clustering of records in a single time series illustrates characteristic disease patterns, like seasonal oscillations. Clustering of records in multiple time series (e.g., disease, temperature, and precipitation) may allow to determine groups or variables with similar temporal patterns. If the patterns are synchronized in some specific way, it might be possible to detect the features that have a capacity to predict high or low disease incidence. These features of hierarchical clustering make them suitable for exploring the association between disease and weather parameters, especially when studying disease dynamics affected by climate change. 

In the presented study, we propose a hierarchical agglomerative approach for cluster identification in multiple time series. We demonstrate its potential to identify infectious outbreaks based on environmental predictors, specifically a relevant set of weather conditions. We considered enteric infections transmitted by food and water (salmonellosis and bacterial intestinal infection) and metrological factors, such as atmospheric temperature, barometric pressure, relative humidity, and dew point in Barnaul, Russia, in 2004–2011. The daily records for infections and weather parameters were maintained by the Climate-Water-Diseases-Infections (CliWaDIn 1.0) database [8,18]. The preliminary analysis and visualization of the data structure was performed using the t-distributed stochastic neighbor embedding (*t*-SNE) method. The optimal number of clusters was selected based on Ward distance using the silhouette metric. The selected clusters were further assessed with respect to their density and homogeneity. We clustered the time series data into groups of days with similar values of meteorological factors and disease counts. By presenting this goal as an unsupervised machine learning task, we identified time periods when meteorological conditions “favor” infections. The main advantage offered by the proposed technique is its ability to create a composite of meteorological conditions—a rule of thumb—to detect days with “unseasonable weather” favoring infectious outbreaks for a given location.

## 2. Data and Methods 

The study utilizes meteorological records of the public weather server “The Weather of Russia” (http://meteo.infospace.ru/) and passive surveillance records curated and maintained by the CliWaDIn version 1.0 database [8]. The database contains records of enteric waterborne infections for six cities in Russia (Novosibirsk, Barnaul, Ekaterinburg, Chelyabinsk, Krasnoyarsk, Vladivostok). In this analysis, we used data from the city of Barnaul from 1 January 2004 to 31 December 2011. Barnaul, the administrative center of Altai Krai, Russia, is located on the left bank of the Ob River at the confluence with the Barnaulka River (Latitude: 53° 21′ 38.02″ N; Longitude: 83° 45′ 49.00″ E), in the forest steppe zone of the West Siberian Plain. As of the 2010 Census, the city of Barnaul had 612,401 residents. 

### 2.1. Health Outcomes

**Figure 1:**
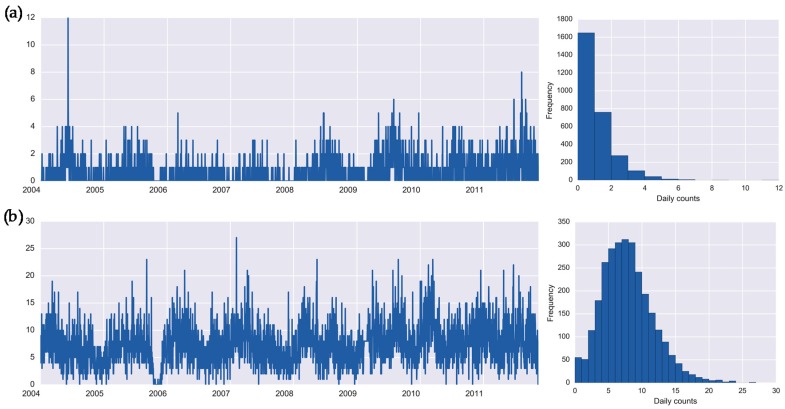
Daily time series and histograms for health outcomes: (**a**) salmonellosis, A02.8, and (**b**) enteritis, A04.9 in Barnaul, Russia in 2004–2011.

Daily counts of infections, recorded as “other specified salmonella infections” and “Bacterial intestinal infection or unspecified bacterial enteritis” and defined by International Classification of Diseases (ICD-10) as A02.8 and A04.9, are referred thereafter as salmonellosis (1901 cases) and enteritis (21,019 cases), respectively. Figure 1 shows the original time series of counts, illustrating the temporal dynamics of infections, and the histograms, reflecting the distributional shape of the temporal process. 

Enteritis has a higher mean incidence rate (7.38 vs. 0.67 cases per day) as compared to salmonellosis. As shown in the histogram (Figure 1a), the first bar indicates a high proportion of days (58%) with no cases of salmonellosis. The plots and descriptive statistics show that salmonellosis has prolonged periods with no cases and periodic outbreaks of infection in summer months, whereas the daily counts of enteritis fluctuate around the mean with loosely defined seasonality and occasional spikes of high counts. The daily counts are presented in Supplemental Table S1.

### 2.2. Meteorological Records

**Figure 2:**
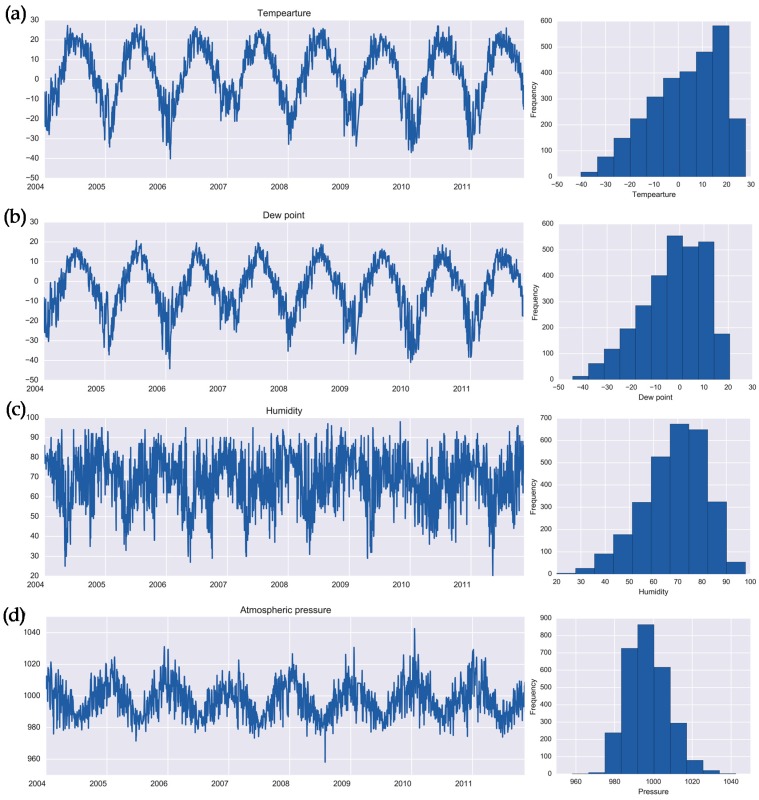
Daily time series and histograms for weather parameters: (**a**) temperature (°C), (**b**) dew point (°C), (**c**) relative humidity (%), and (**d**) atmospheric pressure (hPa) in Barnaul, Russia in 2004–2011.

The study region has a continental relatively dry climate (Köppen Dfb) with a wide range of temperature extremes from below −45 °C (−49°F) in the winter (defined as three consecutive months: December, January and February) to above +35 °C (95 °F) in the summer (defined as three consecutive months: June, July and August). We abstracted the average daily values of temperature (°C), atmospheric pressure (hPa), relative humidity (%) and dew point (°C) for 2848 days of the study period from 1 January 2004 to 15 November 2011 (Supplemental Table S1). The proportion of missing records is low (1% or 28 days out of 2849 days) with 2 weeks of missing data in late February and March of 2009. The time series of weather parameters have pronounced seasonal variations with no clearly marked trend. Table 1 contains summary statistics for health outcomes and meteorological data. The time series of daily averages for weather parameters and their distributions are shown in Figure 2. 

### 2.3. Agglomerative Clustering

The construction of hierarchical clustering can be performed in a divisive or in an agglomerative manner. In divisive clustering, all the observations are assigned to a single cluster, which is then partitioned into two least-similar clusters. The process is repeated recursively on each cluster until there is one cluster for each observation. Agglomerative clustering offers a viable alternative to the k-means algorithm, another popular classification approach. In agglomerative clustering, clusters are created by linking each data point to its nearest neighbor using a distance measure, as opposed to creating a cluster based on centroids as for the k-means algorithm. The number of clusters in agglomerative clustering is determined by reiterating the algorithm with different linkage methods, so all the available points converge into a rapidly diminishing number of clusters until they all comprise a single group. While agglomerative clustering algorithms are more cumbersome and computationally demanding than the k-means methods, they create a complete range of nested cluster solutions. This feature is especially attractive when we need to classify finite series of events that occurred under specific conditions. For example, in epidemiologic investigations we often need to define conditions that are favorable for disease transmission or harmful exposures. 

We denote the daily time series of disease counts, *z_1t_* and *z_2t_*, for salmonellosis and enteritis, respectively, and a set of time series of weather parameters, *w_mt_*, where *m = {1,…,4}* indicate a parameter, *t = {1,…,T}* is a study day, and *T* is the effective length of time series, *T* = 2848 days. As illustrated in Figure 1 and Figure 2, *z_1t_* and *z_2t_* are assumed to be derived from a Poisson distribution with parameters λ_l_ and λ_2_, respectively; and *w_mt_*, are assumed to be derived from a multivariate normal distribution, *N*(**μ**, Σ). 

The task of clustering the time series of disease counts and weather parameters consisted of multiple steps. First, we consider a time series as a sample *X^T^ = {x_1_, …, x_T_}*, where an object *x_i_* is a certain day with the centered daily values of disease counts and meteorological parameters, say *x_i_ = {z_1i_, w_1i_, w_2i_, w_3i_, w_4i_}*. To each day *x_i_∈X^T^*, we assign a label, or a cluster identifier, of class *y_i_*. Next, to describe and visualize the multidimensional data, we examine the clustering structure using the *t*-Distributed Stochastic Neighbor Embedding (*t*-SNE) method [19], which relies on the conditional probabilities, as follow (Equation (1)):

(1) $$p_{j|i}=\frac{exp\;(-{\left|x_{i}-x_{j}\right|}^{2}/2\sigma_{i}^{2})}{\sum_{k\neq i}exp\;(-{\left|x_{i}-x_{k}\right|}^{2}/2\sigma_{i}^{2})}$$

The conditional probability $p_{j|i}$ conveys how close the values of day *x_j_* are to those of day *x_i_*, given a normal distribution centered on *x_i_* with variance *σ_i_^2^* [19]. The variance *σ_i_^2^* of each day was selected considering the *perplexia* estimate, such that the days with the most common values of disease counts and weather parameters, or in other words days with higher density, have a smaller variance [19]. For days with similar disease counts and meteorological parameters, $p_{j|i}$ should be relatively high, whereas for days with very different counts and meteorological parameters, $p_{j|i}$ should be almost infinitesimal. 

To perform the agglomerative clustering, we split the segments of the time series into disjoint clusters starting with *T* clusters of size 1 and continue until all the days are included into one cluster with the size of *T*. In the first step of the algorithm, *T-1* clusters are formed, one of size two and the remaining of size 1. The rule to join two days into one cluster is based on a Ward distance metric, *R* [20] aiming to minimize the *R(C, H)* value. Thus, at each step of the algorithm we combine clusters with the minimum distance value. The algorithm stops when all sample units are combined into a single large cluster of size *T*. At each step, Ward distances between clusters were calculated, as follow (Equation (2)):

(2) $$R\left(C,H\right)=\frac{\left|C\right|\left|H\right|}{\left|C\right|+\left|H\right|}\rho^{2}\left(G^{C},G^{H}\right)$$

where *C* and *H* are clusters; *ρ^2^* is the Euclidian distance between the geometric centers *G^C^* and *G^H^* of the clusters *C* and *H*; and *|C|* and *|H|* are the numbers of objects (or days) in the clusters *C* and *H,* respectively. Ward’s distance was chosen because it has tensile properties and shows good results in preliminary experiments on clustering [20,21,22]. These properties were ensured by applying normalization and standardization to each variable before the clustering procedure. In addition, the standard procedure implemented in the adapted package used PCA to transform data (the forward transformation—before clustering and reverse transformation—after clustering) to minimize the correlation across the features. Next, to assess the quality of clustering and to choose the number of clusters, we maximized the *silhouette metric* [20] (Equation (3)):

(3) $$S=\frac{\rho^{2}{}^{\prime }-\rho^{2}{}^{\prime \prime }}{\max\left(\rho^{2}{}^{\prime },\;\rho^{2}{}^{\prime \prime }\right)}$$

where $\rho^{2}{}^{\prime }$ is the Euclidian distance from a given object to objects within the same cluster, and $\rho^{2}{}^{\prime \prime }$ is the Euclidian distance from a given object to objects from the nearest cluster (different from the own cluster). The silhouette metric is calculated separately for each object *x_i_* of the sample (i.e., each day of the time series). In the context of a daily time series, “distances from one day to another” can be interpreted as similarities among days based on disease counts and the averages of meteorological factors. Thus, the silhouette metric for time series data can be interpreted as a similarity profile of time series segments (clusters). 

To determine the number of clusters, we used computational experiments to calculate the silhouette metric with the number of clusters ranging from 4 to 15. The lower limit of four clusters captures the four calendar seasons characteristic for cold temperate climates. The upper limit of 15 clusters was chosen to warrant at least two major seasons over the study period. For each experiment, we calculated the silhouette metric and chose the optimal number of clusters according to the maximum value of the silhouette metric.

Statistical characteristics (mean, standard deviation, interquartile range, and kurtosis) were calculated for all clusters. We used the values of cluster interquartile range (IR) and kurtosis as metrics of clustering quality, with an emphasis on clustering density and homogeneity. The interquartile range, a robust analogue of the variance, was used to indicate clustering density or diffusion (the higher the IR the more diffuse is the cluster). We used kurtosis as a marker of homogeneity. Thus, the clusters with smaller values of both metrics are preferred.

### 2.4. Log-Linear and Harmonic Regression Models

To ease the interpretation of clustering results we also performed the traditional negative binomial harmonic regression and estimated seasonal peak timing for each investigated health outcome [5,6]. The basic Model A was formulated as follows (Equation (4)):

*ln*(E[*z_t_*]) = *β*_0_ + *β*_s_ sin(2πω*t*) + *β*_c_ cos(2πω*t*) (4)

where z_t_ is the daily counts for *z_1t_* and *z_2t_*, e.g., salmonellosis and enteritis, respectively; seasonality in disease cases was assessed based on the significance of the two harmonic terms with t as consecutive days of the study period, and *ω* = 1/365.25. Estimates obtained from fitting Model A can be used to compute phase shift ϕ based on join signs of ${\hat{\beta }}_{s}\;\mathrm{and}\;{\hat{\beta }}_{c}$:

(5) $$\hat{\phi }=arctan\left(\frac{{\hat{\beta }}_{s}}{{\hat{\beta }}_{c}}\right)+k$$

where k=0 when both ${\hat{\beta }}_{s}\;\mathrm{and}\;{\hat{\beta }}_{c}$ are positive, k=2π when ${\hat{\beta }}_{s}<0\;\mathrm{and}\;{\hat{\beta }}_{c}>0$, and k=π otherwise. Peak timing is calculated by multiplying the phase shift by 365.25/2π. Using the δ-method [6], variance (Var) and standard deviation σ of the $\hat{\phi }$ estimates can be estimated as:

(6) $$Var\left(\hat{\phi }\right)=\frac{{\left({\hat{\sigma }}_{\beta_{s}}{\hat{\beta }}_{c}\right)}^{2}+{\left({\hat{\sigma }}_{\beta_{c}}{\hat{\beta }}_{s}\right)}^{2}-\left(2{\hat{\sigma }}_{\beta_{s}\beta_{c}}{\hat{\beta }}_{s}{\hat{\beta }}_{c}\right)}{{\left({\hat{\beta }}_{s}^{2}+{\hat{\beta }}_{c}^{2}\right)}^{2}},\;\sigma_{\hat{\phi }}=\sqrt{Var\left(\hat{\phi }\right)}$$

where $\sigma_{\beta_{s}}^{2}$ and $\sigma_{\beta_{c}}^{2}$ are the variances of the estimates of $\beta_{s}$ and $\beta_{c}$, respectively, and $\sigma_{\beta_{s}\beta_{c}}$ is the covariance. 

We then explored the sensitivity of the peak timing estimates and repeated the modeling by sequentially adding the first order autocorrelation term (Model A1) and indicator variables for day of the week (Model A2).

We examined the potential form of associations among daily values of health outcomes and all predictors using loess smoother with the span parameter of 0.75 and estimated pair-wise Spearman correlation coefficients. We then fitted a standard generalized negative binomial regression to quantify the association between daily counts of the health outcome, *z_t_*, and weather parameters, *w_jt_* (Model B):

*ln*(E[*z_t_*]) = *β*_0_ + *β_m_ w_mt_* (7)

where *β_m_* is the regression coefficient for the *m*-weather parameter. Since ambient temperature, pressure, and dew point values are highly correlated, we ran Model B with each weather parameter separately. Relative risks (RR) associated with a 10 unit of change in each weather parameter and their 95th confidence intervals (CI_95%_) are estimated as *RR_j_* = *exp*{10 *β_m_*} and *CI_95%_ = exp*{10(*β_m_* ± 1.96*σ_β_m__*)}, respectively. The Model B was then further explored with respect to its stability by adding sequentially the first order autocorrelation term, seasonal harmonics, and indicator variables for day of the week. 

The following software was used in the experiments: programming language Python 2.7 and Scikit-learn framework version 0.18 (http://scikit-learn.org/) to implement *t*-SNE and hierarchical clustering methods; R version 3.5.1 (R Core Team, Vienna, Austria) and R-Studio version 1.1.463 (RStudio, Boston, MA, USA) to run harmonic and log-linear modeling. 

## 3. Results

**Figure 3:**
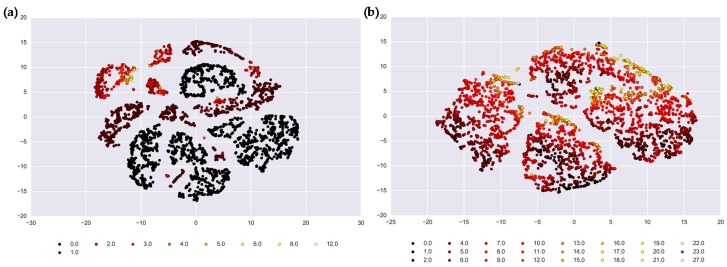
Results of *t*-SNE analysis depicted by the clustering distances in the vertical and horizontal axes for: (**a**) salmonellosis, A02.8, and (**b**) enteritis, A04.9 in Barnaul, Russia in 2004–2011. Colors represent health outcome counts from 0 to 12 for salmonellosis and 0 to 27 for enteritis.

Results of the *t*-SNE method showed the preliminary cluster structure (Figure 3) in a multidimensional space projected on two dimensionless axes. These axes reflect the clustering distances, or normalized composites of all records from the multiple time series, forming a distance matrix that allows us to identify individual days that form a similar pattern based on the weather parameters and health outcome values. Colors represent health outcome counts from 0 to 12 for salmonellosis and 0 to 27 for enteritis. We considered the results of *t*-SNE visualization as a supporting tool for preliminary assessment of clustering and of the number of clusters in the original time series. Visually, the time series of salmonellosis counts may contain from 8 to 12 clusters. The clusters of days with nonzero counts of salmonellosis are concentrated in the upper left part of the panel; and clusters of days with no cases (shown in black) can be clearly distinguished. For enteritis, there appear to be four major clusters, which are likely to refer to four climatic seasons, governed by the weather characteristics, but not by disease outcomes. Days with higher counts (shown in light orange and yellow) are concentrated in distinct areas in each of those four clusters. Yet, the clustering is not as apparent and requires further partitioning and quantification, as presented in the next section.

**Figure 4:**
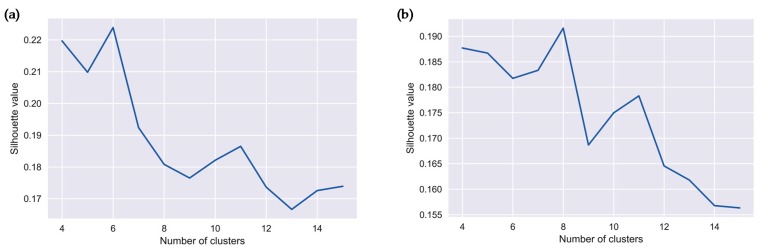
Silhouette metric values and number of clusters for: (**a**) salmonellosis, A02.8, and (**b**) enteritis, A04.9 in Barnaul, Russia in 2004–2011.

To determine the optimal number of clusters describing the original data, we used the silhouette metrics calculated for each sample. The silhouette metric values for agglomerate clustering with the Ward distance are shown in Figure 4. The maximum value of the metric for salmonellosis was 0.224 and indicated the presence of six clusters, and for enteritis it was 0.192 and indicated the presence of eight clusters. In addition, a local maximum of the silhouette value indicated the presence of eleven clusters for both infections. These results justify additional analysis to characterize the clusters corresponding to the global and local maximum values of the silhouette metric associated with two sets of clusters. 

The results of further analysis described below focus on salmonellosis and enteritis infections by presenting the clustering as a sequence of two sets, based on the global and local maximum values of the silhouette metric. For salmonellosis, there were six clusters (Set 1) in the global maximum and 11 clusters (Set 2) in the local maximum (Table 2). The description of selected clusters consists of the average values of daily morbidity with their 95% confidence intervals, the average values of meteorological characteristics, and the number of days in the clusters belonging to each of four seasons, defined by three consecutive months (starting with December–February as winter). 

Cluster 5 in Set 1 has high disease counts (greater than 3 cases per day, representing the 98th percentile) mainly during summer days with a relatively high average temperature of 16.09 °C, low average humidity of 65.82%, and low pressure of 989.65 hPa. This cluster also picks 50 days and 12 days that were unseasonably warm for spring and fall, respectively. Set 2 consisted of eleven clusters for salmonellosis (Table 2). The two clusters with high counts of salmonellosis (on average 3 or more cases per day) were Cluster 4 and Cluster 10. Cluster 4 mainly contains days of the summer season (similarly to Cluster 5 in Set 1), while Cluster 10 includes 66 days the spring, summer and fall seasons with low relative humidity values of 48.32%. Thus, these clusters of high disease counts occurred during unseasonably warm days in spring and fall, and during summer days with relatively high average temperature, low average humidity, and low pressure and (Table 2).

**Figure 5:**
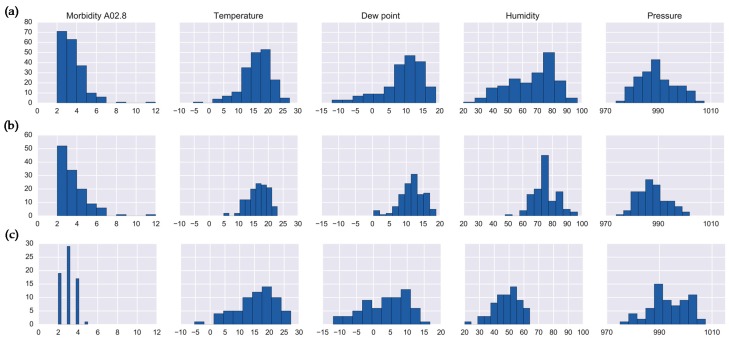
Histograms of salmonellosis daily counts and meteorological characteristics for: (**a**) cluster 5 (Set 1), (**b**) cluster 4 (Set 2), and (**c**) cluster 10 (Set 2).

We compared the histograms of the clusters identified in Sets 1 and 2 (Figure 5). The clusters with the highest number of cases (Cluster 5 in Set 1 and Cluster 4 in Set 2) showed very similar histograms (Figure 5a,b, respectively) and were identifiable with both the local and global maxima of the silhouette metric. Table 3 shows the interquartile range (IR) and coefficient of kurtosis of disease counts and meteorological characteristics for the clusters with high disease counts: Cluster 5 in Set 1 and Clusters 4 and 10 in Set 2. The IR values for disease counts show that in general the structure of clustering is similar, yet for weather parameters the IR values are smallest in Cluster 4, suggesting a dense and homogeneous arrangement of this cluster. The kurtosis values for Cluster 5 were consistently high, indicating a less defined or more diffused clustering. For Cluster 10, which was the smallest cluster by size, the kurtosis values were small, yet the IR values were high. This suggests that Cluster 10 was on average more diffuse with respect to temperature, dew point, and pressure parameters.

**Figure 6:**
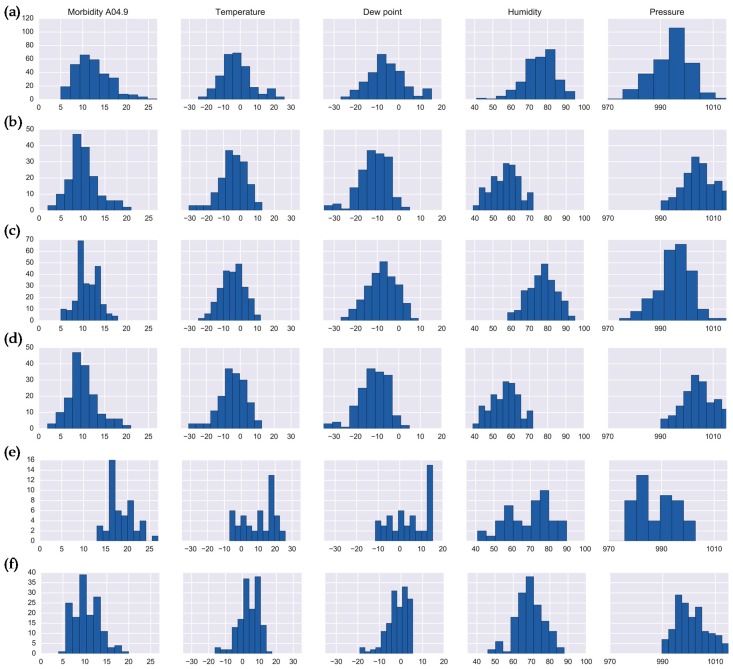
Histograms of enteritis daily counts and meteorological characteristics for: (**a**) Cluster 2 (Set 1); (**b**) Cluster 8, (Set 1); (**c**) Cluster 7 (Set 2); (**d**) Cluster 8 (Set 2); (**e**) Cluster 10 (Set 2); (**f**) Cluster 11 (Set 2).

For enteritis, there were eight clusters (Set 1) in the global maximum and 11 clusters (Set 2) in the local maximum (Table 4). The clustering results of Set 1 reveal two clusters, Cluster 2 and Cluster 8, with high disease counts, which were on average exceeding 10 cases per day (representing the 81st percentile). The histograms for these clusters are shown in Figure 6a,b. Cluster 2 includes primarily spring and winter days with an average temperature of −2.11 °C, relative humidity of 75.62%, and pressure of 993.98 hPa, which are locally considered as “warm winter” or “cold spring” days. Cluster 8 includes the days of the spring season (March and April), with high pressure values of 1004.68 hPa, low relative humidity of 56.44%, and relatively low temperature values of −4.6 °C. 

Clustering of enteritis in Set 2 revealed four clusters (Clusters 7, 8, 10, and 11) with high disease counts (on average above 10 cases per day). The histograms of the cluster features are shown in Figure 6c–f. Cluster 8 in Set 2 is identical to Cluster 8 in the Set 1 (Figure 6b,d). Clusters 7, 8, and 11 includes days that are unseasonably warm for local winters with an average daily temperature of −4.52 °C, pressure of 995.25 hPa, and humidity of 76.84%, yet are also colder than typical spring weather. Cluster 11 includes “very warm” winter days with an average temperature of 3.87 °C, high mean pressure values of 1000.93 hPa and average humidity of 68.93%. Cluster 2 from Set 1 and Cluster 10 from Set 2 contain days representing all four seasons, which indicates potentially sporadic outbreaks, deserving further evaluation.

Table 5 offers a comparison of clusters with a high incidence of enteritis for both sets based on measures of interquartile range and kurtosis. Cluster 8 in Set 1 and Set 2 are identical, which is explained by the mechanism of hierarchical clustering. Clusters 7 and 11 have the smallest values of interquartile range for all weather parameters, indicating a smaller dispersion of these features for the days in those clusters. Overall, Cluster 7 is the most homogeneous cluster in terms of meteorological factors and has the smallest values of kurtosis. Cluster 10 is the smallest cluster with only 47 days, yet it is a very noisy cluster that includes sporadic days with high disease counts. 

**Figure 7:**
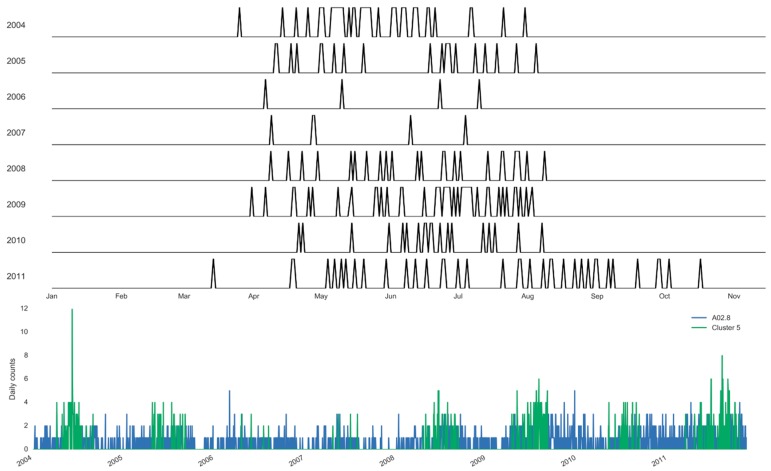
Sampling distribution of days selected annually and the corresponding time series for Cluster 5 of salmonellosis, A02.8 (Set 1) in Barnaul, Russia in 2004–2011.

**Figure 8:**
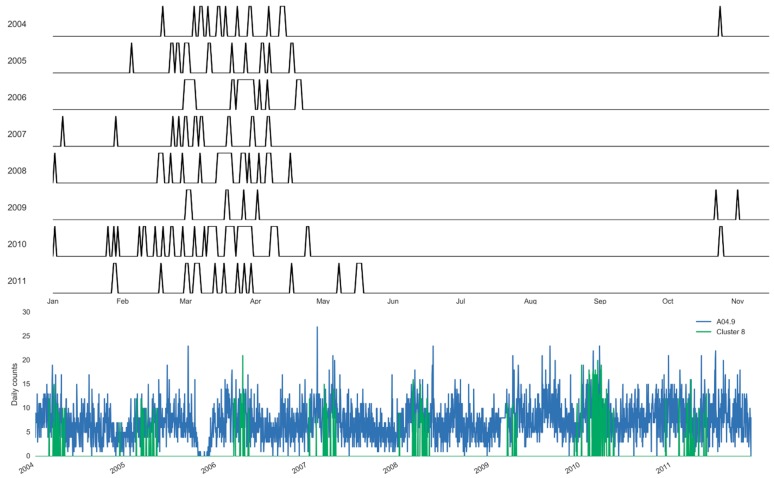
Sampling distribution of days selected annually and the corresponding time series for Cluster 8 of enteritis, A04.9 (Set 1) in Barnaul, Russia in 2004–2011.

Using the clustering approach, we estimate the specific days and the boundaries of each selected cluster (Figure 7 and Figure 8). Figure 7 illustrates the annual boundaries for the largest cluster constructed for salmonellosis, Cluster 5, along with the time series highlighting the days selected for the cluster. Cluster 5 was centered around early July (186th ± 21 days), supporting the marked 127 summer days of this cluster. Similarly, Figure 8 shows the annual boundaries for Cluster 8 constructed for non-specific enteritis with the annual centroids near late March and early April (95th ± 13 days). The peak timing estimated based on the results of the basic harmonic regression model (Model A, Table 6) confirms these findings. The seasonal increases for salmonellosis and enteritis were in early July (187th ± 7 days) and mid-April (103th ± 10 days), respectively. The sensitivity analysis demonstrates the stability of peak timing estimation (Table 6). 

**Figure 9:**
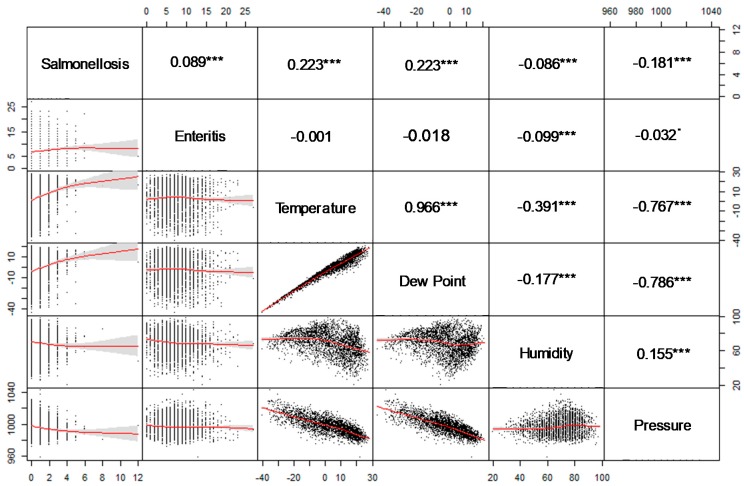
A scatter-plot matrix for health outcomes and weather variables and pair-wise Spearman correlation. *** *p* < 0.001, * *p* < 0.1.

The associations between ambient temperature or dewpoint and salmonellosis are relatively weak yet significant, which agrees with the salmonellosis summer peak timing (Figure 9). As expected for temperate climates, the associations between ambient temperature or dewpoint and barometric pressure are quite strong and inverse. The results of the univariate log-linear regression Model B shown in Table 7 agree with the agglomerative clustering results and Spearman correlation. The relative risks of an increase in daily salmonella counts associated with 10 °C in ambient temperature and dew point were 27.8% (CI 95%:22.8–33.0) and 30.9% (CI 95%:25.2–37.0), respectively. For both health outcomes, low humidity and barometric temperature were associated with an increase in disease counts. However, after adjusting for autocorrelation, seasonality, and effects of the day of the week, these results no longer hold (data not shown).

## 4. Discussion

The presented study illustrated a hierarchical agglomerative approach for clustering the time series data as allocation of days with similar values of meteorological factors and disease counts. We detected a well-defined cluster for salmonellosis, which occurred primarily during summer months and unusually warm days in spring. For the counts of enteritis, or non-specific bacterial intestinal infection, we detected two clusters during unseasonably warm winter days. The proposed method offers several important insights for understanding temporal variations and meteorological conditions favoring the transmission of enteric infections. The main advantage offered by the proposed techniques is its ability to create a composite of meteorological conditions similar to a rule of thumb to suspect a day with potentially high disease counts for a given location. This method can also tolerate low periodicity and autocorrelation, and thus be useful for unstable weather periods, especially during transitional seasons like spring and fall in temperate climates. The ability to identify the environmental predictors of infectious outbreaks, specifically a relevant set of weather conditions is essential for building predictive models to inform public policy [9,23]. Conventional weather forecasts can be used to test whether the proposed method has sufficient predictive capacity, especially in situations when the weather changes abruptly. These findings have major implications for understanding potential health impacts of climate change and extreme weather effects.

In comparison to classic harmonic regression models that offer the estimates of seasonal characteristics for well-defined periodic processes, the clustering approach can help in fine tuning for unseasonable weather. As weather instability has become more prominent with climate change, the predictive capacity of typical harmonic prediction that assumes a well-defined cyclical behavior is likely to diminish. Using the clustering approach, we can estimate the specific days and the boundaries of each selected cluster with high disease counts (as shown in Figure 7 and Figure 8). The boundaries of the cluster fluctuated depending on the number of days with the pre-specified weather pattern in each year. In fact, in the last year of the study period the upper boundary of the cluster was substantially shifted to the right, picking up the warm weather pattern during the unusual late summer and fall of 2011.

As expected, the traditional harmonic regression is not sensitive enough to capture departures from stable oscillations. The observed summer peak is typical for well documented seasonal increase in salmonellosis in temperate climates [6]. While the single summer peak in salmonellosis and the major spring peak in enteritis were detected by both methods, the harmonic regression missed the irregular spikes of salmonellosis in spring and spikes of non-specific enteritis occurred during summer months. For enteritis, the annual centroids of Cluster 8 fluctuated near late March and early April (95th ± 13 day of the year). Similarly, the harmonic regression model predicts the peak on the 103rd day of the year. These similarities reassure the potential for applying both methods simultaneously to better characterize the detected increase: by estimating seasonal peak timing offered by harmonic regression and by defining the homogeneity of a seasonal increase offered by the agglomerative clustering method. The classic log-linear regression models to assess the association between daily counts of infection and weather characteristics confirmed the relationships presented by the agglomerative clustering but offered low predictive capacity, with Model B having a variability below 10%. Even after adjusting for first order autocorrelation and weekly cycle, the traditional model was still insufficient to capture temporal variations associated with subtle changes in weather variables.

We anticipated that a comparison of the seasonal clusters in two health outcomes, salmonellosis and non-specific enteritis, will give us some clues whether some cases of non-specific enteritis were likely to be untested bacterial infections. We were able to make such assertions in our early study of medical claims [7]. We found that most nonspecific gastrointestinal diseases peak concurrently with viral enteritis, suggesting a lack of diagnostic testing for viruses, which may adversely affect the efficiency of prevention, surveillance, and treatment efforts. It is also possible that the seasonal increases in non-specific infections could be indicative of emerging strains that are rarely tested in routine settings. The detected partial overlap in two infections indicate that the suggested clustering approach could complement existing methodology for outbreak detection and characterization [23,24,25], and might have the potential to estimate the rate of under-reporting and provide recommendations to test for infections known to peak in certain times.

The suggested approach also offers characteristics to measure cluster compactness and homogeneity. We used the interquartile range and the kurtosis coefficient to estimate the features distribution of the obtained clusters. The interquartile range, which is a robust analogue of the variance, allows us to estimate the spread of the features of the objects in a cluster. Kurtosis allows us to estimate the sharpness of the distribution peak and the presence of “popular values” of features as well as the homogeneity or the flatness of the distribution. As the results show, splitting the data into smaller clusters can yield more compact subsets of data describing morbidity and weather factors. In fact, for salmonellosis Cluster 4 (Set 2) turned out to be more compact in comparison with Cluster 5 (Set 1) for weather factors: the values of interquartile range are smaller, while the excess value for pressure and relative humidity is higher. A similar situation is observed for bacterial infection, where Clusters 7 and 11 (Set 2) had lower interquartile range values as compared to the largest Cluster 2 (Set 1).

In studying the temporal variations in infectious disease, the outliers and missing data points often complicate the classical time series analysis. Typically, if there are missing values in the time series, then perhaps standard methods for imputing or recovering missing values, most suitable for time series, should be used at the development and application stages [26]. While in the presented example the proportion of missing data was low, it is possible that potential underreporting had resulted in overall reduced counts. Both HRMS and agglomerative clustering can tolerate a small fraction of values missing at random. However structural or systematic missingness might affect cluster detection, and the extent of such effect should be further investigated. 

For infectious diseases characterized by periods of outbreaks alternating with periods of low incidence, deciding whether a spike in fact is an outlier is difficult. It is unlikely that a true process governing the observed distribution is known, so we often rely on common assumption of Poisson or negative binomial distributions typical for time series of counts. In our example, only one value (12 cases of salmonellosis) warrants suspicion and when we tested its impact this single outlier did not affect the result of clustering. We argue that the understanding of how a substantial fraction of outliers might influence the clustering process, such repeating the analysis with and without outliers [27], or by applying robust methods of clustering [28], should be a standard practice.

The proposed example tackled the time series of infections only in one location and it would be valuable to know if the detected clusters will hold in cities with similar population structures and similar climates. The CliWaDIn database contains data for six Russian cities and this research will be further extended to test whether the number and characteristics of clusters vary geographically and also whether it is possible to build a “universal model” [29]. In addition to meteorological characteristics, any future analysis should consider water quality parameters, demographic structure, and social factors such as holidays, typical vacation times, workdays, and weekends. In our current model, we did not consider the incubation period or lag effects of a disease and included only weather characteristics for the day of disease counts. We envision that at the next stage an investigation of the lag structure and delayed effects is essential for revealing the patterns that might precede an outbreak [11]. The poor performance of traditional log-linear models could stem from potential non-linearity, auto-correlation, and presence of lagged effects [30]. The inclusion of additional adjustments to capture the delayed effects of weather variables on the health outcome could potentially improve the model fit. In fact, in 2008 we published a method that allowed us to quantify the time-distributed effect of exposure on a spread of infection with a known incubation period [11]. We modeled the health effect of high temperature on reported cryptosporidiosis caused by a pathogen with ~7-day incubation period and demonstrated the model’s ability to recover the effect with a nonlinear distributed lag structure. Future studies should determine a structure of the delay and consider various reasons for the delay (including an incubation period, a differential delay due to disease severity, an administrative delay due to testing and reporting procedures), and compare the delayed effects recovered by both log-linear model and machine learning methods with a broad range of lags and permutations for weather parameters. 

Understanding of temporal patterns along with accurate estimation of seasonal peak timing are essential for building reliable predictive models. Foodborne and waterborne enteric infections caused by pathogens sensitive to environmental conditions are difficult to track and control. While their seasonal peaks could be well synchronized, outbreaks triggered by high temperature may exhibit peak delays [7]. Improvements in surveillance system detection and reporting, implementation of public health policies along with environmental and climatic changes may result in shifts of seasonal peaks and amplitudes [10]. An investment into routine monitoring, tracking and forecasting of infections sensitive to environmental and climate conditions is reasonable and cost effective considering the damage cost of climate change [12,13,23]. While the proposed model is not designed to be continually updated with real time meteorological forecast because of peculiarities of the hierarchical clustering mechanism, future studies should explore opportunities for developing methods for near-term forecasting and early outbreak detection based on emerging methodology and data sources.

## 5. Conclusions

With the growing availability of routine disease monitoring, identification of environmental conditions that are predictive of disease occurrence requires novel ways to explore time-referenced data. Presenting this problem as an unsupervised machine learning task allowed us to detect and describe the features indicative of time periods with high counts of infections. We demonstrated the process of cluster identification and results of clustering by using two enteric infections that were likely transmitted by food and water, and were affected by metrological factors such as atmospheric temperature, barometric pressure, relative humidity, and dew point. We identified time periods with unseasonable meteorological conditions that “favor” infections. These findings had major implications for understanding the potential health impacts of climate change. The main research objective in identifying environmental predictors of infections should shift to designing robust algorithms to predict health outcomes based on meteorological, environmental, demographic parameters that govern the underlying dynamic system. 

## Figures and Tables

**Table 1:** Statistical characteristics for daily values of health outcomes and weather parameters.

Parameter	Mean	SD	Median	Minimum	Maximum	IR	Kurtosis
Salmonellosis (A02.8)	0.67	1.01	0.00	0.00	12.00	1.00	9.77
Enteritis (A04.9)	7.38	3.80	7.00	0.00	27.00	5.00	0.85
Temperature, °C	2.96	14.55	4.83	−40.16	27.81	22.61	−0.61
Dew point, °C	−2.74	12.88	−0.97	−44.16	20.69	18.60	−0.32
Humidity, %	68.56	12.82	70.00	20.00	98.00	18.00	−0.09
Pressure, hPa	997.02	10.44	996.25	958.11	1042.55	14.68	−0.01

SD: standard deviation; IR: interquartile range.

**Table 2:** Average values of clusters for salmonellosis, A02.8 and weather parameters.

Cluster	Daily Number of Cases	Confidence Interval for Number of Cases	Temperature, °C	Dew Point, °C	Humidity, %	Pressure, hPa	Seasonal Distribution, Number of Days				
							Spring	Summer	Autumn	Winter	Total Number of Days
**Set 1**											
Cluster 1	0.72	[0.66;0.77]	16.51	9.14	64.70	988.94	19	606	209	-	834
Cluster 2	0.48	[0.41;0.54]	−18.41	−22.28	70.26	1010.65	122	-	-	407	529
Cluster 3	0.27	[0.22;0.32]	1.16	−2.76	75.71	1000.56	-	-	489	-	489
Cluster 4	0.33	[0.27;0.38]	−4.86	−8.03	77.48	997.34	232	-	-	265	497
Cluster 5	3.10	[2.91;3.28]	16.09	8.87	65.82	989.65	50	127	12	-	189
Cluster 6	0.55	[0.47;0.63]	10.32	0.10	52.10	993.90	310	-		-	310
**Set 2**											
Cluster 1	0.27	[0.22;0.32]	1.17	−2.77	75.71	1000.57	-	-	489	-	489
Cluster 2	0.33	[0.27;0.38]	−4.86	−8.03	77.48	997.34	232	-	-	265	497
Cluster 3	0.55	[0.47;0.63]	10.32	0.10	52.10	993.90	310	-	-	-	310
Cluster 4	3.14	[2.88;3.41]	16.43	11.66	75.21	987.59	7	109	7	-	123
Cluster 5	0.54	[0.45;0.63]	−22.27	−25.27	74.44	1012.24	12	-	-	352	364
Cluster 6	1.52	[1.42;1.62]	11.92	4.01	61.37	994.62	-	50	134	-	184
Cluster 7	0.36	[0.30;0.43]	15.95	11.76	77.69	987.23	9	207	6	-	222
Cluster 8	0.33	[0.26;0.41]	−9.88	−15.71	61.05	1007.13	110	-	-	55	165
Cluster 9	0.02	[0.00;0.03]	17.28	8.06	57.48	989.78	-	168	69	-	237
Cluster 10	3.00	[2.81;3.19]	15.47	3.69	48.32	993.51	43	18	5	-	66
Cluster 11	1.23	[1.14;1.32]	20.65	12.41	61.80	984.41	10	181	-	-	191

Colored rows refer to clusters with the high disease counts.

**Table 3:** Clustering density and homogeneity for selected clusters with high salmonellosis counts.

Set	Cluster	Disease Counts		Temperature		Dew Point		Humidity		Pressure	
		IR	K	IR	K	IR	K	IR	K	IR	K
Set 1	Cluster 5	2.00	12.22	6.02	1.97	6.25	0.94	21.0	−0.44	9.04	−0.50
Set 2	Cluster 4	2.00	9.76	4.80	0.25	4.65	0.74	9.50	0.69	7.82	−0.44
	Cluster 10	2.00	−0.86	8.70	0.23	10.73	−0.60	13.00	0.03	9.98	−0.31

IR: interquartile range; K: coefficient of kurtosis.

**Table 4:** Average values of clusters for enteritis, A04.9 and weather parameters.

Cluster	Daily Number of Cases	Confidence Interval for Number of Cases	Temperature, °C	Dew Point, °C	Humidity, %	Pressure, hPa	Seasonal Distribution, Number of Days				
							Spring	Summer	Autumn	Winter	Total Number of Days
**Set 1**											
Cluster 1	7.21	[6.99;7.43]	17.60	11.10	68.47	987.33	24	702	48	-	774
Cluster 2	12.26	[11.82;12.70]	−2.11	−5.79	75.62	993.98	137	17	4	128	286
Cluster 3	7.62	[7.25;8.01]	8.43	1.02	62.56	997.18	-	-	332	-	332
Cluster 4	7.12	[6.82;7.44]	−21.02	−24.10	74.10	1011.89	17	-	-	399	416
Cluster 5	6.99	[6.67;7.32]	11.49	1.05	52.10	992.94	324	14	-	-	338
Cluster 6	5.01	[4.70;5.31]	−6.89	−9.76	78.64	1000.65	94	-	-	120	214
Cluster 7	4.17	[3.90;4.45]	−0.96	−4.27	78.06	1001.58	-	-	326		326
Cluster 8	10.08	[9.53;10.63]	−4.60	−11.86	56.44	1004.68	137	-	-	25	162
**Set 2**											
Cluster 1	7.13	[6.82;7.44]	−21.02	−24.10	74.10	1011.89	17	-	-	399	416
Cluster 2	6.99	[6.67;7.32]	11.49	1.05	52.10	992.94	324	14	-	-	338
Cluster 3	4.17	[3.90;4.45]	−0.96	−4.27	78.06	1001.58	-	-	326	-	326
Cluster 4	5.77	[5.51;6.02]	15.80	10.93	74.84	986.96	24	369	33	-	426
Cluster 5	5.45	[5.11;5.79]	12.14	3.18	57.37	994.13	-	-	183	-	183
Cluster 6	5.01	[4.70;5.31]	−6.89	−9.76	78.64	1000.65	94	-	-	120	214
Cluster 7	11.05	[10.72;11.38]	−4.52	−7.79	76.84	995.25	114	1	-	124	239
Cluster 8	10.08	[9.53;10.63]	−4.60	−11.86	56.44	1004.68	137	-	-	25	162
Cluster 9	8.99	[8.70;9.27]	19.81	11.30	60.67	987.80	-	333	15	-	348
Cluster 10	18.43	[17.58;19.27]	10.17	4.35	69.44	987.50	23	16	4	4	47
Cluster 11	10.30	[9.83;10.77]	3.87	−1.63	68.93	1000.93	-	-	-	149	149

Colored rows refer to clusters with the high disease counts.

**Table 5:** Clustering density and homogeneity for selected clusters with high enteritis counts.

Set	Cluster	Disease Counts		Temperature		Dew Point		Humidity		Pressure	
		IR	K	IR	K	IR	K	IR	K	IR	K
Set 1	Cluster 2	4.00	0.74	10.48	0.35	10.13	0.11	11.00	0.78	9.07	1.43
	Cluster 8	4.00	0.68	9.84	0.51	9.45	0.80	11.00	−0.63	8.06	−0.41
Set 2	Cluster 7	4.00	−0.38	9.79	−0.36	9.76	−0.37	10.00	−0.39	7.86	0.55
	Cluster 8	4.00	0.68	9.84	0.51	9.45	0.80	11.00	−0.63	8.06	−0.41
	Cluster 10	4.50	0.21	16.83	−1.14	15.49	−1.28	18.50	−0.63	11.65	1.31
	Cluster 11	4.00	0.17	7.86	0.67	6.25	1.51	9.00	0.18	8.00	−0.53

IR: interquartile range; K: coefficient of kurtosis.

**Table 6:** Sensitivity analysis of peak timing estimates based on harmonic regression models for health outcomes.

Infection	Peak Timing Estimates	Model A	Model A1	Model A2
Salmonellosis (A02.8)	RR (SE)	187.0 (7.4)	186.6 (8.6)	186.5 (8.6)
	LCI; UCI	[179.7;194.4]	[178.0;195.3]	[177.9;195.0]
Enteritis (A04.9)	RR (SE)	103.0 (9.5)	105.4 (12.3)	104.7 (11.9)
	LCI; UCI	[93.5;112.5]	[93.1;117.8]	[92.9;116.6]

RR: Risk Ratio; SE: Standard error; LCI & UCI: Lower and Upper confidence intervals.

**Table 7:** Results of log-linear regression model for health outcomes: estimates of relative risk associated with weather parameters.

Infection	Risk Estimates	Temperature	Dew Point	Humidity	Pressure
Salmonellosis (A02.8)	RR	1.278	1.309	0.915	0.772
	LCI; UCI	[1.228;1.330]	[1.252;1.370]	[0.877;0.954]	[0.731;0.815]
Enteritis (A04.9)	RR	0.994	0.986	0.965	0.986
	LCI; UCI	[0.981;1.007]	[0.972;1.001]	[0.950;0.979]	[0.968;1.004]

RR: relative risk; LCU and UCI: lower and upper confidence interval, respectively.

## Supplementary Materials

The following are available online at https://www.mdpi.com/1660-4601/16/12/2083/s1, Table S1. The daily counts of health outcomes and average daily values of temperature (°C), atmospheric pressure (hPa), relative humidity (%) and dew point (°C) from 1 January 2004 to 15 November 2011.
