# Supplementary material for: Agglomerative Clustering of Enteric Infections and Weather Parameters to Identify Seasonal Outbreaks in Cold Climates
Source: Int J Environ Res Public Health. 2019 Jun 12;16(12):2083. doi: 10.3390/ijerph16122083 (PMC6617417; doi:10.3390/ijerph16122083)
Supplement: Supplementary file 1 [file ijerph-16-02083-s001.zip › Supplemental Table S1.docx]

**Supplemental Table S1**. The daily counts of health outcomes: salmonellosis (A02.8) and enteritis (A04.9), and average daily values of temperature (^o^C), atmospheric pressure (hPa), relative humidity (%) and dew point (^o^C) from 1 January 2004 to 15 November 2011.

| **Date** | **A02.8** | **A04.9** | **Temperature ^o^C** | **Dew point ^о^С** | **Humidity**  **%** | **Pressure hPA** |
| --- | --- | --- | --- | --- | --- | --- |
| 01-01-2004 | 0 | 4 | -7.9 | -9.9 | 83 | 1005.5 |
| 01-02-2004 | 0 | 4 | -6.5 | -8.2 | 86 | 1004.9 |
| 01-03-2004 | 0 | 9 | -8.6 | -11.4 | 77 | 1005.0 |
| 01-04-2004 | 0 | 7 | -10.3 | -12.9 | 79 | 1005.6 |
| 01-05-2004 | 0 | 8 | -21.2 | -23.9 | 77 | 1012.5 |
| 01-06-2004 | 0 | 7 | -23.8 | -25.8 | 81 | 1008.1 |
| 01-07-2004 | 0 | 8 | -20.6 | -22.6 | 81 | 1006.3 |
| 01-08-2004 | 1 | 7 | -21.3 | -23.9 | 77 | 1004.4 |
| 01-09-2004 | 2 | 13 | -23.2 | -25.5 | 79 | 1007.8 |
| 01-10-2004 | 0 | 6 | -23.7 | -25.8 | 80 | 1011.0 |
| 01-11-2004 | 0 | 3 | -9.3 | -12.7 | 74 | 997.2 |
| 01-12-2004 | 0 | 9 | -6.2 | -9.0 | 78 | 996.5 |
| 01-13-2004 | 0 | 8 | -14.6 | -17.2 | 79 | 1009.3 |
| 01-14-2004 | 1 | 9 | -25.3 | -27.5 | 79 | 1017.9 |
| 01-15-2004 | 1 | 6 | -25.6 | -28.0 | 79 | 1015.9 |
| 01-16-2004 | 0 | 11 | -24.8 | -27.1 | 79 | 1013.9 |
| 01-17-2004 | 0 | 5 | -23.0 | -25.2 | 80 | 1016.9 |
| 01-18-2004 | 1 | 4 | -21.9 | -23.9 | 82 | 1014.9 |
| 01-19-2004 | 0 | 11 | -24.9 | -27.3 | 78 | 1013.3 |
| 01-20-2004 | 0 | 7 | -25.9 | -28.2 | 79 | 1012.2 |
| 01-21-2004 | 0 | 11 | -22.2 | -24.5 | 80 | 1010.1 |
| 01-22-2004 | 0 | 9 | -20.8 | -22.9 | 81 | 1008.9 |
| 01-23-2004 | 0 | 11 | -19.1 | -21.5 | 79 | 1009.0 |
| 01-24-2004 | 0 | 4 | -18.1 | -20.9 | 76 | 998.7 |
| 01-25-2004 | 0 | 9 | -14.1 | -17.8 | 71 | 993.3 |
| 01-26-2004 | 0 | 6 | -7.6 | -10.4 | 77 | 992.5 |
| 01-27-2004 | 0 | 10 | -12.5 | -15.2 | 79 | 991.5 |
| 01-28-2004 | 0 | 7 | -25.7 | -28.8 | 73 | 1003.4 |
| 01-29-2004 | 0 | 9 | -27.9 | -30.4 | 77 | 1014.2 |
| 01-30-2004 | 0 | 6 | -26.3 | -28.7 | 77 | 1019.0 |
| 01-31-2004 | 0 | 6 | -24.3 | -26.9 | 76 | 1021.4 |
| 02-01-2004 | 1 | 7 | -20.3 | -22.6 | 79 | 1020.4 |
| 02-02-2004 | 0 | 12 | -12.8 | -14.6 | 84 | 1017.5 |
| 02-03-2004 | 0 | 8 | -15.3 | -17.2 | 84 | 1017.5 |
| 02-04-2004 | 0 | 9 | -21.1 | -22.9 | 84 | 1015.7 |
| 02-05-2004 | 1 | 13 | -19.9 | -21.8 | 83 | 1010.3 |
| 02-06-2004 | 0 | 6 | -12.6 | -15.8 | 75 | 1005.6 |
| 02-07-2004 | 0 | 8 | -5.8 | -9.8 | 70 | 998.9 |
| 02-08-2004 | 0 | 7 | -5.2 | -8.8 | 73 | 994.8 |
| 02-09-2004 | 0 | 10 | 0.0 | -1.7 | 88 | 994.7 |
| 02-10-2004 | 0 | 13 | 0.7 | -0.6 | 90 | 991.8 |
| 02-11-2004 | 1 | 12 | -3.8 | -7.3 | 74 | 998.2 |
| 02-12-2004 | 0 | 12 | -4.1 | -7.4 | 75 | 998.6 |
| 02-13-2004 | 0 | 12 | -2.6 | -6.8 | 71 | 987.1 |
| 02-14-2004 | 0 | 12 | -10.2 | -13.8 | 71 | 1003.6 |
| 02-15-2004 | 1 | 6 | -8.7 | -11.6 | 77 | 996.7 |
| 02-16-2004 | 1 | 9 | -1.3 | -3.5 | 83 | 976.0 |
| 02-17-2004 | 0 | 15 | -8.0 | -12.9 | 67 | 982.8 |
| 02-18-2004 | 2 | 11 | -18.3 | -22.2 | 68 | 1001.3 |
| 02-19-2004 | 0 | 13 | -12.5 | -15.0 | 79 | 1002.8 |
| 02-20-2004 | 2 | 11 | -7.5 | -10.6 | 77 | 1004.9 |
| 02-21-2004 | 1 | 4 | -11.8 | -15.7 | 70 | 1003.8 |
| 02-22-2004 | 1 | 13 | -12.8 | -15.6 | 77 | 1002.3 |
| 02-23-2004 | 0 | 5 | -6.2 | -8.7 | 80 | 999.4 |
| 02-24-2004 | 0 | 11 | -6.4 | -9.6 | 75 | 1003.7 |
| 02-25-2004 | 2 | 8 | -4.3 | -9.2 | 65 | 998.5 |
| 02-26-2004 | 0 | 12 | -7.0 | -13.1 | 58 | 999.0 |
| 02-27-2004 | 2 | 9 | -11.6 | -15.6 | 70 | 1009.2 |
| 02-28-2004 | 1 | 9 | -9.5 | -14.4 | 66 | 1011.7 |
| 02-29-2004 | 1 | 5 | -11.1 | -14.1 | 77 | 1013.5 |
| 03-01-2004 | 0 | 13 | -7.7 | -10.0 | 81 | 1012.9 |
| 03-02-2004 | 0 | 8 | -6.6 | -8.9 | 82 | 1014.3 |
| 03-03-2004 | 0 | 8 | -5.2 | -8.2 | 78 | 1010.6 |
| 03-04-2004 | 1 | 8 | -5.1 | -10.0 | 69 | 1005.1 |
| 03-05-2004 | 0 | 6 | -5.1 | -8.8 | 76 | 1000.8 |
| 03-06-2004 | 0 | 4 | -0.9 | -6.1 | 69 | 996.5 |
| 03-07-2004 | 0 | 9 | 2.7 | -0.0 | 82 | 979.4 |
| 03-08-2004 | 1 | 6 | -5.9 | -9.1 | 75 | 986.0 |
| 03-09-2004 | 0 | 16 | -7.4 | -11.6 | 69 | 1000.3 |
| 03-10-2004 | 2 | 19 | -6.8 | -8.9 | 83 | 1000.9 |
| 03-11-2004 | 1 | 10 | -5.8 | -9.6 | 72 | 993.2 |
| 03-12-2004 | 0 | 12 | -4.5 | -8.3 | 73 | 984.8 |
| 03-13-2004 | 0 | 7 | -16.2 | -22.0 | 57 | 1001.1 |
| 03-14-2004 | 0 | 3 | -18.2 | -21.3 | 74 | 1009.3 |
| 03-15-2004 | 0 | 7 | -17.9 | -23.0 | 62 | 1016.6 |
| 03-16-2004 | 0 | 14 | -14.9 | -21.7 | 56 | 1013.2 |
| 03-17-2004 | 0 | 15 | -12.5 | -19.8 | 54 | 1006.9 |
| 03-18-2004 | 0 | 7 | -9.4 | -12.3 | 78 | 1005.1 |
| 03-19-2004 | 1 | 10 | -7.7 | -11.6 | 71 | 1006.3 |
| 03-20-2004 | 1 | 6 | -7.3 | -13.3 | 60 | 1002.1 |
| 03-21-2004 | 0 | 10 | -5.6 | -7.3 | 86 | 1003.9 |
| 03-22-2004 | 0 | 6 | -6.7 | -10.2 | 75 | 1004.3 |
| 03-23-2004 | 1 | 17 | -0.8 | -3.8 | 80 | 993.1 |
| 03-24-2004 | 1 | 7 | 0.6 | -0.8 | 89 | 980.2 |
| 03-25-2004 | 0 | 8 | -10.3 | -16.2 | 59 | 994.7 |
| 03-26-2004 | 1 | 11 | -13.1 | -18.2 | 63 | 1001.8 |
| 03-27-2004 | 1 | 4 | -13.9 | -19.7 | 58 | 1007.8 |
| 03-28-2004 | 0 | 8 | -11.4 | -16.2 | 65 | 1012.8 |
| 03-29-2004 | 0 | 10 | -2.6 | -9.9 | 55 | 998.6 |
| 03-30-2004 | 1 | 13 | -0.2 | -2.8 | 82 | 998.5 |
| 03-31-2004 | 1 | 8 | 1.5 | -0.3 | 87 | 994.1 |
| 04-01-2004 | 0 | 11 | 6.6 | -2.8 | 52 | 989.4 |
| 04-02-2004 | 0 | 9 | 3.8 | 1.8 | 86 | 994.9 |
| 04-03-2004 | 0 | 4 | 2.5 | -2.9 | 69 | 1004.0 |
| 04-04-2004 | 0 | 9 | 4.1 | -5.7 | 50 | 1006.6 |
| 04-05-2004 | 1 | 6 | 4.3 | -4.1 | 55 | 1003.4 |
| 04-06-2004 | 4 | 7 | 4.9 | 0.4 | 74 | 994.1 |
| 04-07-2004 | 2 | 7 | 1.6 | -2.9 | 72 | 1001.8 |
| 04-08-2004 | 1 | 10 | 3.7 | -1.1 | 71 | 994.5 |
| 04-09-2004 | 0 | 5 | -0.5 | -5.0 | 70 | 1001.0 |
| 04-10-2004 | 0 | 4 | -2.5 | -9.9 | 55 | 1009.8 |
| 04-11-2004 | 0 | 7 | -1.8 | -7.6 | 63 | 1010.3 |
| 04-12-2004 | 1 | 9 | 4.6 | 2.5 | 86 | 995.1 |
| 04-13-2004 | 0 | 13 | 4.6 | 3.2 | 90 | 989.5 |
| 04-14-2004 | 0 | 7 | 4.8 | 4.0 | 94 | 985.0 |
| 04-15-2004 | 0 | 17 | 8.5 | 6.4 | 86 | 978.3 |
| 04-16-2004 | 0 | 9 | 0.1 | -4.9 | 68 | 986.7 |
| 04-17-2004 | 0 | 4 | 0.3 | -7.3 | 57 | 992.9 |
| 04-18-2004 | 0 | 5 | 1.5 | -6.7 | 56 | 991.0 |
| 04-19-2004 | 1 | 9 | 1.0 | -2.2 | 79 | 988.2 |
| 04-20-2004 | 0 | 10 | 4.6 | -2.4 | 62 | 1000.8 |
| 04-21-2004 | 0 | 7 | 7.7 | -1.9 | 52 | 997.0 |
| 04-22-2004 | 1 | 3 | 3.6 | -3.2 | 62 | 997.7 |
| 04-23-2004 | 0 | 6 | 6.5 | -4.0 | 50 | 994.6 |
| 04-24-2004 | 0 | 6 | 11.6 | 2.1 | 53 | 992.1 |
| 04-25-2004 | 2 | 7 | 8.6 | 4.5 | 76 | 979.3 |
| 04-26-2004 | 2 | 8 | 0.2 | -8.4 | 53 | 994.5 |
| 04-27-2004 | 0 | 7 | -1.3 | -7.8 | 58 | 1000.2 |
| 04-28-2004 | 3 | 10 | 2.5 | -7.0 | 50 | 1003.2 |
| 04-29-2004 | 0 | 5 | 4.5 | -2.4 | 62 | 994.9 |
| 04-30-2004 | 0 | 7 | -0.1 | -8.6 | 53 | 1002.8 |
| 05-01-2004 | 0 | 2 | 4.3 | -7.5 | 43 | 1009.6 |
| 05-02-2004 | 0 | 10 | 13.9 | -2.0 | 33 | 1003.8 |
| 05-03-2004 | 1 | 3 | 17.6 | 1.0 | 34 | 997.5 |
| 05-04-2004 | 1 | 12 | 20.9 | -0.7 | 25 | 987.6 |
| 05-05-2004 | 4 | 12 | 16.2 | 4.1 | 48 | 981.4 |
| 05-06-2004 | 2 | 4 | 6.1 | 1.3 | 73 | 983.7 |
| 05-07-2004 | 0 | 9 | 4.4 | -0.9 | 69 | 987.2 |
| 05-08-2004 | 1 | 2 | 8.0 | -4.1 | 44 | 991.2 |
| 05-09-2004 | 1 | 4 | 10.0 | 0.4 | 54 | 987.3 |
| 05-10-2004 | 1 | 2 | 9.0 | -3.9 | 41 | 997.5 |
| 05-11-2004 | 2 | 1 | 17.6 | -0.8 | 30 | 993.5 |
| 05-12-2004 | 1 | 7 | 19.7 | 3.0 | 34 | 993.1 |
| 05-13-2004 | 1 | 10 | 18.8 | 5.6 | 44 | 997.9 |
| 05-14-2004 | 1 | 3 | 18.0 | 4.5 | 44 | 1000.3 |
| 05-15-2004 | 0 | 2 | 20.7 | 4.9 | 41 | 998.5 |
| 05-16-2004 | 1 | 4 | 23.7 | 6.9 | 37 | 996.1 |
| 05-17-2004 | 2 | 9 | 24.5 | 9.4 | 42 | 992.6 |
| 05-18-2004 | 2 | 4 | 25.9 | 11.0 | 42 | 985.9 |
| 05-19-2004 | 2 | 6 | 27.3 | 10.7 | 37 | 979.4 |
| 05-20-2004 | 1 | 8 | 22.8 | 12.9 | 54 | 982.6 |
| 05-21-2004 | 1 | 8 | 16.2 | 12.6 | 79 | 988.3 |
| 05-22-2004 | 0 | 4 | 14.5 | 11.0 | 80 | 992.0 |
| 05-23-2004 | 4 | 9 | 17.3 | 10.0 | 66 | 992.2 |
| 05-24-2004 | 2 | 9 | 13.3 | 9.1 | 76 | 993.6 |
| 05-25-2004 | 4 | 6 | 12.0 | 7.6 | 75 | 994.8 |
| 05-26-2004 | 4 | 7 | 15.7 | 4.5 | 51 | 1001.1 |
| 05-27-2004 | 2 | 4 | 16.4 | 4.6 | 48 | 1002.1 |
| 05-28-2004 | 3 | 5 | 19.5 | 8.2 | 51 | 1004.0 |
| 05-29-2004 | 4 | 7 | 21.3 | 10.5 | 54 | 998.0 |
| 05-30-2004 | 1 | 2 | 22.9 | 11.3 | 50 | 988.1 |
| 05-31-2004 | 1 | 0 | 17.0 | 6.7 | 52 | 992.3 |
| 06-01-2004 | 4 | 3 | 13.3 | 4.7 | 57 | 1001.3 |
| 06-02-2004 | 1 | 11 | 15.0 | 4.5 | 51 | 1003.9 |
| 06-03-2004 | 4 | 11 | 21.6 | 9.1 | 46 | 998.1 |
| 06-04-2004 | 3 | 6 | 25.8 | 9.5 | 38 | 989.1 |
| 06-05-2004 | 1 | 1 | 25.6 | 8.5 | 36 | 984.0 |
| 06-06-2004 | 1 | 8 | 19.1 | 12.2 | 65 | 989.1 |
| 06-07-2004 | 12 | 5 | 16.2 | 4.6 | 48 | 997.6 |
| 06-08-2004 | 6 | 4 | 16.3 | 7.9 | 59 | 997.3 |
| 06-09-2004 | 4 | 2 | 16.7 | 7.6 | 55 | 999.8 |
| 06-10-2004 | 3 | 7 | 16.3 | 9.8 | 67 | 997.6 |
| 06-11-2004 | 3 | 5 | 17.6 | 10.5 | 66 | 997.8 |
| 06-12-2004 | 3 | 2 | 17.5 | 12.1 | 73 | 997.0 |
| 06-13-2004 | 1 | 3 | 21.2 | 11.8 | 58 | 992.9 |
| 06-14-2004 | 0 | 8 | 23.0 | 13.9 | 58 | 988.1 |
| 06-15-2004 | 2 | 8 | 20.2 | 13.9 | 67 | 989.7 |
| 06-16-2004 | 4 | 15 | 23.5 | 13.0 | 54 | 990.3 |
| 06-17-2004 | 1 | 10 | 20.2 | 10.4 | 54 | 986.1 |
| 06-18-2004 | 2 | 7 | 21.3 | 11.1 | 54 | 988.5 |
| 06-19-2004 | 1 | 5 | 21.6 | 13.6 | 63 | 987.9 |
| 06-20-2004 | 2 | 5 | 23.1 | 13.0 | 56 | 985.3 |
| 06-21-2004 | 1 | 8 | 21.1 | 13.9 | 65 | 989.0 |
| 06-22-2004 | 0 | 8 | 20.6 | 12.0 | 60 | 985.2 |
| 06-23-2004 | 3 | 6 | 18.2 | 13.6 | 76 | 979.8 |
| 06-24-2004 | 3 | 5 | 14.1 | 13.2 | 94 | 982.2 |
| 06-25-2004 | 2 | 4 | 17.0 | 11.9 | 73 | 986.9 |
| 06-26-2004 | 1 | 3 | 17.8 | 14.2 | 80 | 988.4 |
| 06-27-2004 | 1 | 2 | 17.6 | 15.0 | 85 | 988.5 |
| 06-28-2004 | 3 | 7 | 20.5 | 15.9 | 75 | 987.4 |
| 06-29-2004 | 2 | 5 | 21.0 | 16.9 | 78 | 982.8 |
| 06-30-2004 | 2 | 9 | 21.0 | 16.2 | 75 | 982.5 |
| 07-01-2004 | 0 | 9 | 21.0 | 16.3 | 76 | 982.2 |
| 07-02-2004 | 1 | 6 | 19.8 | 16.2 | 81 | 984.7 |
| 07-03-2004 | 0 | 2 | 18.9 | 15.2 | 81 | 981.4 |
| 07-04-2004 | 4 | 7 | 12.5 | 8.5 | 77 | 986.6 |
| 07-05-2004 | 2 | 12 | 12.5 | 7.2 | 71 | 985.5 |
| 07-06-2004 | 2 | 5 | 13.4 | 8.8 | 74 | 990.0 |
| 07-07-2004 | 2 | 8 | 18.7 | 9.9 | 57 | 987.7 |
| 07-08-2004 | 1 | 9 | 20.8 | 13.8 | 65 | 983.7 |
| 07-09-2004 | 0 | 1 | 17.5 | 15.0 | 85 | 981.3 |
| 07-10-2004 | 0 | 1 | 21.4 | 15.2 | 69 | 983.5 |
| 07-11-2004 | 2 | 11 | 20.4 | 15.9 | 77 | 980.5 |
| 07-12-2004 | 2 | 5 | 21.2 | 16.5 | 76 | 985.3 |
| 07-13-2004 | 0 | 10 | 22.1 | 17.0 | 73 | 988.4 |
| 07-14-2004 | 0 | 8 | 20.7 | 17.0 | 80 | 986.1 |
| 07-15-2004 | 2 | 8 | 20.6 | 16.7 | 79 | 988.5 |
| 07-16-2004 | 0 | 9 | 21.8 | 16.5 | 73 | 990.7 |
| 07-17-2004 | 0 | 12 | 21.0 | 15.9 | 74 | 991.0 |
| 07-18-2004 | 2 | 5 | 20.9 | 14.1 | 68 | 988.5 |
| 07-19-2004 | 0 | 6 | 18.7 | 12.4 | 68 | 988.1 |
| 07-20-2004 | 0 | 7 | 17.0 | 13.4 | 80 | 988.2 |
| 07-21-2004 | 0 | 5 | 16.5 | 13.5 | 83 | 987.3 |
| 07-22-2004 | 0 | 6 | 17.1 | 13.1 | 79 | 991.0 |
| 07-23-2004 | 1 | 8 | 18.6 | 13.0 | 73 | 989.4 |
| 07-24-2004 | 1 | 5 | 18.6 | 16.1 | 85 | 985.1 |
| 07-25-2004 | 1 | 5 | 19.5 | 14.4 | 74 | 988.8 |
| 07-26-2004 | 1 | 9 | 19.4 | 13.3 | 70 | 993.9 |
| 07-27-2004 | 1 | 6 | 19.8 | 11.8 | 64 | 993.7 |
| 07-28-2004 | 0 | 7 | 20.6 | 12.4 | 62 | 990.1 |
| 07-29-2004 | 0 | 11 | 21.1 | 11.9 | 57 | 985.5 |
| 07-30-2004 | 0 | 3 | 20.5 | 11.0 | 56 | 983.6 |
| 07-31-2004 | 0 | 5 | 20.5 | 12.5 | 63 | 983.3 |
| 08-01-2004 | 1 | 5 | 19.3 | 11.6 | 63 | 983.3 |
| 08-02-2004 | 2 | 4 | 19.8 | 14.0 | 73 | 980.2 |
| 08-03-2004 | 2 | 6 | 20.6 | 15.9 | 77 | 978.5 |
| 08-04-2004 | 1 | 17 | 18.8 | 14.9 | 78 | 979.5 |
| 08-05-2004 | 0 | 11 | 16.0 | 13.6 | 86 | 987.3 |
| 08-06-2004 | 0 | 7 | 17.3 | 13.1 | 77 | 984.3 |
| 08-07-2004 | 0 | 6 | 11.0 | 6.2 | 74 | 988.6 |
| 08-08-2004 | 1 | 7 | 11.3 | 6.4 | 73 | 991.0 |
| 08-09-2004 | 0 | 9 | 12.3 | 7.2 | 72 | 995.3 |
| 08-10-2004 | 0 | 4 | 16.1 | 6.6 | 56 | 991.2 |
| 08-11-2004 | 1 | 3 | 17.2 | 11.6 | 71 | 990.4 |
| 08-12-2004 | 1 | 2 | 16.9 | 13.1 | 80 | 993.8 |
| 08-13-2004 | 0 | 7 | 22.7 | 14.3 | 61 | 992.3 |
| 08-14-2004 | 1 | 3 | 23.0 | 15.0 | 63 | 987.5 |
| 08-15-2004 | 0 | 0 | 23.2 | 13.6 | 57 | 984.7 |
| 08-16-2004 | 1 | 14 | 17.6 | 11.5 | 70 | 992.4 |
| 08-17-2004 | 0 | 9 | 19.2 | 12.6 | 68 | 989.2 |
| 08-18-2004 | 1 | 4 | 19.6 | 12.6 | 65 | 989.3 |
| 08-19-2004 | 2 | 5 | 15.4 | 8.6 | 68 | 991.5 |
| 08-20-2004 | 1 | 11 | 18.2 | 8.6 | 56 | 987.9 |
| 08-21-2004 | 1 | 2 | 15.0 | 8.9 | 70 | 983.3 |
| 08-22-2004 | 0 | 3 | 11.8 | 7.3 | 76 | 991.7 |
| 08-23-2004 | 0 | 7 | 12.0 | 6.3 | 71 | 997.2 |
| 08-24-2004 | 1 | 10 | 12.9 | 7.1 | 70 | 997.2 |
| 08-25-2004 | 1 | 7 | 12.9 | 6.2 | 68 | 998.8 |
| 08-26-2004 | 2 | 7 | 14.7 | 6.3 | 61 | 999.5 |
| 08-27-2004 | 1 | 5 | 14.4 | 5.9 | 60 | 995.7 |
| 08-28-2004 | 0 | 5 | 14.2 | 5.8 | 63 | 991.9 |
| 08-29-2004 | 1 | 3 | 17.1 | 7.5 | 58 | 987.9 |
| 08-30-2004 | 3 | 2 | 17.7 | 10.6 | 66 | 986.0 |
| 08-31-2004 | 0 | 6 | 13.8 | 11.5 | 86 | 988.4 |
| 09-01-2004 | 0 | 5 | 11.5 | 10.6 | 94 | 990.8 |
| 09-02-2004 | 0 | 6 | 12.7 | 7.8 | 74 | 993.7 |
| 09-03-2004 | 2 | 7 | 13.9 | 7.8 | 69 | 993.0 |
| 09-04-2004 | 1 | 1 | 16.9 | 8.9 | 61 | 993.3 |
| 09-05-2004 | 2 | 6 | 19.7 | 8.6 | 51 | 990.4 |
| 09-06-2004 | 2 | 7 | 18.8 | 11.1 | 63 | 987.7 |
| 09-07-2004 | 1 | 7 | 10.4 | 8.9 | 91 | 985.3 |
| 09-08-2004 | 1 | 5 | 13.1 | 6.2 | 64 | 988.0 |
| 09-09-2004 | 2 | 7 | 12.9 | 6.2 | 66 | 992.5 |
| 09-10-2004 | 0 | 11 | 12.5 | 4.4 | 61 | 996.7 |
| 09-11-2004 | 0 | 4 | 17.4 | 8.1 | 58 | 991.0 |
| 09-12-2004 | 0 | 3 | 18.1 | 8.0 | 55 | 979.6 |
| 09-13-2004 | 2 | 13 | 12.4 | 5.3 | 64 | 985.4 |
| 09-14-2004 | 1 | 2 | 8.2 | 5.7 | 84 | 995.4 |
| 09-15-2004 | 2 | 8 | 10.7 | 5.2 | 70 | 996.4 |
| 09-16-2004 | 0 | 5 | 12.9 | 6.0 | 66 | 998.5 |
| 09-17-2004 | 0 | 4 | 15.9 | 8.8 | 64 | 998.5 |
| 09-18-2004 | 0 | 1 | 18.4 | 8.9 | 56 | 997.4 |
| 09-19-2004 | 0 | 3 | 18.2 | 9.2 | 58 | 991.4 |
| 09-20-2004 | 1 | 5 | 16.6 | 9.1 | 64 | 987.3 |
| 09-21-2004 | 0 | 9 | 8.0 | 7.2 | 94 | 989.7 |
| 09-22-2004 | 0 | 5 | 7.3 | 5.9 | 90 | 997.1 |
| 09-23-2004 | 0 | 3 | 6.0 | 3.3 | 84 | 1003.1 |
| 09-24-2004 | 0 | 1 | 4.6 | -0.9 | 70 | 1005.0 |
| 09-25-2004 | 0 | 2 | 1.4 | -2.6 | 75 | 1010.6 |
| 09-26-2004 | 0 | 1 | 1.5 | -2.0 | 80 | 1002.4 |
| 09-27-2004 | 0 | 4 | 0.2 | -2.5 | 82 | 1002.5 |
| 09-28-2004 | 0 | 3 | 0.6 | -5.0 | 69 | 1014.0 |
| 09-29-2004 | 0 | 5 | 2.2 | -4.4 | 65 | 1014.4 |
| 09-30-2004 | 0 | 8 | 9.1 | -2.7 | 47 | 1006.5 |
| 10-01-2004 | 0 | 2 | 16.0 | 0.1 | 35 | 998.7 |
| 10-02-2004 | 0 | 5 | 15.6 | 4.2 | 49 | 993.6 |
| 10-03-2004 | 1 | 6 | 16.8 | 6.8 | 53 | 991.0 |
| 10-04-2004 | 0 | 8 | 18.1 | 2.7 | 39 | 989.6 |
| 10-05-2004 | 0 | 12 | 17.5 | 5.4 | 48 | 987.1 |
| 10-06-2004 | 0 | 8 | 10.7 | 7.3 | 80 | 989.5 |
| 10-07-2004 | 1 | 5 | 4.1 | 2.1 | 87 | 997.1 |
| 10-08-2004 | 0 | 9 | 2.8 | 0.9 | 88 | 996.6 |
| 10-09-2004 | 0 | 7 | 5.3 | 4.2 | 92 | 1002.6 |
| 10-10-2004 | 0 | 7 | 9.9 | 4.1 | 68 | 1002.4 |
| 10-11-2004 | 0 | 8 | 9.9 | 2.4 | 62 | 1001.1 |
| 10-12-2004 | 0 | 7 | 10.8 | 3.3 | 61 | 997.0 |
| 10-13-2004 | 0 | 11 | 13.9 | 3.1 | 50 | 995.2 |
| 10-14-2004 | 0 | 7 | 13.2 | 3.7 | 53 | 991.7 |
| 10-15-2004 | 0 | 4 | 3.1 | -1.2 | 74 | 992.6 |
| 10-16-2004 | 0 | 3 | 2.4 | -0.3 | 81 | 999.5 |
| 10-17-2004 | 0 | 2 | 0.0 | -1.0 | 92 | 1003.0 |
| 10-18-2004 | 1 | 13 | -3.5 | -6.7 | 77 | 1012.6 |
| 10-19-2004 | 3 | 6 | -1.6 | -5.6 | 73 | 1008.9 |
| 10-20-2004 | 0 | 6 | 1.2 | -1.6 | 81 | 1008.0 |
| 10-21-2004 | 0 | 8 | 3.8 | -5.9 | 49 | 999.6 |
| 10-22-2004 | 0 | 3 | -0.5 | -4.5 | 74 | 1002.8 |
| 10-23-2004 | 0 | 7 | -4.7 | -7.6 | 79 | 1011.6 |
| 10-24-2004 | 0 | 3 | 1.7 | -4.1 | 67 | 1002.4 |
| 10-25-2004 | 0 | 10 | 5.9 | 0.3 | 68 | 1002.7 |
| 10-26-2004 | 0 | 6 | 9.9 | -0.3 | 51 | 990.6 |
| 10-27-2004 | 0 | 4 | 0.7 | -2.4 | 80 | 992.9 |
| 10-28-2004 | 0 | 7 | -2.2 | -5.3 | 80 | 1003.3 |
| 10-29-2004 | 0 | 11 | 2.2 | -1.5 | 77 | 997.9 |
| 10-30-2004 | 0 | 8 | 4.3 | 2.1 | 85 | 986.8 |
| 10-31-2004 | 1 | 6 | 0.6 | -2.0 | 81 | 994.0 |
| 11-01-2004 | 0 | 6 | -3.9 | -7.1 | 76 | 996.8 |
| 11-02-2004 | 0 | 6 | -6.2 | -9.9 | 73 | 1005.7 |
| 11-03-2004 | 0 | 3 | -3.8 | -7.9 | 72 | 999.8 |
| 11-04-2004 | 0 | 5 | 0.7 | -2.1 | 80 | 988.6 |
| 11-05-2004 | 0 | 7 | -0.5 | -3.1 | 82 | 988.2 |
| 11-06-2004 | 0 | 6 | -1.3 | -2.6 | 90 | 988.2 |
| 11-07-2004 | 0 | 6 | -6.7 | -7.6 | 92 | 995.3 |
| 11-08-2004 | 0 | 2 | -11.9 | -13.8 | 84 | 1011.9 |
| 11-09-2004 | 1 | 6 | -8.5 | -11.5 | 76 | 1007.9 |
| 11-10-2004 | 1 | 7 | -2.6 | -4.2 | 88 | 994.2 |
| 11-11-2004 | 0 | 7 | -2.4 | -4.9 | 81 | 999.8 |
| 11-12-2004 | 0 | 5 | -1.0 | -2.8 | 87 | 1002.4 |
| 11-13-2004 | 0 | 4 | 0.6 | -1.4 | 87 | 1010.3 |
| 11-14-2004 | 0 | 2 | -2.5 | -5.5 | 77 | 1009.8 |
| 11-15-2004 | 0 | 4 | -4.3 | -7.8 | 75 | 1002.4 |
| 11-16-2004 | 1 | 1 | -1.5 | -6.3 | 67 | 994.6 |
| 11-17-2004 | 0 | 3 | -2.1 | -3.5 | 89 | 1002.0 |
| 11-18-2004 | 0 | 3 | -2.0 | -4.8 | 80 | 1001.6 |
| 11-19-2004 | 0 | 4 | 2.0 | -5.0 | 60 | 992.9 |
| 11-20-2004 | 0 | 0 | 0.0 | -1.9 | 85 | 1004.0 |
| 11-21-2004 | 0 | 4 | -1.2 | -2.5 | 90 | 1014.7 |
| 11-22-2004 | 0 | 5 | -3.3 | -4.9 | 87 | 1016.2 |
| 11-23-2004 | 1 | 2 | -4.4 | -9.1 | 68 | 1010.0 |
| 11-24-2004 | 0 | 7 | -6.6 | -8.8 | 83 | 1005.0 |
| 11-25-2004 | 0 | 5 | -6.2 | -9.9 | 73 | 999.7 |
| 11-26-2004 | 1 | 3 | 0.2 | -1.7 | 86 | 1002.2 |
| 11-27-2004 | 0 | 3 | 1.8 | -1.4 | 79 | 997.1 |
| 11-28-2004 | 0 | 2 | 0.5 | -3.5 | 73 | 988.8 |
| 11-29-2004 | 0 | 7 | -1.5 | -2.8 | 90 | 993.8 |
| 11-30-2004 | 0 | 3 | -6.8 | -10.0 | 75 | 1001.0 |
| 12-01-2004 | 0 | 3 | -6.9 | -8.8 | 84 | 997.4 |
| 12-02-2004 | 0 | 5 | -18.9 | -21.3 | 79 | 1011.5 |
| 12-03-2004 | 0 | 4 | -13.9 | -16.2 | 81 | 1002.2 |
| 12-04-2004 | 1 | 3 | -7.6 | -10.7 | 76 | 1002.3 |
| 12-05-2004 | 2 | 4 | -4.0 | -6.4 | 81 | 1001.5 |
| 12-06-2004 | 0 | 7 | -0.2 | -7.0 | 57 | 992.2 |
| 12-07-2004 | 0 | 4 | -10.6 | -13.7 | 75 | 998.4 |
| 12-08-2004 | 0 | 5 | -7.7 | -9.4 | 86 | 1000.3 |
| 12-09-2004 | 0 | 4 | -5.1 | -5.7 | 95 | 999.9 |
| 12-10-2004 | 1 | 1 | -12.9 | -14.8 | 84 | 1007.3 |
| 12-11-2004 | 0 | 1 | -5.3 | -5.9 | 95 | 995.4 |
| 12-12-2004 | 1 | 2 | -5.1 | -7.6 | 81 | 992.2 |
| 12-13-2004 | 0 | 7 | -8.0 | -9.7 | 86 | 1006.7 |
| 12-14-2004 | 2 | 4 | -8.8 | -10.2 | 88 | 998.1 |
| 12-15-2004 | 1 | 3 | -10.3 | -12.6 | 81 | 1003.0 |
| 12-16-2004 | 0 | 6 | -16.1 | -17.8 | 85 | 1008.7 |
| 12-17-2004 | 1 | 1 | -11.4 | -13.0 | 86 | 999.2 |
| 12-18-2004 | 0 | 2 | -11.1 | -12.7 | 86 | 998.3 |
| 12-19-2004 | 0 | 3 | -21.5 | -23.3 | 83 | 1005.3 |
| 12-20-2004 | 0 | 5 | -22.8 | -24.8 | 81 | 1008.5 |
| 12-21-2004 | 0 | 0 | -23.5 | -25.9 | 79 | 1008.6 |
| 12-22-2004 | 0 | 3 | -23.4 | -25.8 | 79 | 1006.3 |
| 12-23-2004 | 0 | 5 | -22.5 | -25.2 | 76 | 1011.4 |
| 12-24-2004 | 0 | 4 | -21.5 | -24.3 | 75 | 1015.5 |
| 12-25-2004 | 0 | 5 | -24.7 | -27.3 | 77 | 1019.2 |
| 12-26-2004 | 0 | 5 | -26.9 | -29.5 | 76 | 1020.6 |
| 12-27-2004 | 0 | 4 | -30.6 | -33.3 | 74 | 1017.7 |
| 12-28-2004 | 0 | 5 | -30.4 | -33.3 | 73 | 1019.8 |
| 12-29-2004 | 1 | 4 | -22.3 | -25.7 | 71 | 1017.0 |
| 12-30-2004 | 0 | 2 | -16.1 | -21.2 | 61 | 1007.0 |
| 12-31-2004 | 0 | 4 | -8.1 | -11.2 | 76 | 993.7 |
| 01-01-2005 | 0 | 6 | -16.8 | -18.8 | 82 | 998.0 |
| 01-02-2005 | 0 | 5 | -13.6 | -15.8 | 81 | 998.3 |
| 01-03-2005 | 0 | 4 | -9.7 | -13.2 | 73 | 999.6 |
| 01-04-2005 | 0 | 6 | -11.3 | -13.9 | 79 | 1003.2 |
| 01-05-2005 | 0 | 5 | -15.1 | -17.4 | 80 | 1009.0 |
| 01-06-2005 | 0 | 7 | -19.2 | -21.4 | 81 | 1011.8 |
| 01-07-2005 | 0 | 3 | -18.6 | -20.8 | 80 | 1006.0 |
| 01-08-2005 | 0 | 7 | -10.2 | -14.8 | 66 | 1003.5 |
| 01-09-2005 | 0 | 6 | -6.9 | -9.1 | 83 | 1005.6 |
| 01-10-2005 | 0 | 4 | -8.2 | -11.7 | 73 | 1005.8 |
| 01-11-2005 | 0 | 8 | -1.9 | -5.1 | 76 | 1003.0 |
| 01-12-2005 | 1 | 7 | -5.3 | -9.5 | 69 | 999.0 |
| 01-13-2005 | 3 | 2 | -2.4 | -6.0 | 73 | 1000.3 |
| 01-14-2005 | 0 | 4 | -5.8 | -7.4 | 86 | 1005.9 |
| 01-15-2005 | 0 | 4 | -8.1 | -8.9 | 93 | 1008.2 |
| 01-16-2005 | 0 | 0 | -4.8 | -5.6 | 92 | 1008.2 |
| 01-17-2005 | 0 | 6 | -5.4 | -7.4 | 84 | 1006.9 |
| 01-18-2005 | 1 | 9 | -8.8 | -13.4 | 66 | 1003.5 |
| 01-19-2005 | 0 | 12 | -12.3 | -14.7 | 80 | 1005.3 |
| 01-20-2005 | 0 | 2 | -16.7 | -18.5 | 84 | 999.6 |
| 01-21-2005 | 0 | 7 | -21.9 | -24.0 | 80 | 1002.5 |
| 01-22-2005 | 0 | 4 | -24.2 | -26.7 | 77 | 1011.6 |
| 01-23-2005 | 0 | 3 | -32.3 | -34.9 | 75 | 1016.8 |
| 01-24-2005 | 1 | 4 | -32.5 | -35.4 | 73 | 1014.4 |
| 01-25-2005 | 0 | 5 | -30.6 | -33.5 | 73 | 1014.1 |
| 01-26-2005 | 2 | 5 | -34.2 | -37.1 | 72 | 1017.2 |
| 01-27-2005 | 0 | 2 | -27.5 | -30.6 | 72 | 1017.4 |
| 01-28-2005 | 0 | 3 | -27.2 | -30.3 | 72 | 1017.7 |
| 01-29-2005 | 0 | 1 | -29.9 | -32.7 | 74 | 1022.9 |
| 01-30-2005 | 0 | 2 | -24.4 | -27.4 | 73 | 1020.5 |
| 01-31-2005 | 0 | 8 | -25.8 | -29.0 | 72 | 1017.6 |
| 02-01-2005 | 0 | 7 | -29.9 | -33.0 | 72 | 1020.0 |
| 02-02-2005 | 0 | 6 | -28.9 | -31.9 | 73 | 1013.8 |
| 02-03-2005 | 0 | 4 | -26.5 | -29.8 | 72 | 1011.7 |
| 02-04-2005 | 0 | 12 | -29.1 | -32.1 | 73 | 1015.9 |
| 02-05-2005 | 2 | 3 | -25.5 | -28.7 | 71 | 1014.8 |
| 02-06-2005 | 1 | 5 | -23.8 | -27.4 | 70 | 1011.8 |
| 02-07-2005 | 0 | 3 | -23.2 | -26.9 | 69 | 1011.3 |
| 02-08-2005 | 0 | 6 | -16.6 | -22.2 | 59 | 1010.3 |
| 02-09-2005 | 1 | 6 | -17.5 | -23.8 | 55 | 1009.2 |
| 02-10-2005 | 0 | 12 | -16.4 | -21.7 | 60 | 1007.0 |
| 02-11-2005 | 2 | 6 | -13.9 | -18.4 | 66 | 1000.8 |
| 02-12-2005 | 0 | 5 | -21.5 | -24.1 | 77 | 1005.6 |
| 02-13-2005 | 1 | 5 | -24.7 | -27.4 | 76 | 1014.4 |
| 02-14-2005 | 1 | 13 | -25.1 | -28.2 | 73 | 1020.0 |
| 02-15-2005 | 1 | 9 | -25.7 | -28.8 | 73 | 1023.9 |
| 02-16-2005 | 0 | 7 | -26.6 | -29.5 | 74 | 1024.6 |
| 02-17-2005 | 0 | 5 | -22.3 | -25.6 | 72 | 1020.5 |
| 02-18-2005 | 0 | 8 | -13.1 | -15.9 | 77 | 1008.9 |
| 02-19-2005 | 0 | 4 | -11.3 | -13.5 | 82 | 1005.2 |
| 02-20-2005 | 0 | 4 | -14.2 | -16.4 | 81 | 999.9 |
| 02-21-2005 | 1 | 6 | -22.9 | -25.0 | 81 | 1005.2 |
| 02-22-2005 | 0 | 8 | -21.2 | -24.1 | 76 | 1005.8 |
| 02-23-2005 | 0 | 7 | -20.3 | -23.7 | 73 | 1005.2 |
| 02-24-2005 | 0 | 8 | -18.6 | -22.6 | 68 | 1004.1 |
| 02-25-2005 | 1 | 2 | -15.4 | -21.2 | 58 | 1005.3 |
| 02-26-2005 | 1 | 7 | -14.6 | -18.0 | 73 | 1008.6 |
| 02-27-2005 | 0 | 2 | -11.0 | -14.0 | 76 | 1002.7 |
| 02-28-2005 | 0 | 5 | -7.8 | -11.7 | 72 | 1005.9 |
| 03-01-2005 | 0 | 12 | -8.9 | -11.2 | 83 | 1016.8 |
| 03-02-2005 | 0 | 11 | -7.3 | -11.8 | 71 | 1012.1 |
| 03-03-2005 | 1 | 12 | -6.8 | -13.6 | 58 | 1004.6 |
| 03-04-2005 | 0 | 13 | -2.2 | -7.0 | 69 | 1003.8 |
| 03-05-2005 | 1 | 9 | -9.5 | -17.2 | 50 | 1015.2 |
| 03-06-2005 | 1 | 7 | -7.8 | -17.5 | 43 | 1008.4 |
| 03-07-2005 | 0 | 4 | -2.2 | -7.5 | 66 | 986.7 |
| 03-08-2005 | 0 | 9 | -10.1 | -13.2 | 76 | 995.4 |
| 03-09-2005 | 1 | 10 | -13.1 | -16.9 | 71 | 1011.4 |
| 03-10-2005 | 1 | 9 | -8.1 | -14.2 | 60 | 1006.2 |
| 03-11-2005 | 0 | 9 | -3.6 | -8.5 | 67 | 998.8 |
| 03-12-2005 | 0 | 8 | -5.5 | -7.8 | 82 | 1005.7 |
| 03-13-2005 | 1 | 5 | -4.1 | -6.4 | 83 | 995.9 |
| 03-14-2005 | 2 | 10 | -8.0 | -12.0 | 70 | 1003.0 |
| 03-15-2005 | 0 | 8 | -5.9 | -8.5 | 80 | 1010.2 |
| 03-16-2005 | 0 | 4 | -5.7 | -10.0 | 71 | 1001.4 |
| 03-17-2005 | 1 | 10 | -1.8 | -5.6 | 74 | 998.6 |
| 03-18-2005 | 1 | 10 | -1.6 | -5.5 | 73 | 995.2 |
| 03-19-2005 | 1 | 6 | -1.2 | -5.6 | 72 | 987.4 |
| 03-20-2005 | 0 | 5 | -1.3 | -4.7 | 75 | 1002.4 |
| 03-21-2005 | 0 | 8 | 2.1 | -3.9 | 65 | 1002.2 |
| 03-22-2005 | 0 | 10 | 2.3 | -3.8 | 66 | 1000.4 |
| 03-23-2005 | 2 | 5 | -0.4 | -3.2 | 80 | 1004.5 |
| 03-24-2005 | 0 | 5 | 0.9 | -3.3 | 72 | 997.1 |
| 03-25-2005 | 0 | 6 | 0.0 | -3.9 | 73 | 993.3 |
| 03-26-2005 | 0 | 5 | 2.2 | -1.6 | 76 | 992.5 |
| 03-27-2005 | 1 | 4 | -1.0 | -6.1 | 67 | 1000.2 |
| 03-28-2005 | 0 | 7 | -0.8 | -5.2 | 71 | 1006.8 |
| 03-29-2005 | 0 | 8 | -0.2 | -4.2 | 75 | 1007.3 |
| 03-30-2005 | 1 | 15 | 2.1 | -3.5 | 67 | 997.7 |
| 03-31-2005 | 0 | 7 | 0.7 | -4.9 | 65 | 995.1 |
| 04-01-2005 | 1 | 6 | 0.2 | -5.6 | 65 | 994.5 |
| 04-02-2005 | 1 | 6 | -4.9 | -10.9 | 60 | 1000.6 |
| 04-03-2005 | 0 | 5 | -3.3 | -9.6 | 59 | 989.5 |
| 04-04-2005 | 0 | 7 | -3.8 | -9.8 | 61 | 990.5 |
| 04-05-2005 | 0 | 7 | -1.9 | -8.0 | 63 | 988.1 |
| 04-06-2005 | 1 | 8 | -6.2 | -9.2 | 78 | 993.2 |
| 04-07-2005 | 0 | 4 | -3.7 | -8.2 | 68 | 1004.5 |
| 04-08-2005 | 0 | 7 | 1.4 | -2.5 | 75 | 997.9 |
| 04-09-2005 | 1 | 7 | -6.2 | -14.8 | 49 | 1012.9 |
| 04-10-2005 | 1 | 5 | -0.8 | -5.6 | 69 | 1008.6 |
| 04-11-2005 | 0 | 7 | 3.2 | -1.0 | 74 | 1004.9 |
| 04-12-2005 | 1 | 5 | 4.7 | 0.9 | 78 | 1001.9 |
| 04-13-2005 | 0 | 8 | 8.4 | -0.1 | 58 | 992.7 |
| 04-14-2005 | 0 | 8 | 8.9 | 1.8 | 63 | 987.2 |
| 04-15-2005 | 1 | 12 | 2.6 | -1.6 | 74 | 997.0 |
| 04-16-2005 | 2 | 9 | 0.2 | -2.5 | 81 | 1001.6 |
| 04-17-2005 | 1 | 7 | 1.0 | -9.1 | 48 | 1011.8 |
| 04-18-2005 | 0 | 8 | 5.4 | -5.3 | 47 | 1003.9 |
| 04-19-2005 | 1 | 13 | 11.3 | -0.4 | 47 | 993.1 |
| 04-20-2005 | 1 | 9 | 13.7 | 5.2 | 59 | 994.5 |
| 04-21-2005 | 0 | 10 | 12.5 | 5.0 | 63 | 1000.8 |
| 04-22-2005 | 0 | 8 | 15.3 | 2.6 | 46 | 1000.7 |
| 04-23-2005 | 0 | 10 | 16.9 | 0.3 | 36 | 996.1 |
| 04-24-2005 | 0 | 7 | 16.3 | 4.6 | 50 | 997.5 |
| 04-25-2005 | 2 | 11 | 15.7 | 4.1 | 48 | 999.0 |
| 04-26-2005 | 4 | 8 | 18.6 | 7.2 | 48 | 989.3 |
| 04-27-2005 | 0 | 10 | 9.7 | 3.9 | 68 | 990.4 |
| 04-28-2005 | 2 | 4 | 3.0 | -2.7 | 66 | 991.8 |
| 04-29-2005 | 0 | 7 | 3.7 | -7.6 | 45 | 999.9 |
| 04-30-2005 | 0 | 4 | 7.5 | -4.6 | 42 | 994.9 |
| 05-01-2005 | 1 | 7 | 5.8 | -5.8 | 43 | 993.1 |
| 05-02-2005 | 2 | 8 | 1.4 | -9.8 | 43 | 1002.4 |
| 05-03-2005 | 3 | 10 | 2.6 | -10.4 | 39 | 1007.5 |
| 05-04-2005 | 1 | 5 | 7.8 | -8.0 | 33 | 997.2 |
| 05-05-2005 | 2 | 6 | 12.4 | 2.3 | 51 | 986.9 |
| 05-06-2005 | 4 | 6 | 16.1 | 2.8 | 43 | 984.3 |
| 05-07-2005 | 0 | 2 | 20.0 | 6.1 | 40 | 980.5 |
| 05-08-2005 | 0 | 10 | 15.4 | 4.8 | 50 | 990.7 |
| 05-09-2005 | 0 | 8 | 12.0 | -1.2 | 42 | 998.1 |
| 05-10-2005 | 0 | 3 | 12.5 | -0.2 | 43 | 995.1 |
| 05-11-2005 | 0 | 16 | 11.6 | 2.6 | 55 | 993.4 |
| 05-12-2005 | 0 | 11 | 10.0 | 0.1 | 52 | 994.2 |
| 05-13-2005 | 0 | 6 | 10.5 | -0.7 | 47 | 998.2 |
| 05-14-2005 | 1 | 5 | 11.0 | 2.6 | 57 | 999.7 |
| 05-15-2005 | 1 | 1 | 10.8 | 3.8 | 68 | 996.9 |
| 05-16-2005 | 1 | 13 | 13.1 | 1.6 | 49 | 1002.1 |
| 05-17-2005 | 0 | 6 | 17.7 | 3.7 | 41 | 1004.3 |
| 05-18-2005 | 3 | 13 | 19.3 | 6.7 | 46 | 1001.4 |
| 05-19-2005 | 3 | 8 | 18.6 | 9.6 | 57 | 996.0 |
| 05-20-2005 | 1 | 8 | 16.5 | 8.2 | 61 | 995.9 |
| 05-21-2005 | 0 | 6 | 17.7 | 8.4 | 56 | 995.0 |
| 05-22-2005 | 0 | 6 | 17.1 | 7.4 | 57 | 994.5 |
| 05-23-2005 | 0 | 6 | 11.0 | 0.4 | 51 | 1002.6 |
| 05-24-2005 | 1 | 8 | 14.9 | 2.9 | 47 | 993.2 |
| 05-25-2005 | 3 | 10 | 16.1 | 5.4 | 50 | 985.5 |
| 05-26-2005 | 2 | 5 | 9.2 | 5.5 | 79 | 981.4 |
| 05-27-2005 | 0 | 4 | 5.2 | -0.3 | 70 | 990.9 |
| 05-28-2005 | 1 | 5 | 10.4 | 0.3 | 53 | 993.7 |
| 05-29-2005 | 0 | 4 | 16.2 | 3.0 | 43 | 989.7 |
| 05-30-2005 | 3 | 8 | 18.8 | 8.7 | 54 | 980.4 |
| 05-31-2005 | 0 | 7 | 14.9 | 6.0 | 58 | 979.0 |
| 06-01-2005 | 1 | 8 | 10.3 | 7.9 | 85 | 977.4 |
| 06-02-2005 | 1 | 6 | 10.5 | 5.9 | 73 | 989.2 |
| 06-03-2005 | 0 | 8 | 12.8 | 5.1 | 61 | 997.4 |
| 06-04-2005 | 0 | 7 | 14.4 | 7.0 | 64 | 995.5 |
| 06-05-2005 | 0 | 8 | 14.8 | 8.8 | 69 | 994.0 |
| 06-06-2005 | 2 | 7 | 17.1 | 7.6 | 56 | 994.3 |
| 06-07-2005 | 1 | 8 | 19.4 | 9.2 | 55 | 992.6 |
| 06-08-2005 | 0 | 10 | 20.9 | 10.5 | 55 | 992.1 |
| 06-09-2005 | 4 | 9 | 23.5 | 12.9 | 54 | 989.2 |
| 06-10-2005 | 0 | 2 | 17.8 | 9.1 | 58 | 988.4 |
| 06-11-2005 | 1 | 5 | 20.2 | 10.8 | 57 | 988.8 |
| 06-12-2005 | 1 | 11 | 23.1 | 12.1 | 51 | 987.5 |
| 06-13-2005 | 0 | 9 | 23.7 | 11.4 | 47 | 984.8 |
| 06-14-2005 | 1 | 19 | 22.9 | 15.3 | 63 | 982.7 |
| 06-15-2005 | 1 | 10 | 21.6 | 14.2 | 64 | 982.2 |
| 06-16-2005 | 1 | 10 | 20.8 | 14.8 | 69 | 985.9 |
| 06-17-2005 | 1 | 12 | 21.1 | 14.6 | 69 | 983.4 |
| 06-18-2005 | 0 | 6 | 16.1 | 10.5 | 71 | 984.7 |
| 06-19-2005 | 1 | 12 | 17.6 | 8.6 | 56 | 982.7 |
| 06-20-2005 | 1 | 5 | 15.4 | 10.9 | 75 | 988.6 |
| 06-21-2005 | 0 | 9 | 20.6 | 11.0 | 56 | 992.1 |
| 06-22-2005 | 0 | 16 | 23.0 | 14.1 | 59 | 988.3 |
| 06-23-2005 | 0 | 7 | 25.9 | 15.5 | 54 | 980.5 |
| 06-24-2005 | 0 | 5 | 23.4 | 14.9 | 59 | 971.5 |
| 06-25-2005 | 2 | 4 | 18.8 | 10.8 | 61 | 974.3 |
| 06-26-2005 | 1 | 11 | 15.8 | 12.1 | 79 | 974.4 |
| 06-27-2005 | 0 | 6 | 17.1 | 13.0 | 78 | 979.8 |
| 06-28-2005 | 2 | 6 | 19.5 | 13.7 | 71 | 983.4 |
| 06-29-2005 | 0 | 5 | 21.1 | 13.0 | 62 | 988.8 |
| 06-30-2005 | 1 | 8 | 21.2 | 11.9 | 59 | 990.8 |
| 07-01-2005 | 0 | 9 | 23.1 | 14.4 | 61 | 988.2 |
| 07-02-2005 | 0 | 5 | 23.5 | 15.4 | 63 | 985.3 |
| 07-03-2005 | 0 | 7 | 21.3 | 16.2 | 74 | 985.5 |
| 07-04-2005 | 1 | 8 | 23.7 | 15.0 | 61 | 985.2 |
| 07-05-2005 | 0 | 6 | 26.0 | 16.2 | 57 | 983.9 |
| 07-06-2005 | 0 | 9 | 24.4 | 17.2 | 66 | 986.7 |
| 07-07-2005 | 1 | 7 | 24.6 | 14.4 | 57 | 986.4 |
| 07-08-2005 | 0 | 8 | 27.8 | 19.2 | 62 | 982.5 |
| 07-09-2005 | 0 | 8 | 25.8 | 20.7 | 74 | 981.8 |
| 07-10-2005 | 0 | 11 | 20.6 | 12.8 | 63 | 986.0 |
| 07-11-2005 | 2 | 10 | 19.1 | 9.1 | 53 | 987.7 |
| 07-12-2005 | 0 | 12 | 19.3 | 11.1 | 60 | 986.1 |
| 07-13-2005 | 4 | 4 | 22.7 | 13.2 | 56 | 978.8 |
| 07-14-2005 | 0 | 5 | 21.5 | 10.9 | 54 | 979.3 |
| 07-15-2005 | 2 | 7 | 19.4 | 9.9 | 57 | 982.6 |
| 07-16-2005 | 0 | 8 | 19.2 | 10.9 | 61 | 983.8 |
| 07-17-2005 | 1 | 7 | 17.7 | 13.2 | 76 | 987.5 |
| 07-18-2005 | 1 | 7 | 19.3 | 14.6 | 75 | 989.4 |
| 07-19-2005 | 2 | 13 | 16.3 | 12.9 | 82 | 990.0 |
| 07-20-2005 | 0 | 6 | 15.9 | 13.1 | 85 | 990.2 |
| 07-21-2005 | 3 | 4 | 16.7 | 14.6 | 87 | 988.8 |
| 07-22-2005 | 2 | 7 | 18.2 | 14.6 | 80 | 988.7 |
| 07-23-2005 | 3 | 6 | 19.4 | 14.2 | 75 | 988.7 |
| 07-24-2005 | 0 | 8 | 21.6 | 14.7 | 68 | 987.2 |
| 07-25-2005 | 0 | 8 | 23.4 | 16.1 | 66 | 985.0 |
| 07-26-2005 | 2 | 3 | 20.4 | 17.7 | 85 | 984.0 |
| 07-27-2005 | 0 | 8 | 20.4 | 17.8 | 85 | 982.8 |
| 07-28-2005 | 1 | 9 | 20.0 | 17.7 | 87 | 982.7 |
| 07-29-2005 | 1 | 11 | 20.7 | 17.1 | 81 | 982.2 |
| 07-30-2005 | 1 | 6 | 22.4 | 17.3 | 74 | 982.8 |
| 07-31-2005 | 0 | 10 | 22.9 | 16.7 | 70 | 983.2 |
| 08-01-2005 | 1 | 14 | 25.1 | 17.7 | 65 | 983.5 |
| 08-02-2005 | 2 | 7 | 27.0 | 17.7 | 60 | 982.3 |
| 08-03-2005 | 2 | 10 | 25.1 | 18.3 | 68 | 982.9 |
| 08-04-2005 | 1 | 12 | 21.2 | 18.5 | 85 | 983.6 |
| 08-05-2005 | 2 | 6 | 20.8 | 18.8 | 89 | 986.0 |
| 08-06-2005 | 0 | 6 | 21.5 | 19.1 | 86 | 987.4 |
| 08-07-2005 | 1 | 7 | 21.5 | 15.6 | 70 | 984.6 |
| 08-08-2005 | 2 | 8 | 19.9 | 13.6 | 68 | 984.1 |
| 08-09-2005 | 1 | 4 | 19.6 | 12.9 | 67 | 985.0 |
| 08-10-2005 | 2 | 10 | 14.0 | 10.5 | 80 | 985.2 |
| 08-11-2005 | 0 | 6 | 11.5 | 6.0 | 70 | 988.6 |
| 08-12-2005 | 0 | 5 | 11.6 | 6.0 | 70 | 993.8 |
| 08-13-2005 | 0 | 5 | 9.8 | 7.7 | 87 | 997.7 |
| 08-14-2005 | 1 | 4 | 11.3 | 6.0 | 73 | 1003.2 |
| 08-15-2005 | 1 | 7 | 17.4 | 8.4 | 57 | 998.4 |
| 08-16-2005 | 3 | 4 | 20.6 | 12.8 | 63 | 992.8 |
| 08-17-2005 | 0 | 9 | 21.4 | 13.2 | 64 | 989.8 |
| 08-18-2005 | 1 | 8 | 21.6 | 13.3 | 62 | 987.5 |
| 08-19-2005 | 1 | 4 | 21.9 | 12.6 | 59 | 987.2 |
| 08-20-2005 | 1 | 11 | 22.6 | 13.3 | 59 | 985.4 |
| 08-21-2005 | 2 | 9 | 25.5 | 12.5 | 46 | 979.5 |
| 08-22-2005 | 1 | 10 | 24.0 | 10.4 | 43 | 977.5 |
| 08-23-2005 | 0 | 13 | 14.9 | 10.7 | 77 | 984.1 |
| 08-24-2005 | 1 | 5 | 13.5 | 11.7 | 89 | 988.3 |
| 08-25-2005 | 1 | 7 | 14.6 | 11.9 | 84 | 988.8 |
| 08-26-2005 | 3 | 4 | 12.6 | 11.7 | 94 | 987.5 |
| 08-27-2005 | 0 | 5 | 15.5 | 13.6 | 88 | 988.3 |
| 08-28-2005 | 0 | 5 | 14.9 | 9.6 | 73 | 997.1 |
| 08-29-2005 | 0 | 7 | 15.1 | 10.5 | 76 | 995.8 |
| 08-30-2005 | 0 | 6 | 16.3 | 10.4 | 71 | 992.8 |
| 08-31-2005 | 0 | 7 | 16.4 | 10.1 | 68 | 991.0 |
| 09-01-2005 | 0 | 8 | 14.6 | 9.2 | 72 | 995.6 |
| 09-02-2005 | 0 | 7 | 16.2 | 9.9 | 69 | 999.1 |
| 09-03-2005 | 1 | 6 | 16.5 | 10.9 | 73 | 997.9 |
| 09-04-2005 | 0 | 3 | 19.5 | 10.6 | 58 | 992.3 |
| 09-05-2005 | 3 | 14 | 18.2 | 12.6 | 70 | 985.0 |
| 09-06-2005 | 1 | 23 | 16.7 | 12.8 | 78 | 982.8 |
| 09-07-2005 | 0 | 7 | 16.2 | 6.1 | 53 | 984.3 |
| 09-08-2005 | 0 | 6 | 12.2 | 7.2 | 73 | 988.6 |
| 09-09-2005 | 2 | 11 | 10.2 | 5.1 | 71 | 993.2 |
| 09-10-2005 | 0 | 5 | 14.7 | 6.7 | 60 | 990.3 |
| 09-11-2005 | 1 | 5 | 16.0 | 10.9 | 73 | 988.7 |
| 09-12-2005 | 1 | 7 | 12.0 | 6.5 | 71 | 996.2 |
| 09-13-2005 | 0 | 9 | 8.3 | 3.8 | 74 | 997.3 |
| 09-14-2005 | 0 | 4 | 6.6 | -0.5 | 64 | 1002.4 |
| 09-15-2005 | 0 | 9 | 6.0 | -0.5 | 68 | 1003.0 |
| 09-16-2005 | 0 | 7 | 8.8 | -0.3 | 57 | 1000.3 |
| 09-17-2005 | 0 | 4 | 9.7 | 0.8 | 56 | 999.4 |
| 09-18-2005 | 2 | 6 | 10.3 | 0.7 | 55 | 1000.5 |
| 09-19-2005 | 1 | 13 | 10.0 | 1.3 | 60 | 996.6 |
| 09-20-2005 | 1 | 9 | 9.7 | 1.5 | 61 | 996.5 |
| 09-21-2005 | 0 | 8 | 12.0 | 1.9 | 54 | 993.0 |
| 09-22-2005 | 0 | 2 | 9.3 | 3.7 | 71 | 990.6 |
| 09-23-2005 | 0 | 4 | 9.4 | 2.3 | 62 | 996.8 |
| 09-24-2005 | 0 | 7 | 10.3 | 4.1 | 67 | 993.1 |
| 09-25-2005 | 0 | 5 | 8.3 | 6.0 | 85 | 986.1 |
| 09-26-2005 | 2 | 6 | 5.2 | 2.7 | 84 | 997.0 |
| 09-27-2005 | 0 | 4 | 4.2 | -1.0 | 70 | 1006.6 |
| 09-28-2005 | 0 | 4 | 8.9 | 2.0 | 62 | 1000.8 |
| 09-29-2005 | 1 | 4 | 7.1 | 4.2 | 82 | 1000.8 |
| 09-30-2005 | 0 | 2 | 7.3 | 2.6 | 73 | 1010.7 |
| 10-01-2005 | 0 | 7 | 9.7 | 4.4 | 72 | 1009.9 |
| 10-02-2005 | 0 | 3 | 9.8 | 4.2 | 71 | 1000.9 |
| 10-03-2005 | 2 | 16 | 8.0 | 3.3 | 73 | 993.7 |
| 10-04-2005 | 0 | 5 | 4.6 | 0.4 | 74 | 1002.5 |
| 10-05-2005 | 0 | 5 | 5.0 | 0.3 | 73 | 1003.1 |
| 10-06-2005 | 1 | 6 | 10.1 | 1.3 | 57 | 996.4 |
| 10-07-2005 | 0 | 4 | 11.8 | 2.0 | 52 | 992.3 |
| 10-08-2005 | 0 | 2 | 10.4 | 2.0 | 57 | 988.2 |
| 10-09-2005 | 0 | 5 | 4.1 | 0.1 | 76 | 989.7 |
| 10-10-2005 | 1 | 2 | 1.2 | -0.9 | 85 | 1002.5 |
| 10-11-2005 | 0 | 3 | 0.7 | -5.9 | 62 | 1016.2 |
| 10-12-2005 | 0 | 4 | 2.1 | -5.4 | 57 | 1016.1 |
| 10-13-2005 | 1 | 3 | 5.1 | -4.6 | 49 | 1010.4 |
| 10-14-2005 | 0 | 3 | 7.2 | 0.2 | 62 | 1005.1 |
| 10-15-2005 | 0 | 1 | 5.6 | 1.6 | 76 | 1002.3 |
| 10-16-2005 | 0 | 0 | 4.8 | 1.4 | 80 | 1002.3 |
| 10-17-2005 | 0 | 0 | 8.9 | 2.3 | 66 | 1000.1 |
| 10-18-2005 | 0 | 0 | 6.1 | 3.9 | 85 | 999.0 |
| 10-19-2005 | 0 | 0 | 4.4 | 3.0 | 90 | 1004.4 |
| 10-20-2005 | 0 | 1 | 10.1 | -0.7 | 49 | 999.5 |
| 10-21-2005 | 0 | 0 | 13.4 | -0.6 | 38 | 994.5 |
| 10-22-2005 | 0 | 0 | 13.4 | -0.7 | 38 | 992.2 |
| 10-23-2005 | 0 | 1 | 9.1 | 1.0 | 58 | 990.2 |
| 10-24-2005 | 0 | 0 | 3.0 | 0.8 | 85 | 998.6 |
| 10-25-2005 | 0 | 1 | 5.3 | 0.5 | 71 | 1002.8 |
| 10-26-2005 | 0 | 0 | 5.6 | -2.1 | 60 | 1003.4 |
| 10-27-2005 | 0 | 0 | 6.6 | -0.5 | 63 | 996.6 |
| 10-28-2005 | 0 | 0 | 1.5 | -0.2 | 88 | 1003.8 |
| 10-29-2005 | 0 | 0 | 1.9 | -0.9 | 82 | 997.7 |
| 10-30-2005 | 0 | 0 | -2.2 | -6.0 | 75 | 995.8 |
| 10-31-2005 | 0 | 0 | -0.6 | -2.9 | 83 | 987.4 |
| 11-01-2005 | 0 | 0 | 0.8 | -0.7 | 89 | 986.7 |
| 11-02-2005 | 0 | 0 | 0.8 | -0.7 | 89 | 985.9 |
| 11-03-2005 | 0 | 0 | -4.5 | -11.5 | 55 | 996.4 |
| 11-04-2005 | 0 | 0 | -2.7 | -6.9 | 71 | 997.3 |
| 11-05-2005 | 0 | 0 | 0.5 | -1.5 | 86 | 994.9 |
| 11-06-2005 | 0 | 0 | 1.1 | -0.7 | 87 | 997.5 |
| 11-07-2005 | 0 | 0 | 0.6 | -2.2 | 81 | 983.8 |
| 11-08-2005 | 0 | 0 | -1.5 | -5.4 | 72 | 991.3 |
| 11-09-2005 | 0 | 0 | -1.0 | -4.8 | 73 | 997.8 |
| 11-10-2005 | 0 | 1 | -4.0 | -6.8 | 78 | 999.2 |
| 11-11-2005 | 0 | 0 | -8.6 | -10.8 | 82 | 1008.0 |
| 11-12-2005 | 0 | 0 | -3.8 | -6.3 | 80 | 1008.7 |
| 11-13-2005 | 0 | 0 | -0.5 | -2.8 | 85 | 1007.9 |
| 11-14-2005 | 0 | 0 | -3.2 | -4.2 | 92 | 1005.1 |
| 11-15-2005 | 0 | 0 | -1.6 | -4.3 | 80 | 1003.9 |
| 11-16-2005 | 0 | 0 | -3.9 | -5.9 | 84 | 1005.0 |
| 11-17-2005 | 0 | 0 | -2.7 | -4.4 | 87 | 1010.0 |
| 11-18-2005 | 0 | 0 | -9.4 | -12.1 | 79 | 1018.3 |
| 11-19-2005 | 0 | 0 | -15.7 | -17.6 | 83 | 1017.8 |
| 11-20-2005 | 0 | 0 | -15.5 | -17.6 | 82 | 1018.4 |
| 11-21-2005 | 0 | 1 | -14.8 | -17.0 | 82 | 1014.9 |
| 11-22-2005 | 1 | 0 | -13.2 | -15.8 | 79 | 1008.5 |
| 11-23-2005 | 0 | 0 | -10.2 | -14.4 | 70 | 997.8 |
| 11-24-2005 | 0 | 1 | -7.3 | -10.2 | 78 | 992.6 |
| 11-25-2005 | 1 | 2 | -10.1 | -12.4 | 81 | 996.1 |
| 11-26-2005 | 0 | 0 | -4.0 | -5.6 | 86 | 1002.2 |
| 11-27-2005 | 0 | 0 | -3.0 | -5.7 | 80 | 1004.9 |
| 11-28-2005 | 1 | 0 | -5.7 | -8.5 | 79 | 1003.5 |
| 11-29-2005 | 0 | 4 | -3.4 | -5.1 | 86 | 1008.1 |
| 11-30-2005 | 0 | 0 | -5.2 | -6.2 | 91 | 1008.8 |
| 12-01-2005 | 0 | 1 | -14.4 | -16.8 | 80 | 1012.9 |
| 12-02-2005 | 0 | 4 | -16.3 | -18.0 | 85 | 1019.3 |
| 12-03-2005 | 0 | 2 | -14.8 | -17.8 | 76 | 1011.8 |
| 12-04-2005 | 1 | 1 | -17.1 | -21.0 | 70 | 1004.6 |
| 12-05-2005 | 1 | 8 | -9.7 | -11.9 | 81 | 1002.7 |
| 12-06-2005 | 0 | 2 | -8.7 | -10.9 | 82 | 1004.3 |
| 12-07-2005 | 1 | 4 | -14.8 | -16.9 | 82 | 1008.1 |
| 12-08-2005 | 0 | 4 | -24.2 | -26.5 | 78 | 1015.7 |
| 12-09-2005 | 0 | 2 | -24.1 | -26.6 | 77 | 1023.9 |
| 12-10-2005 | 0 | 4 | -25.5 | -28.0 | 77 | 1028.0 |
| 12-11-2005 | 0 | 5 | -29.8 | -32.3 | 76 | 1031.2 |
| 12-12-2005 | 0 | 7 | -31.5 | -34.4 | 73 | 1028.3 |
| 12-13-2005 | 0 | 5 | -26.2 | -29.0 | 74 | 1016.8 |
| 12-14-2005 | 0 | 10 | -19.4 | -22.9 | 71 | 1009.3 |
| 12-15-2005 | 0 | 8 | -9.8 | -15.0 | 62 | 1007.4 |
| 12-16-2005 | 1 | 4 | -4.1 | -5.8 | 86 | 1011.7 |
| 12-17-2005 | 0 | 6 | -5.2 | -6.8 | 87 | 1014.2 |
| 12-18-2005 | 0 | 8 | -8.9 | -10.5 | 86 | 1017.0 |
| 12-19-2005 | 1 | 11 | -15.3 | -17.3 | 82 | 1017.8 |
| 12-20-2005 | 0 | 7 | -17.2 | -19.6 | 80 | 1016.5 |
| 12-21-2005 | 0 | 8 | -17.1 | -19.7 | 78 | 1010.1 |
| 12-22-2005 | 0 | 9 | -16.8 | -19.4 | 78 | 1006.7 |
| 12-23-2005 | 1 | 9 | -17.5 | -20.3 | 77 | 1004.7 |
| 12-24-2005 | 0 | 6 | -14.6 | -18.0 | 73 | 1004.3 |
| 12-25-2005 | 0 | 5 | -13.7 | -15.7 | 83 | 1007.0 |
| 12-26-2005 | 2 | 6 | -18.5 | -20.3 | 84 | 1007.3 |
| 12-27-2005 | 0 | 5 | -10.4 | -12.1 | 85 | 998.4 |
| 12-28-2005 | 0 | 2 | -5.0 | -8.0 | 77 | 995.8 |
| 12-29-2005 | 0 | 2 | -14.0 | -16.8 | 77 | 1002.5 |
| 12-30-2005 | 0 | 4 | -12.4 | -15.7 | 74 | 996.8 |
| 12-31-2005 | 0 | 6 | -12.6 | -15.7 | 74 | 1001.3 |
| 01-01-2006 | 0 | 4 | -28.0 | -31.6 | 69 | 1014.6 |
| 01-02-2006 | 0 | 12 | -33.4 | -36.9 | 68 | 1029.1 |
| 01-03-2006 | 0 | 8 | -30.2 | -33.9 | 67 | 1029.5 |
| 01-04-2006 | 0 | 7 | -17.7 | -23.5 | 57 | 1022.8 |
| 01-05-2006 | 0 | 10 | -13.6 | -18.4 | 64 | 1015.0 |
| 01-06-2006 | 0 | 6 | -12.8 | -15.6 | 77 | 1006.8 |
| 01-07-2006 | 0 | 8 | -22.6 | -25.2 | 77 | 1007.7 |
| 01-08-2006 | 0 | 7 | -21.3 | -23.9 | 77 | 1000.0 |
| 01-09-2006 | 0 | 11 | -17.1 | -20.1 | 75 | 988.7 |
| 01-10-2006 | 0 | 4 | -28.0 | -31.6 | 68 | 998.2 |
| 01-11-2006 | 0 | 8 | -31.8 | -35.5 | 67 | 1004.2 |
| 01-12-2006 | 0 | 8 | -27.3 | -30.4 | 72 | 1002.5 |
| 01-13-2006 | 0 | 8 | -29.7 | -34.2 | 62 | 1006.9 |
| 01-14-2006 | 1 | 5 | -14.7 | -17.7 | 76 | 997.2 |
| 01-15-2006 | 0 | 6 | -10.4 | -13.4 | 77 | 990.7 |
| 01-16-2006 | 0 | 9 | -7.9 | -10.1 | 82 | 996.1 |
| 01-17-2006 | 0 | 14 | -7.7 | -9.9 | 82 | 997.3 |
| 01-18-2006 | 1 | 3 | -6.6 | -9.1 | 81 | 998.8 |
| 01-19-2006 | 0 | 7 | -8.9 | -11.6 | 79 | 1000.0 |
| 01-20-2006 | 0 | 10 | -32.3 | -36.4 | 64 | 1016.2 |
| 01-21-2006 | 0 | 4 | -35.3 | -39.2 | 65 | 1014.0 |
| 01-22-2006 | 0 | 5 | -34.9 | -39.1 | 62 | 1011.9 |
| 01-23-2006 | 0 | 9 | -31.3 | -34.9 | 67 | 1004.8 |
| 01-24-2006 | 0 | 6 | -30.9 | -34.3 | 69 | 1006.0 |
| 01-25-2006 | 0 | 7 | -40.2 | -44.2 | 62 | 1011.2 |
| 01-26-2006 | 1 | 8 | -34.6 | -38.4 | 65 | 1002.6 |
| 01-27-2006 | 0 | 3 | -33.0 | -36.7 | 67 | 1009.4 |
| 01-28-2006 | 0 | 2 | -32.5 | -35.7 | 70 | 1011.6 |
| 01-29-2006 | 0 | 6 | -26.5 | -29.8 | 71 | 1012.0 |
| 01-30-2006 | 1 | 5 | -24.8 | -27.8 | 74 | 1016.3 |
| 01-31-2006 | 0 | 4 | -23.8 | -26.9 | 74 | 1013.3 |
| 02-01-2006 | 0 | 16 | -22.1 | -25.3 | 73 | 1011.5 |
| 02-02-2006 | 1 | 8 | -21.6 | -25.8 | 66 | 1009.0 |
| 02-03-2006 | 0 | 6 | -10.0 | -12.2 | 82 | 1005.9 |
| 02-04-2006 | 0 | 3 | -19.2 | -22.0 | 76 | 1015.2 |
| 02-05-2006 | 0 | 14 | -17.8 | -20.6 | 76 | 1010.5 |
| 02-06-2006 | 0 | 12 | -6.8 | -10.1 | 75 | 997.0 |
| 02-07-2006 | 1 | 7 | -6.7 | -9.0 | 81 | 1008.6 |
| 02-08-2006 | 0 | 11 | -7.1 | -9.0 | 84 | 1004.0 |
| 02-09-2006 | 0 | 10 | -2.2 | -4.1 | 85 | 995.7 |
| 02-10-2006 | 0 | 15 | -1.0 | -2.7 | 87 | 985.3 |
| 02-11-2006 | 0 | 9 | -13.4 | -17.4 | 69 | 988.7 |
| 02-12-2006 | 0 | 9 | -16.9 | -20.1 | 73 | 1006.3 |
| 02-13-2006 | 0 | 14 | -16.3 | -18.9 | 78 | 1015.6 |
| 02-14-2006 | 0 | 8 | -21.2 | -23.8 | 77 | 1022.7 |
| 02-15-2006 | 0 | 12 | -17.7 | -21.3 | 73 | 1017.5 |
| 02-16-2006 | 0 | 9 | -16.6 | -20.0 | 73 | 1008.1 |
| 02-17-2006 | 0 | 13 | -13.1 | -17.7 | 66 | 995.7 |
| 02-18-2006 | 1 | 8 | -9.7 | -13.7 | 70 | 990.2 |
| 02-19-2006 | 1 | 8 | -15.1 | -19.1 | 70 | 1004.7 |
| 02-20-2006 | 0 | 12 | -17.2 | -19.8 | 78 | 1014.5 |
| 02-21-2006 | 2 | 10 | -19.2 | -22.0 | 77 | 1013.9 |
| 02-22-2006 | 0 | 8 | -17.9 | -20.8 | 76 | 1012.8 |
| 02-23-2006 | 1 | 3 | -18.4 | -22.4 | 69 | 1013.4 |
| 02-24-2006 | 0 | 3 | -17.7 | -22.3 | 67 | 1007.6 |
| 02-25-2006 | 1 | 6 | -18.0 | -21.4 | 74 | 1006.3 |
| 02-26-2006 | 0 | 9 | -14.9 | -17.6 | 78 | 1009.0 |
| 02-27-2006 | 1 | 12 | -12.1 | -15.4 | 76 | 1009.8 |
| 02-28-2006 | 1 | 17 | -10.7 | -15.3 | 69 | 1006.2 |
| 03-01-2006 | 0 | 18 | -6.3 | -9.6 | 76 | 998.0 |
| 03-02-2006 | 0 | 11 | -5.5 | -8.5 | 77 | 996.1 |
| 03-03-2006 | 5 | 11 | -11.2 | -14.4 | 75 | 1003.1 |
| 03-04-2006 | 0 | 11 | -6.5 | -10.3 | 73 | 993.3 |
| 03-05-2006 | 0 | 3 | -10.0 | -13.1 | 76 | 999.7 |
| 03-06-2006 | 0 | 8 | -6.3 | -9.3 | 77 | 999.3 |
| 03-07-2006 | 1 | 11 | -2.9 | -6.1 | 76 | 995.8 |
| 03-08-2006 | 0 | 2 | -10.2 | -13.5 | 74 | 1007.0 |
| 03-09-2006 | 0 | 12 | -12.7 | -17.7 | 65 | 1018.3 |
| 03-10-2006 | 1 | 8 | -10.8 | -18.2 | 51 | 1017.3 |
| 03-11-2006 | 0 | 7 | -9.3 | -16.1 | 56 | 1011.4 |
| 03-12-2006 | 0 | 7 | -6.1 | -12.4 | 60 | 1009.6 |
| 03-13-2006 | 1 | 6 | -4.2 | -10.6 | 59 | 1006.7 |
| 03-14-2006 | 1 | 12 | -5.3 | -11.2 | 63 | 1003.2 |
| 03-15-2006 | 0 | 9 | -10.6 | -13.1 | 79 | 1001.7 |
| 03-16-2006 | 0 | 11 | -8.9 | -10.9 | 83 | 1003.0 |
| 03-17-2006 | 0 | 16 | -3.0 | -7.4 | 69 | 997.2 |
| 03-18-2006 | 0 | 10 | -3.5 | -7.5 | 72 | 993.8 |
| 03-19-2006 | 2 | 4 | -4.8 | -6.6 | 86 | 997.5 |
| 03-20-2006 | 2 | 6 | -6.6 | -8.5 | 85 | 1007.2 |
| 03-21-2006 | 0 | 6 | -7.0 | -12.0 | 69 | 1006.2 |
| 03-22-2006 | 0 | 8 | -3.7 | -10.3 | 61 | 999.1 |
| 03-23-2006 | 3 | 5 | -1.7 | -4.7 | 78 | 1000.8 |
| 03-24-2006 | 0 | 9 | -1.8 | -3.7 | 85 | 1006.9 |
| 03-25-2006 | 1 | 7 | -0.6 | -2.6 | 86 | 1006.2 |
| 03-26-2006 | 1 | 11 | 2.5 | -2.6 | 70 | 996.8 |
| 03-27-2006 | 1 | 12 | -0.9 | -5.8 | 67 | 999.8 |
| 03-28-2006 | 1 | 3 | 3.3 | -0.8 | 75 | 982.4 |
| 03-29-2006 | 1 | 7 | -2.2 | -6.6 | 69 | 996.3 |
| 03-30-2006 | 0 | 9 | -0.7 | -4.0 | 77 | 996.2 |
| 03-31-2006 | 0 | 14 | 2.2 | -0.4 | 84 | 989.2 |
| 04-01-2006 | 0 | 5 | 0.1 | -1.6 | 88 | 992.4 |
| 04-02-2006 | 0 | 7 | -4.6 | -12.0 | 53 | 1004.6 |
| 04-03-2006 | 0 | 11 | -0.9 | -8.6 | 56 | 998.6 |
| 04-04-2006 | 0 | 7 | 0.2 | -2.9 | 78 | 992.9 |
| 04-05-2006 | 0 | 13 | -5.8 | -13.3 | 52 | 1003.6 |
| 04-06-2006 | 0 | 13 | -6.3 | -15.4 | 45 | 1002.6 |
| 04-07-2006 | 1 | 8 | -4.9 | -14.5 | 43 | 994.7 |
| 04-08-2006 | 1 | 6 | -5.0 | -13.9 | 46 | 990.1 |
| 04-09-2006 | 0 | 8 | -6.5 | -13.9 | 53 | 1002.4 |
| 04-10-2006 | 0 | 10 | -5.8 | -11.4 | 61 | 1012.4 |
| 04-11-2006 | 0 | 11 | -3.9 | -8.7 | 65 | 1012.2 |
| 04-12-2006 | 1 | 6 | -4.3 | -13.2 | 47 | 1006.9 |
| 04-13-2006 | 0 | 21 | -1.6 | -10.4 | 52 | 1003.5 |
| 04-14-2006 | 0 | 11 | 3.8 | -0.2 | 74 | 991.4 |
| 04-15-2006 | 0 | 5 | 3.0 | -2.2 | 68 | 990.9 |
| 04-16-2006 | 0 | 9 | 1.1 | -6.3 | 58 | 999.6 |
| 04-17-2006 | 1 | 14 | 3.9 | -0.7 | 72 | 989.8 |
| 04-18-2006 | 2 | 13 | 11.2 | 2.8 | 56 | 981.7 |
| 04-19-2006 | 1 | 12 | 1.7 | -1.6 | 78 | 992.8 |
| 04-20-2006 | 3 | 13 | 1.4 | -6.6 | 55 | 1006.2 |
| 04-21-2006 | 0 | 6 | 4.4 | -6.3 | 48 | 998.0 |
| 04-22-2006 | 3 | 6 | 9.2 | 3.4 | 68 | 986.3 |
| 04-23-2006 | 0 | 11 | 4.4 | 3.0 | 90 | 991.3 |
| 04-24-2006 | 0 | 11 | 4.4 | 1.6 | 82 | 1001.1 |
| 04-25-2006 | 0 | 14 | 8.1 | 0.5 | 61 | 999.8 |
| 04-26-2006 | 1 | 12 | 9.3 | 2.6 | 64 | 993.5 |
| 04-27-2006 | 0 | 9 | 1.9 | 1.3 | 95 | 991.7 |
| 04-28-2006 | 0 | 9 | 2.5 | -4.3 | 65 | 986.5 |
| 04-29-2006 | 0 | 2 | 2.1 | -6.4 | 55 | 990.5 |
| 04-30-2006 | 0 | 3 | 2.8 | -1.0 | 78 | 990.7 |
| 05-01-2006 | 1 | 4 | 2.0 | 0.1 | 87 | 990.9 |
| 05-02-2006 | 1 | 4 | 2.2 | -6.6 | 53 | 996.6 |
| 05-03-2006 | 2 | 7 | 4.2 | -2.0 | 65 | 992.1 |
| 05-04-2006 | 1 | 17 | 2.8 | -4.6 | 58 | 999.8 |
| 05-05-2006 | 0 | 9 | 3.6 | -6.6 | 49 | 1003.4 |
| 05-06-2006 | 0 | 9 | 4.1 | -7.3 | 45 | 1001.1 |
| 05-07-2006 | 0 | 10 | 6.0 | -5.8 | 44 | 1004.2 |
| 05-08-2006 | 0 | 6 | 7.1 | -4.5 | 44 | 1005.7 |
| 05-09-2006 | 1 | 6 | 8.1 | -1.9 | 52 | 1005.7 |
| 05-10-2006 | 0 | 3 | 11.8 | -1.5 | 41 | 1006.4 |
| 05-11-2006 | 0 | 12 | 16.1 | -0.4 | 33 | 1006.2 |
| 05-12-2006 | 0 | 8 | 18.9 | 0.5 | 30 | 998.9 |
| 05-13-2006 | 1 | 4 | 20.4 | 2.8 | 33 | 986.8 |
| 05-14-2006 | 0 | 2 | 13.9 | 6.2 | 60 | 989.3 |
| 05-15-2006 | 0 | 6 | 11.4 | -2.0 | 41 | 998.4 |
| 05-16-2006 | 0 | 8 | 12.2 | 2.7 | 52 | 988.8 |
| 05-17-2006 | 0 | 14 | 14.5 | 0.8 | 44 | 984.6 |
| 05-18-2006 | 0 | 4 | 17.2 | 3.7 | 43 | 991.9 |
| 05-19-2006 | 0 | 6 | 16.3 | 3.0 | 41 | 995.7 |
| 05-20-2006 | 1 | 10 | 17.6 | 6.2 | 54 | 981.9 |
| 05-21-2006 | 0 | 8 | 5.8 | -4.8 | 50 | 998.6 |
| 05-22-2006 | 1 | 5 | 9.6 | -4.0 | 41 | 1003.0 |
| 05-23-2006 | 0 | 8 | 10.6 | -1.5 | 45 | 996.6 |
| 05-24-2006 | 0 | 8 | 17.2 | 3.1 | 40 | 993.3 |
| 05-25-2006 | 0 | 8 | 22.6 | 2.5 | 27 | 985.3 |
| 05-26-2006 | 1 | 4 | 20.1 | 8.5 | 49 | 983.3 |
| 05-27-2006 | 0 | 8 | 16.6 | 6.0 | 52 | 987.8 |
| 05-28-2006 | 1 | 4 | 14.0 | 4.0 | 51 | 994.6 |
| 05-29-2006 | 3 | 13 | 13.0 | 0.1 | 43 | 994.2 |
| 05-30-2006 | 0 | 6 | 9.2 | -2.2 | 46 | 998.3 |
| 05-31-2006 | 0 | 2 | 13.4 | -0.4 | 40 | 997.9 |
| 06-01-2006 | 0 | 4 | 16.9 | 2.1 | 39 | 992.6 |
| 06-02-2006 | 0 | 6 | 16.2 | 4.2 | 46 | 988.5 |
| 06-03-2006 | 0 | 7 | 17.4 | 6.8 | 51 | 986.0 |
| 06-04-2006 | 1 | 4 | 18.9 | 7.6 | 51 | 987.8 |
| 06-05-2006 | 1 | 5 | 20.0 | 9.6 | 53 | 991.2 |
| 06-06-2006 | 1 | 3 | 21.4 | 11.6 | 57 | 992.6 |
| 06-07-2006 | 1 | 10 | 22.5 | 11.4 | 53 | 994.0 |
| 06-08-2006 | 0 | 7 | 23.5 | 9.8 | 44 | 992.9 |
| 06-09-2006 | 0 | 5 | 24.0 | 14.3 | 57 | 989.3 |
| 06-10-2006 | 0 | 8 | 20.1 | 9.4 | 52 | 992.7 |
| 06-11-2006 | 1 | 11 | 18.4 | 7.1 | 50 | 993.5 |
| 06-12-2006 | 0 | 15 | 24.6 | 10.7 | 43 | 990.4 |
| 06-13-2006 | 0 | 5 | 24.2 | 15.4 | 59 | 984.2 |
| 06-14-2006 | 0 | 5 | 17.4 | 13.8 | 81 | 976.8 |
| 06-15-2006 | 0 | 9 | 10.9 | 8.0 | 83 | 989.3 |
| 06-16-2006 | 1 | 5 | 13.1 | 9.2 | 77 | 993.7 |
| 06-17-2006 | 0 | 6 | 17.3 | 11.5 | 70 | 996.7 |
| 06-18-2006 | 0 | 6 | 20.3 | 13.1 | 65 | 996.2 |
| 06-19-2006 | 0 | 11 | 21.5 | 12.6 | 59 | 994.5 |
| 06-20-2006 | 0 | 9 | 22.0 | 11.9 | 57 | 992.4 |
| 06-21-2006 | 0 | 9 | 22.6 | 13.1 | 58 | 988.6 |
| 06-22-2006 | 0 | 8 | 25.2 | 13.9 | 52 | 985.0 |
| 06-23-2006 | 0 | 6 | 26.7 | 14.8 | 49 | 981.9 |
| 06-24-2006 | 1 | 7 | 24.8 | 13.5 | 51 | 983.0 |
| 06-25-2006 | 0 | 1 | 23.1 | 13.2 | 55 | 984.8 |
| 06-26-2006 | 2 | 8 | 23.2 | 14.6 | 61 | 983.8 |
| 06-27-2006 | 0 | 4 | 22.9 | 15.6 | 64 | 981.2 |
| 06-28-2006 | 1 | 7 | 21.5 | 16.2 | 73 | 980.6 |
| 06-29-2006 | 0 | 10 | 22.8 | 15.6 | 64 | 980.2 |
| 06-30-2006 | 0 | 3 | 23.0 | 16.7 | 70 | 979.0 |
| 07-01-2006 | 0 | 10 | 22.5 | 17.1 | 73 | 979.6 |
| 07-02-2006 | 0 | 5 | 21.5 | 18.2 | 81 | 979.2 |
| 07-03-2006 | 1 | 7 | 23.5 | 18.2 | 74 | 980.2 |
| 07-04-2006 | 0 | 8 | 25.7 | 19.5 | 71 | 982.7 |
| 07-05-2006 | 0 | 4 | 21.4 | 15.0 | 68 | 987.1 |
| 07-06-2006 | 1 | 1 | 14.0 | 12.3 | 89 | 988.5 |
| 07-07-2006 | 1 | 2 | 17.7 | 10.6 | 65 | 990.8 |
| 07-08-2006 | 1 | 3 | 18.5 | 11.8 | 66 | 987.2 |
| 07-09-2006 | 0 | 3 | 12.8 | 10.4 | 85 | 989.8 |
| 07-10-2006 | 1 | 4 | 14.3 | 9.4 | 74 | 990.0 |
| 07-11-2006 | 1 | 8 | 15.7 | 10.6 | 73 | 989.0 |
| 07-12-2006 | 1 | 5 | 17.7 | 12.3 | 73 | 987.2 |
| 07-13-2006 | 1 | 4 | 19.6 | 11.9 | 65 | 988.2 |
| 07-14-2006 | 1 | 6 | 21.1 | 12.9 | 61 | 989.2 |
| 07-15-2006 | 0 | 4 | 17.8 | 14.6 | 82 | 987.7 |
| 07-16-2006 | 0 | 3 | 18.5 | 13.9 | 76 | 986.5 |
| 07-17-2006 | 0 | 8 | 19.5 | 14.0 | 72 | 987.8 |
| 07-18-2006 | 2 | 5 | 20.4 | 14.9 | 73 | 988.5 |
| 07-19-2006 | 1 | 3 | 19.4 | 16.9 | 86 | 989.8 |
| 07-20-2006 | 0 | 10 | 18.8 | 16.9 | 89 | 990.9 |
| 07-21-2006 | 1 | 10 | 20.5 | 17.2 | 82 | 989.1 |
| 07-22-2006 | 0 | 7 | 24.2 | 16.5 | 63 | 986.8 |
| 07-23-2006 | 0 | 3 | 20.4 | 16.0 | 77 | 982.5 |
| 07-24-2006 | 0 | 7 | 14.6 | 9.6 | 73 | 987.2 |
| 07-25-2006 | 1 | 6 | 16.9 | 8.0 | 59 | 985.5 |
| 07-26-2006 | 0 | 3 | 18.5 | 11.0 | 63 | 983.6 |
| 07-27-2006 | 1 | 5 | 18.1 | 12.1 | 71 | 981.0 |
| 07-28-2006 | 1 | 6 | 15.8 | 8.7 | 66 | 983.1 |
| 07-29-2006 | 0 | 3 | 19.6 | 11.9 | 63 | 981.2 |
| 07-30-2006 | 0 | 9 | 22.3 | 14.9 | 64 | 981.0 |
| 07-31-2006 | 0 | 8 | 19.3 | 11.6 | 63 | 983.9 |
| 08-01-2006 | 1 | 4 | 21.1 | 10.1 | 52 | 985.7 |
| 08-02-2006 | 0 | 7 | 19.0 | 10.1 | 58 | 986.8 |
| 08-03-2006 | 1 | 4 | 19.4 | 9.0 | 54 | 986.3 |
| 08-04-2006 | 0 | 6 | 19.2 | 13.4 | 70 | 982.9 |
| 08-05-2006 | 0 | 7 | 16.2 | 11.1 | 73 | 985.5 |
| 08-06-2006 | 0 | 3 | 16.6 | 9.2 | 64 | 985.7 |
| 08-07-2006 | 2 | 3 | 15.0 | 10.7 | 76 | 985.7 |
| 08-08-2006 | 0 | 2 | 13.7 | 7.9 | 71 | 986.1 |
| 08-09-2006 | 1 | 6 | 15.2 | 8.7 | 66 | 981.5 |
| 08-10-2006 | 0 | 6 | 14.7 | 9.6 | 72 | 989.8 |
| 08-11-2006 | 0 | 4 | 13.8 | 10.6 | 82 | 996.0 |
| 08-12-2006 | 1 | 0 | 15.5 | 9.4 | 69 | 993.6 |
| 08-13-2006 | 1 | 7 | 18.9 | 10.9 | 61 | 984.1 |
| 08-14-2006 | 0 | 6 | 19.8 | 10.9 | 59 | 977.6 |
| 08-15-2006 | 1 | 4 | 11.2 | 5.9 | 71 | 984.6 |
| 08-16-2006 | 1 | 6 | 12.7 | 6.3 | 65 | 986.1 |
| 08-17-2006 | 0 | 6 | 14.6 | 7.0 | 62 | 994.0 |
| 08-18-2006 | 0 | 6 | 13.9 | 10.6 | 81 | 996.5 |
| 08-19-2006 | 0 | 3 | 15.1 | 10.4 | 76 | 996.2 |
| 08-20-2006 | 0 | 6 | 15.3 | 9.0 | 68 | 993.8 |
| 08-21-2006 | 0 | 8 | 21.1 | 13.2 | 61 | 987.1 |
| 08-22-2006 | 0 | 7 | 19.8 | 13.8 | 70 | 983.0 |
| 08-23-2006 | 0 | 5 | 14.4 | 10.4 | 78 | 989.2 |
| 08-24-2006 | 0 | 7 | 13.5 | 7.7 | 70 | 992.3 |
| 08-25-2006 | 0 | 3 | 15.2 | 7.5 | 63 | 994.9 |
| 08-26-2006 | 0 | 5 | 9.7 | -0.4 | 54 | 1004.0 |
| 08-27-2006 | 0 | 5 | 10.8 | 2.1 | 60 | 996.5 |
| 08-28-2006 | 0 | 4 | 12.8 | 5.3 | 64 | 992.0 |
| 08-29-2006 | 1 | 6 | 15.4 | 7.3 | 63 | 987.5 |
| 08-30-2006 | 0 | 8 | 7.9 | 6.2 | 89 | 990.4 |
| 08-31-2006 | 0 | 0 | 8.2 | 3.8 | 75 | 997.3 |
| 09-01-2006 | 0 | 3 | 10.5 | 7.0 | 80 | 1004.6 |
| 09-02-2006 | 0 | 3 | 14.0 | 4.7 | 57 | 1004.9 |
| 09-03-2006 | 1 | 2 | 19.5 | 6.6 | 44 | 997.2 |
| 09-04-2006 | 0 | 4 | 14.1 | 8.6 | 70 | 998.5 |
| 09-05-2006 | 1 | 9 | 8.5 | 1.5 | 65 | 1002.7 |
| 09-06-2006 | 0 | 5 | 10.7 | 1.8 | 57 | 1003.9 |
| 09-07-2006 | 0 | 6 | 13.7 | 1.0 | 46 | 1003.8 |
| 09-08-2006 | 1 | 4 | 14.7 | 6.3 | 60 | 998.4 |
| 09-09-2006 | 0 | 4 | 21.3 | 6.0 | 43 | 994.9 |
| 09-10-2006 | 0 | 2 | 21.2 | 7.0 | 42 | 988.9 |
| 09-11-2006 | 1 | 5 | 16.8 | 10.7 | 69 | 989.4 |
| 09-12-2006 | 1 | 14 | 17.1 | 9.4 | 62 | 989.0 |
| 09-13-2006 | 0 | 6 | 17.8 | 11.3 | 68 | 984.1 |
| 09-14-2006 | 0 | 10 | 13.1 | 5.9 | 63 | 985.0 |
| 09-15-2006 | 0 | 12 | 10.0 | 5.1 | 72 | 989.9 |
| 09-16-2006 | 1 | 4 | 15.2 | 3.2 | 46 | 994.0 |
| 09-17-2006 | 2 | 7 | 17.2 | 3.6 | 42 | 988.3 |
| 09-18-2006 | 2 | 7 | 12.8 | 3.3 | 54 | 987.1 |
| 09-19-2006 | 1 | 17 | 7.2 | -3.0 | 51 | 997.3 |
| 09-20-2006 | 0 | 8 | 7.8 | -0.5 | 60 | 1001.5 |
| 09-21-2006 | 0 | 11 | 8.0 | 3.7 | 76 | 1003.8 |
| 09-22-2006 | 0 | 6 | 7.5 | -0.9 | 60 | 1002.8 |
| 09-23-2006 | 1 | 1 | 10.6 | 1.4 | 56 | 996.7 |
| 09-24-2006 | 0 | 3 | 13.7 | 0.5 | 47 | 984.0 |
| 09-25-2006 | 1 | 11 | 5.3 | 0.8 | 72 | 991.7 |
| 09-26-2006 | 0 | 6 | 12.1 | -2.1 | 39 | 992.3 |
| 09-27-2006 | 1 | 6 | 9.3 | 2.4 | 64 | 991.9 |
| 09-28-2006 | 0 | 7 | 11.8 | 3.2 | 60 | 985.9 |
| 09-29-2006 | 1 | 6 | 9.1 | -2.2 | 48 | 994.2 |
| 09-30-2006 | 0 | 12 | 14.2 | -1.5 | 34 | 993.2 |
| 10-01-2006 | 0 | 4 | 11.3 | 2.4 | 57 | 995.1 |
| 10-02-2006 | 1 | 9 | 11.4 | 2.7 | 59 | 993.7 |
| 10-03-2006 | 0 | 3 | 18.7 | 0.2 | 29 | 982.2 |
| 10-04-2006 | 0 | 4 | 4.5 | 2.5 | 86 | 986.6 |
| 10-05-2006 | 0 | 6 | -0.4 | -6.6 | 61 | 1006.3 |
| 10-06-2006 | 0 | 5 | 0.5 | -6.7 | 57 | 1001.4 |
| 10-07-2006 | 1 | 3 | 4.7 | 3.2 | 90 | 983.7 |
| 10-08-2006 | 1 | 10 | 2.2 | -0.3 | 83 | 995.7 |
| 10-09-2006 | 1 | 7 | 3.9 | 1.2 | 82 | 997.4 |
| 10-10-2006 | 2 | 4 | 6.8 | 2.4 | 74 | 987.7 |
| 10-11-2006 | 0 | 12 | 1.4 | -3.0 | 72 | 993.1 |
| 10-12-2006 | 0 | 0 | -0.7 | -7.3 | 58 | 1003.5 |
| 10-13-2006 | 0 | 9 | -1.9 | -6.8 | 66 | 997.6 |
| 10-14-2006 | 0 | 4 | 0.9 | -3.2 | 74 | 999.5 |
| 10-15-2006 | 0 | 5 | 2.7 | -2.7 | 69 | 1000.2 |
| 10-16-2006 | 0 | 5 | 4.2 | 0.0 | 75 | 993.9 |
| 10-17-2006 | 3 | 13 | 2.0 | -1.2 | 79 | 1000.2 |
| 10-18-2006 | 0 | 13 | 0.9 | -2.6 | 78 | 999.1 |
| 10-19-2006 | 1 | 5 | 0.1 | -4.3 | 72 | 1002.4 |
| 10-20-2006 | 1 | 6 | 2.8 | -3.3 | 65 | 995.7 |
| 10-21-2006 | 1 | 3 | 2.5 | -0.4 | 81 | 993.0 |
| 10-22-2006 | 1 | 2 | -3.5 | -9.3 | 62 | 1006.6 |
| 10-23-2006 | 0 | 4 | -6.8 | -11.7 | 68 | 1013.0 |
| 10-24-2006 | 0 | 8 | -2.3 | -8.0 | 63 | 1003.6 |
| 10-25-2006 | 0 | 6 | 3.9 | 0.0 | 76 | 1001.0 |
| 10-26-2006 | 1 | 3 | 6.8 | 3.9 | 82 | 1000.9 |
| 10-27-2006 | 0 | 10 | 7.9 | 3.8 | 75 | 1001.0 |
| 10-28-2006 | 0 | 9 | 8.1 | 2.4 | 67 | 995.2 |
| 10-29-2006 | 0 | 10 | 7.8 | 2.0 | 67 | 996.9 |
| 10-30-2006 | 0 | 6 | 9.4 | 1.8 | 61 | 996.9 |
| 10-31-2006 | 0 | 4 | 9.1 | 5.5 | 78 | 1001.3 |
| 11-01-2006 | 1 | 6 | 10.0 | 5.2 | 73 | 985.5 |
| 11-02-2006 | 0 | 3 | 0.8 | -4.9 | 65 | 998.2 |
| 11-03-2006 | 0 | 4 | 0.5 | -5.0 | 67 | 1007.0 |
| 11-04-2006 | 0 | 9 | 0.9 | -6.1 | 62 | 1005.8 |
| 11-05-2006 | 1 | 8 | 1.1 | -4.7 | 66 | 1002.7 |
| 11-06-2006 | 1 | 7 | 1.4 | -4.0 | 68 | 1000.0 |
| 11-07-2006 | 1 | 6 | 3.6 | -1.6 | 69 | 998.0 |
| 11-08-2006 | 0 | 1 | 6.7 | -0.1 | 63 | 994.5 |
| 11-09-2006 | 1 | 6 | 9.4 | 1.6 | 59 | 988.2 |
| 11-10-2006 | 0 | 7 | 3.4 | 1.1 | 84 | 999.9 |
| 11-11-2006 | 0 | 4 | 2.7 | -2.2 | 70 | 1009.3 |
| 11-12-2006 | 1 | 4 | 2.5 | -1.5 | 75 | 1008.1 |
| 11-13-2006 | 0 | 9 | -0.6 | -3.6 | 81 | 1001.1 |
| 11-14-2006 | 0 | 6 | 1.4 | -2.4 | 75 | 1001.8 |
| 11-15-2006 | 1 | 9 | -1.3 | -5.6 | 71 | 1004.7 |
| 11-16-2006 | 0 | 7 | -3.7 | -7.6 | 72 | 1001.4 |
| 11-17-2006 | 0 | 10 | -2.6 | -5.8 | 76 | 997.4 |
| 11-18-2006 | 0 | 5 | -2.1 | -3.6 | 88 | 995.2 |
| 11-19-2006 | 0 | 7 | -6.9 | -9.8 | 77 | 999.7 |
| 11-20-2006 | 1 | 8 | -2.9 | -4.9 | 85 | 995.6 |
| 11-21-2006 | 2 | 9 | -5.5 | -6.7 | 90 | 995.8 |
| 11-22-2006 | 0 | 7 | -8.8 | -10.4 | 86 | 1001.4 |
| 11-23-2006 | 0 | 3 | -14.2 | -16.7 | 78 | 1012.0 |
| 11-24-2006 | 0 | 3 | -20.9 | -24.3 | 72 | 1016.0 |
| 11-25-2006 | 0 | 5 | -13.9 | -16.1 | 81 | 1009.9 |
| 11-26-2006 | 0 | 0 | -11.1 | -13.4 | 81 | 1007.1 |
| 11-27-2006 | 1 | 9 | -12.9 | -15.2 | 81 | 1008.0 |
| 11-28-2006 | 0 | 15 | -16.2 | -18.7 | 79 | 1008.0 |
| 11-29-2006 | 0 | 8 | -16.4 | -19.2 | 76 | 1006.9 |
| 11-30-2006 | 0 | 4 | -11.0 | -14.8 | 71 | 1003.5 |
| 12-01-2006 | 0 | 4 | -10.8 | -12.9 | 82 | 1005.0 |
| 12-02-2006 | 0 | 9 | -16.0 | -19.4 | 72 | 1007.3 |
| 12-03-2006 | 0 | 8 | -12.8 | -16.8 | 69 | 1005.0 |
| 12-04-2006 | 0 | 8 | -9.9 | -13.9 | 69 | 1001.5 |
| 12-05-2006 | 0 | 9 | -5.7 | -9.5 | 72 | 1000.9 |
| 12-06-2006 | 0 | 4 | -2.6 | -5.1 | 81 | 1002.6 |
| 12-07-2006 | 0 | 13 | -2.6 | -5.3 | 79 | 1006.5 |
| 12-08-2006 | 0 | 12 | -2.4 | -4.9 | 80 | 1000.2 |
| 12-09-2006 | 0 | 3 | -2.1 | -4.3 | 83 | 995.0 |
| 12-10-2006 | 0 | 4 | -13.1 | -16.0 | 76 | 1005.2 |
| 12-11-2006 | 0 | 11 | -9.4 | -11.8 | 81 | 1002.2 |
| 12-12-2006 | 1 | 7 | -5.6 | -8.1 | 80 | 998.1 |
| 12-13-2006 | 0 | 7 | -4.2 | -5.8 | 86 | 1000.2 |
| 12-14-2006 | 0 | 11 | -1.8 | -2.9 | 90 | 1009.2 |
| 12-15-2006 | 0 | 5 | -3.4 | -5.3 | 84 | 1008.6 |
| 12-16-2006 | 0 | 6 | -7.5 | -11.0 | 74 | 1002.0 |
| 12-17-2006 | 0 | 1 | -1.8 | -3.9 | 83 | 995.4 |
| 12-18-2006 | 0 | 8 | -0.8 | -3.2 | 82 | 996.9 |
| 12-19-2006 | 0 | 16 | -1.4 | -4.5 | 77 | 994.2 |
| 12-20-2006 | 0 | 10 | -4.1 | -6.1 | 84 | 995.7 |
| 12-21-2006 | 0 | 7 | -8.2 | -10.6 | 82 | 999.7 |
| 12-22-2006 | 1 | 9 | -3.9 | -5.3 | 88 | 987.0 |
| 12-23-2006 | 0 | 5 | -13.6 | -16.6 | 76 | 997.2 |
| 12-24-2006 | 0 | 9 | -18.7 | -21.0 | 80 | 1001.2 |
| 12-25-2006 | 0 | 6 | -11.0 | -13.6 | 78 | 999.1 |
| 12-26-2006 | 0 | 11 | -6.0 | -7.2 | 90 | 1001.8 |
| 12-27-2006 | 0 | 7 | -5.4 | -7.9 | 80 | 1001.0 |
| 12-28-2006 | 0 | 6 | -4.8 | -8.2 | 74 | 992.7 |
| 12-29-2006 | 0 | 6 | -5.6 | -7.4 | 85 | 992.7 |
| 12-30-2006 | 0 | 4 | -9.1 | -12.0 | 77 | 995.4 |
| 12-31-2006 | 0 | 1 | -5.0 | -7.9 | 77 | 997.5 |
| 01-01-2007 | 0 | 5 | -10.9 | -14.5 | 72 | 1003.7 |
| 01-02-2007 | 0 | 8 | -17.0 | -19.9 | 76 | 1010.6 |
| 01-03-2007 | 0 | 7 | -14.4 | -16.8 | 80 | 1013.4 |
| 01-04-2007 | 0 | 7 | -8.2 | -10.6 | 81 | 1014.3 |
| 01-05-2007 | 0 | 6 | -6.3 | -10.4 | 69 | 1015.5 |
| 01-06-2007 | 0 | 12 | -8.0 | -13.9 | 60 | 1011.7 |
| 01-07-2007 | 0 | 5 | -10.4 | -14.4 | 70 | 998.9 |
| 01-08-2007 | 0 | 7 | -17.2 | -19.1 | 83 | 1003.6 |
| 01-09-2007 | 0 | 13 | -17.6 | -19.4 | 84 | 1005.3 |
| 01-10-2007 | 0 | 10 | -8.9 | -11.5 | 79 | 1008.0 |
| 01-11-2007 | 0 | 6 | -9.3 | -11.2 | 85 | 1009.3 |
| 01-12-2007 | 0 | 5 | -6.4 | -7.9 | 87 | 1006.1 |
| 01-13-2007 | 0 | 5 | -3.0 | -5.0 | 84 | 1004.3 |
| 01-14-2007 | 1 | 7 | -4.5 | -9.3 | 65 | 1005.4 |
| 01-15-2007 | 0 | 14 | -13.7 | -16.6 | 78 | 1003.6 |
| 01-16-2007 | 1 | 4 | -15.2 | -17.4 | 82 | 1001.7 |
| 01-17-2007 | 0 | 8 | -12.6 | -15.3 | 78 | 1003.1 |
| 01-18-2007 | 0 | 4 | -11.8 | -13.9 | 83 | 997.7 |
| 01-19-2007 | 0 | 8 | -5.6 | -9.2 | 73 | 999.1 |
| 01-20-2007 | 0 | 4 | -7.4 | -9.3 | 85 | 1006.3 |
| 01-21-2007 | 0 | 7 | -9.3 | -10.4 | 90 | 1010.3 |
| 01-22-2007 | 0 | 9 | -8.9 | -14.5 | 61 | 1007.3 |
| 01-23-2007 | 0 | 12 | -10.0 | -13.1 | 76 | 1003.3 |
| 01-24-2007 | 0 | 13 | -6.0 | -9.3 | 75 | 998.1 |
| 01-25-2007 | 0 | 12 | -6.8 | -9.1 | 82 | 1005.3 |
| 01-26-2007 | 0 | 9 | -2.8 | -5.2 | 82 | 996.8 |
| 01-27-2007 | 0 | 9 | -3.1 | -5.4 | 83 | 996.6 |
| 01-28-2007 | 0 | 7 | -6.1 | -8.2 | 83 | 999.6 |
| 01-29-2007 | 0 | 8 | -4.0 | -6.5 | 81 | 996.7 |
| 01-30-2007 | 0 | 10 | -1.0 | -5.6 | 70 | 999.9 |
| 01-31-2007 | 1 | 8 | 0.2 | -5.7 | 63 | 1000.1 |
| 02-01-2007 | 0 | 6 | -3.0 | -7.4 | 71 | 998.1 |
| 02-02-2007 | 0 | 8 | -3.8 | -10.7 | 57 | 995.3 |
| 02-03-2007 | 0 | 13 | -7.9 | -12.5 | 66 | 998.7 |
| 02-04-2007 | 0 | 10 | -6.8 | -8.6 | 84 | 996.6 |
| 02-05-2007 | 1 | 10 | -8.9 | -10.9 | 83 | 998.0 |
| 02-06-2007 | 0 | 27 | -6.9 | -11.3 | 68 | 992.8 |
| 02-07-2007 | 0 | 11 | -1.7 | -4.1 | 82 | 997.2 |
| 02-08-2007 | 0 | 7 | -5.8 | -8.3 | 80 | 1006.2 |
| 02-09-2007 | 0 | 10 | -7.7 | -9.0 | 89 | 1000.1 |
| 02-10-2007 | 0 | 13 | -8.4 | -10.7 | 81 | 1000.1 |
| 02-11-2007 | 0 | 9 | -14.7 | -16.6 | 83 | 1004.2 |
| 02-12-2007 | 0 | 9 | -6.8 | -8.9 | 83 | 998.8 |
| 02-13-2007 | 0 | 8 | -6.3 | -8.4 | 83 | 995.8 |
| 02-14-2007 | 1 | 9 | -18.0 | -21.5 | 71 | 1004.8 |
| 02-15-2007 | 0 | 13 | -21.2 | -24.5 | 72 | 1007.8 |
| 02-16-2007 | 0 | 6 | -13.7 | -16.8 | 75 | 996.5 |
| 02-17-2007 | 1 | 10 | -12.4 | -14.8 | 80 | 997.4 |
| 02-18-2007 | 0 | 8 | -13.3 | -16.3 | 76 | 993.6 |
| 02-19-2007 | 2 | 12 | -7.9 | -9.8 | 84 | 986.0 |
| 02-20-2007 | 0 | 7 | -13.7 | -17.1 | 72 | 999.3 |
| 02-21-2007 | 1 | 5 | -9.6 | -11.7 | 83 | 999.0 |
| 02-22-2007 | 0 | 4 | -7.8 | -10.0 | 82 | 995.0 |
| 02-23-2007 | 2 | 3 | -5.9 | -9.2 | 75 | 980.3 |
| 02-24-2007 | 1 | 5 | -15.0 | -18.8 | 70 | 988.8 |
| 02-25-2007 | 0 | 7 | -14.6 | -18.6 | 69 | 992.4 |
| 02-26-2007 | 0 | 8 | -11.6 | -15.1 | 72 | 979.0 |
| 02-27-2007 | 0 | 12 | -20.5 | -24.7 | 66 | 1007.5 |
| 02-28-2007 | 0 | 10 | -21.1 | -25.6 | 64 | 1015.1 |
| 03-01-2007 | 0 | 9 | -15.9 | -22.3 | 54 | 1015.6 |
| 03-02-2007 | 0 | 8 | -17.5 | -22.3 | 64 | 1022.7 |
| 03-03-2007 | 0 | 10 | -16.6 | -22.2 | 61 | 1019.1 |
| 03-04-2007 | 0 | 5 | -15.8 | -21.3 | 61 | 1015.8 |
| 03-05-2007 | 0 | 7 | -15.8 | -21.5 | 59 | 1013.7 |
| 03-06-2007 | 0 | 15 | -12.2 | -19.4 | 54 | 1010.8 |
| 03-07-2007 | 0 | 5 | -13.1 | -17.3 | 69 | 1012.2 |
| 03-08-2007 | 0 | 6 | -15.9 | -20.2 | 68 | 1015.0 |
| 03-09-2007 | 0 | 11 | -12.9 | -17.5 | 66 | 1005.7 |
| 03-10-2007 | 0 | 7 | -7.9 | -14.1 | 58 | 996.5 |
| 03-11-2007 | 0 | 14 | -11.2 | -14.1 | 77 | 1000.3 |
| 03-12-2007 | 1 | 8 | -14.4 | -18.0 | 72 | 1007.0 |
| 03-13-2007 | 0 | 5 | -15.3 | -20.7 | 62 | 1009.5 |
| 03-14-2007 | 0 | 11 | -14.8 | -20.9 | 58 | 1007.8 |
| 03-15-2007 | 0 | 9 | -10.0 | -16.4 | 57 | 1002.6 |
| 03-16-2007 | 0 | 8 | -9.8 | -14.7 | 67 | 1000.2 |
| 03-17-2007 | 0 | 9 | -6.7 | -14.2 | 56 | 998.7 |
| 03-18-2007 | 0 | 9 | -0.5 | -7.4 | 60 | 992.5 |
| 03-19-2007 | 2 | 9 | 0.2 | -3.3 | 78 | 993.5 |
| 03-20-2007 | 1 | 9 | -0.8 | -4.3 | 77 | 994.1 |
| 03-21-2007 | 2 | 10 | -3.3 | -6.9 | 74 | 1002.2 |
| 03-22-2007 | 0 | 8 | -1.9 | -5.2 | 75 | 1003.9 |
| 03-23-2007 | 1 | 10 | 1.0 | -1.3 | 84 | 996.8 |
| 03-24-2007 | 1 | 6 | 0.1 | -4.2 | 71 | 995.5 |
| 03-25-2007 | 2 | 4 | -0.5 | -8.1 | 54 | 995.6 |
| 03-26-2007 | 0 | 18 | 1.3 | -4.2 | 66 | 989.1 |
| 03-27-2007 | 0 | 9 | 2.9 | 1.2 | 88 | 981.9 |
| 03-28-2007 | 0 | 8 | -3.0 | -7.5 | 68 | 986.0 |
| 03-29-2007 | 0 | 9 | -2.5 | -6.5 | 71 | 990.0 |
| 03-30-2007 | 0 | 5 | -4.6 | -7.7 | 76 | 997.5 |
| 03-31-2007 | 0 | 8 | -4.4 | -9.8 | 63 | 1014.2 |
| 04-01-2007 | 0 | 9 | -1.2 | -10.2 | 52 | 1005.4 |
| 04-02-2007 | 0 | 13 | 1.8 | -2.5 | 73 | 991.8 |
| 04-03-2007 | 0 | 11 | 0.8 | -1.8 | 83 | 996.1 |
| 04-04-2007 | 1 | 11 | 0.9 | -2.8 | 77 | 1000.4 |
| 04-05-2007 | 0 | 15 | 3.0 | -3.7 | 64 | 1002.8 |
| 04-06-2007 | 1 | 10 | 8.3 | 0.2 | 58 | 996.2 |
| 04-07-2007 | 0 | 9 | 10.3 | 3.1 | 62 | 993.6 |
| 04-08-2007 | 0 | 10 | 5.8 | 2.2 | 79 | 996.8 |
| 04-09-2007 | 0 | 8 | 6.7 | -1.5 | 59 | 997.1 |
| 04-10-2007 | 1 | 21 | 9.9 | 1.3 | 58 | 996.0 |
| 04-11-2007 | 1 | 4 | 8.7 | 2.3 | 64 | 999.9 |
| 04-12-2007 | 1 | 12 | 3.4 | -4.5 | 58 | 1005.8 |
| 04-13-2007 | 0 | 14 | 4.6 | -6.2 | 48 | 1005.7 |
| 04-14-2007 | 0 | 14 | 7.3 | -4.7 | 43 | 994.9 |
| 04-15-2007 | 1 | 13 | 7.0 | 3.5 | 79 | 997.5 |
| 04-16-2007 | 1 | 8 | 11.5 | 3.0 | 59 | 992.2 |
| 04-17-2007 | 0 | 20 | 14.8 | 6.8 | 59 | 981.3 |
| 04-18-2007 | 0 | 12 | 5.2 | 2.8 | 84 | 991.7 |
| 04-19-2007 | 0 | 11 | 4.8 | 1.0 | 77 | 1000.6 |
| 04-20-2007 | 0 | 10 | 7.5 | -0.4 | 60 | 1006.0 |
| 04-21-2007 | 0 | 9 | 11.1 | -2.8 | 41 | 1007.2 |
| 04-22-2007 | 0 | 4 | 13.8 | -2.8 | 32 | 1003.3 |
| 04-23-2007 | 2 | 10 | 12.9 | -2.3 | 39 | 1001.5 |
| 04-24-2007 | 0 | 14 | 16.2 | -2.5 | 30 | 999.8 |
| 04-25-2007 | 0 | 10 | 16.4 | 0.8 | 37 | 999.9 |
| 04-26-2007 | 0 | 11 | 19.8 | 1.5 | 30 | 994.0 |
| 04-27-2007 | 0 | 7 | 16.6 | 5.4 | 54 | 986.2 |
| 04-28-2007 | 0 | 10 | 11.0 | 2.0 | 55 | 990.6 |
| 04-29-2007 | 0 | 4 | 14.6 | 1.3 | 41 | 992.0 |
| 04-30-2007 | 0 | 7 | 14.6 | 1.2 | 41 | 991.6 |
| 05-01-2007 | 0 | 3 | 16.1 | 1.2 | 39 | 996.1 |
| 05-02-2007 | 0 | 8 | 19.5 | 5.0 | 39 | 988.0 |
| 05-03-2007 | 1 | 9 | 16.1 | 1.7 | 38 | 992.9 |
| 05-04-2007 | 0 | 8 | 19.1 | 5.8 | 45 | 982.3 |
| 05-05-2007 | 1 | 8 | 7.3 | -3.5 | 50 | 998.0 |
| 05-06-2007 | 0 | 7 | 11.8 | -1.0 | 44 | 993.2 |
| 05-07-2007 | 1 | 10 | 13.9 | 0.7 | 41 | 988.3 |
| 05-08-2007 | 0 | 17 | 10.9 | 2.8 | 60 | 976.5 |
| 05-09-2007 | 3 | 6 | 7.5 | 2.1 | 70 | 993.2 |
| 05-10-2007 | 0 | 14 | 9.0 | 7.0 | 87 | 997.6 |
| 05-11-2007 | 0 | 12 | 15.9 | 4.3 | 47 | 990.3 |
| 05-12-2007 | 1 | 5 | 16.9 | 7.5 | 59 | 977.6 |
| 05-13-2007 | 1 | 6 | 10.9 | 5.1 | 68 | 988.5 |
| 05-14-2007 | 2 | 5 | 13.0 | 8.7 | 75 | 991.2 |
| 05-15-2007 | 3 | 2 | 17.7 | 13.1 | 75 | 989.0 |
| 05-16-2007 | 1 | 6 | 22.1 | 12.2 | 55 | 981.4 |
| 05-17-2007 | 0 | 7 | 13.8 | 5.7 | 61 | 990.0 |
| 05-18-2007 | 0 | 6 | 12.7 | 0.9 | 47 | 997.3 |
| 05-19-2007 | 0 | 8 | 17.2 | 9.8 | 64 | 987.8 |
| 05-20-2007 | 0 | 9 | 11.8 | 5.4 | 66 | 987.2 |
| 05-21-2007 | 3 | 10 | 7.2 | 1.3 | 68 | 991.8 |
| 05-22-2007 | 0 | 6 | 8.5 | 1.8 | 67 | 987.9 |
| 05-23-2007 | 0 | 4 | 13.4 | 7.5 | 69 | 985.4 |
| 05-24-2007 | 0 | 7 | 14.8 | 8.1 | 66 | 976.3 |
| 05-25-2007 | 0 | 10 | 10.8 | 3.6 | 62 | 989.7 |
| 05-26-2007 | 0 | 3 | 15.1 | 8.6 | 66 | 982.1 |
| 05-27-2007 | 1 | 2 | 12.7 | 6.2 | 68 | 985.3 |
| 05-28-2007 | 0 | 9 | 11.9 | 3.9 | 60 | 993.3 |
| 05-29-2007 | 1 | 4 | 12.5 | 8.4 | 76 | 993.4 |
| 05-30-2007 | 0 | 5 | 14.8 | 11.0 | 78 | 984.9 |
| 05-31-2007 | 1 | 5 | 8.2 | -1.0 | 53 | 993.2 |
| 06-01-2007 | 0 | 3 | 7.8 | -2.9 | 50 | 993.4 |
| 06-02-2007 | 0 | 5 | 10.9 | 5.7 | 71 | 984.6 |
| 06-03-2007 | 1 | 9 | 9.7 | 6.5 | 81 | 983.7 |
| 06-04-2007 | 1 | 8 | 9.0 | 8.3 | 95 | 973.3 |
| 06-05-2007 | 1 | 5 | 8.4 | 3.0 | 69 | 984.2 |
| 06-06-2007 | 0 | 4 | 9.6 | 1.6 | 60 | 996.1 |
| 06-07-2007 | 2 | 13 | 14.7 | 4.4 | 53 | 990.2 |
| 06-08-2007 | 1 | 3 | 12.0 | 4.8 | 61 | 993.8 |
| 06-09-2007 | 0 | 8 | 13.9 | 5.5 | 59 | 995.8 |
| 06-10-2007 | 0 | 4 | 16.6 | 8.8 | 62 | 985.8 |
| 06-11-2007 | 0 | 5 | 17.8 | 12.1 | 71 | 978.8 |
| 06-12-2007 | 0 | 14 | 15.7 | 7.5 | 60 | 982.4 |
| 06-13-2007 | 0 | 5 | 14.4 | 6.7 | 61 | 986.9 |
| 06-14-2007 | 0 | 0 | 13.1 | 6.7 | 69 | 989.3 |
| 06-15-2007 | 0 | 4 | 11.9 | 6.8 | 73 | 990.0 |
| 06-16-2007 | 1 | 4 | 14.8 | 6.8 | 61 | 989.8 |
| 06-17-2007 | 0 | 6 | 22.7 | 12.6 | 54 | 983.2 |
| 06-18-2007 | 1 | 4 | 16.8 | 8.4 | 59 | 986.8 |
| 06-19-2007 | 1 | 9 | 19.2 | 7.3 | 46 | 988.5 |
| 06-20-2007 | 1 | 4 | 20.1 | 14.6 | 72 | 985.8 |
| 06-21-2007 | 0 | 5 | 22.2 | 14.2 | 63 | 986.9 |
| 06-22-2007 | 2 | 12 | 23.1 | 16.0 | 66 | 987.0 |
| 06-23-2007 | 1 | 7 | 23.5 | 17.6 | 70 | 985.2 |
| 06-24-2007 | 1 | 5 | 23.4 | 16.1 | 64 | 980.4 |
| 06-25-2007 | 0 | 11 | 16.2 | 8.0 | 59 | 985.1 |
| 06-26-2007 | 0 | 10 | 16.6 | 7.9 | 59 | 984.6 |
| 06-27-2007 | 1 | 9 | 19.3 | 11.5 | 63 | 983.1 |
| 06-28-2007 | 1 | 2 | 21.4 | 14.7 | 67 | 978.3 |
| 06-29-2007 | 0 | 8 | 18.6 | 14.5 | 78 | 974.3 |
| 06-30-2007 | 1 | 5 | 18.1 | 15.1 | 83 | 976.0 |
| 07-01-2007 | 0 | 8 | 18.7 | 15.6 | 82 | 979.6 |
| 07-02-2007 | 0 | 5 | 20.0 | 15.1 | 74 | 981.2 |
| 07-03-2007 | 3 | 6 | 21.4 | 15.5 | 70 | 981.3 |
| 07-04-2007 | 0 | 2 | 21.0 | 14.5 | 67 | 982.0 |
| 07-05-2007 | 0 | 5 | 21.4 | 15.8 | 72 | 980.7 |
| 07-06-2007 | 1 | 4 | 21.9 | 16.6 | 74 | 979.9 |
| 07-07-2007 | 1 | 8 | 24.7 | 17.6 | 67 | 982.8 |
| 07-08-2007 | 0 | 5 | 24.0 | 19.6 | 77 | 983.0 |
| 07-09-2007 | 1 | 8 | 24.6 | 17.0 | 65 | 981.1 |
| 07-10-2007 | 1 | 6 | 21.3 | 17.8 | 81 | 980.8 |
| 07-11-2007 | 0 | 10 | 21.5 | 18.5 | 84 | 981.0 |
| 07-12-2007 | 0 | 8 | 23.6 | 18.2 | 74 | 982.5 |
| 07-13-2007 | 1 | 3 | 21.4 | 19.0 | 86 | 984.8 |
| 07-14-2007 | 0 | 2 | 23.0 | 16.3 | 68 | 983.6 |
| 07-15-2007 | 0 | 7 | 21.7 | 13.3 | 61 | 983.7 |
| 07-16-2007 | 0 | 8 | 22.3 | 13.5 | 59 | 986.1 |
| 07-17-2007 | 0 | 13 | 21.3 | 12.2 | 58 | 987.2 |
| 07-18-2007 | 1 | 3 | 22.2 | 15.2 | 67 | 987.4 |
| 07-19-2007 | 0 | 10 | 23.4 | 15.2 | 63 | 986.9 |
| 07-20-2007 | 0 | 6 | 25.4 | 16.4 | 61 | 982.2 |
| 07-21-2007 | 1 | 7 | 22.2 | 17.3 | 74 | 979.8 |
| 07-22-2007 | 1 | 8 | 20.8 | 15.3 | 72 | 979.6 |
| 07-23-2007 | 0 | 2 | 22.4 | 11.3 | 53 | 980.7 |
| 07-24-2007 | 0 | 9 | 23.8 | 11.8 | 49 | 981.9 |
| 07-25-2007 | 0 | 7 | 21.1 | 13.1 | 63 | 981.3 |
| 07-26-2007 | 0 | 6 | 18.7 | 13.7 | 75 | 986.5 |
| 07-27-2007 | 1 | 4 | 18.4 | 13.9 | 76 | 989.4 |
| 07-28-2007 | 1 | 6 | 18.1 | 15.1 | 83 | 991.2 |
| 07-29-2007 | 0 | 2 | 18.9 | 15.2 | 80 | 989.9 |
| 07-30-2007 | 0 | 11 | 19.4 | 13.5 | 71 | 990.7 |
| 07-31-2007 | 3 | 9 | 20.3 | 10.8 | 58 | 990.7 |
| 08-01-2007 | 0 | 7 | 21.2 | 10.8 | 56 | 987.8 |
| 08-02-2007 | 0 | 8 | 22.0 | 13.2 | 62 | 986.9 |
| 08-03-2007 | 0 | 11 | 22.9 | 15.6 | 66 | 987.0 |
| 08-04-2007 | 0 | 5 | 21.0 | 13.3 | 64 | 986.4 |
| 08-05-2007 | 1 | 6 | 18.6 | 11.9 | 67 | 986.9 |
| 08-06-2007 | 0 | 7 | 17.2 | 10.6 | 67 | 989.0 |
| 08-07-2007 | 0 | 10 | 21.3 | 10.5 | 54 | 984.9 |
| 08-08-2007 | 0 | 14 | 21.5 | 16.0 | 71 | 982.2 |
| 08-09-2007 | 0 | 4 | 21.1 | 14.3 | 68 | 979.0 |
| 08-10-2007 | 0 | 9 | 15.9 | 9.6 | 69 | 983.2 |
| 08-11-2007 | 0 | 11 | 13.1 | 10.5 | 85 | 982.2 |
| 08-12-2007 | 0 | 4 | 16.1 | 7.6 | 61 | 983.7 |
| 08-13-2007 | 0 | 6 | 14.2 | 5.8 | 58 | 984.0 |
| 08-14-2007 | 0 | 8 | 13.5 | 4.3 | 56 | 985.8 |
| 08-15-2007 | 0 | 4 | 11.0 | 5.7 | 72 | 989.6 |
| 08-16-2007 | 0 | 7 | 10.5 | 6.6 | 78 | 994.4 |
| 08-17-2007 | 0 | 3 | 10.2 | 4.3 | 70 | 996.4 |
| 08-18-2007 | 0 | 4 | 11.8 | 5.9 | 71 | 995.8 |
| 08-19-2007 | 0 | 4 | 12.0 | 8.8 | 82 | 996.4 |
| 08-20-2007 | 0 | 11 | 14.7 | 7.8 | 68 | 996.4 |
| 08-21-2007 | 0 | 6 | 17.0 | 8.4 | 59 | 995.2 |
| 08-22-2007 | 0 | 9 | 16.9 | 12.0 | 74 | 993.8 |
| 08-23-2007 | 0 | 5 | 16.1 | 8.2 | 64 | 994.8 |
| 08-24-2007 | 0 | 8 | 18.0 | 7.0 | 54 | 990.5 |
| 08-25-2007 | 0 | 2 | 10.6 | 3.7 | 66 | 999.8 |
| 08-26-2007 | 1 | 2 | 12.4 | 3.5 | 58 | 997.4 |
| 08-27-2007 | 0 | 4 | 13.6 | 9.1 | 75 | 993.9 |
| 08-28-2007 | 0 | 6 | 20.2 | 12.0 | 63 | 991.5 |
| 08-29-2007 | 0 | 8 | 20.7 | 12.5 | 63 | 993.8 |
| 08-30-2007 | 0 | 8 | 21.5 | 13.3 | 62 | 993.3 |
| 08-31-2007 | 0 | 10 | 21.1 | 13.0 | 64 | 988.9 |
| 09-01-2007 | 0 | 4 | 22.5 | 14.9 | 66 | 986.6 |
| 09-02-2007 | 1 | 5 | 24.0 | 15.1 | 61 | 987.8 |
| 09-03-2007 | 0 | 8 | 23.9 | 14.2 | 57 | 984.1 |
| 09-04-2007 | 0 | 11 | 17.6 | 10.9 | 66 | 985.3 |
| 09-05-2007 | 0 | 5 | 11.6 | 6.8 | 75 | 995.0 |
| 09-06-2007 | 0 | 6 | 13.7 | 7.7 | 70 | 993.9 |
| 09-07-2007 | 0 | 3 | 15.8 | 7.2 | 61 | 987.9 |
| 09-08-2007 | 0 | 5 | 10.3 | 4.7 | 72 | 994.4 |
| 09-09-2007 | 0 | 4 | 10.2 | 3.2 | 65 | 998.0 |
| 09-10-2007 | 0 | 9 | 16.3 | 2.4 | 43 | 995.8 |
| 09-11-2007 | 0 | 11 | 19.6 | 6.4 | 46 | 991.5 |
| 09-12-2007 | 0 | 5 | 22.8 | 6.7 | 39 | 980.6 |
| 09-13-2007 | 0 | 2 | 16.4 | 7.8 | 57 | 982.8 |
| 09-14-2007 | 0 | 9 | 10.7 | 6.2 | 74 | 990.7 |
| 09-15-2007 | 0 | 6 | 7.9 | 1.9 | 68 | 1000.8 |
| 09-16-2007 | 1 | 3 | 8.6 | 1.8 | 66 | 996.3 |
| 09-17-2007 | 0 | 7 | 11.6 | 1.6 | 55 | 990.5 |
| 09-18-2007 | 1 | 6 | 13.1 | 4.2 | 57 | 994.6 |
| 09-19-2007 | 0 | 8 | 12.4 | 4.6 | 62 | 992.1 |
| 09-20-2007 | 0 | 3 | 13.8 | 4.5 | 58 | 984.8 |
| 09-21-2007 | 0 | 5 | 7.9 | 6.5 | 90 | 991.2 |
| 09-22-2007 | 0 | 7 | 6.3 | 5.1 | 92 | 997.3 |
| 09-23-2007 | 0 | 6 | 5.4 | 4.0 | 90 | 1002.4 |
| 09-24-2007 | 0 | 5 | 6.2 | -0.2 | 67 | 1004.4 |
| 09-25-2007 | 0 | 7 | 8.7 | -0.6 | 56 | 1001.1 |
| 09-26-2007 | 0 | 2 | 10.8 | 0.5 | 53 | 997.7 |
| 09-27-2007 | 0 | 6 | 6.2 | 1.2 | 72 | 1002.6 |
| 09-28-2007 | 1 | 5 | 7.6 | -0.6 | 59 | 1006.4 |
| 09-29-2007 | 0 | 2 | 10.9 | 0.0 | 48 | 998.7 |
| 09-30-2007 | 0 | 4 | 12.7 | 3.9 | 57 | 990.2 |
| 10-01-2007 | 2 | 3 | 13.0 | 4.9 | 59 | 984.8 |
| 10-02-2007 | 1 | 10 | 12.3 | 2.6 | 53 | 982.7 |
| 10-03-2007 | 0 | 2 | 1.1 | -4.0 | 69 | 1001.1 |
| 10-04-2007 | 1 | 10 | 2.0 | -0.3 | 84 | 1010.0 |
| 10-05-2007 | 1 | 5 | 4.4 | 1.7 | 83 | 1012.1 |
| 10-06-2007 | 1 | 1 | 6.1 | 0.1 | 66 | 1008.1 |
| 10-07-2007 | 0 | 4 | 7.7 | 2.0 | 67 | 1002.5 |
| 10-08-2007 | 0 | 6 | 2.7 | -0.1 | 82 | 1001.3 |
| 10-09-2007 | 1 | 2 | -0.9 | -7.8 | 58 | 1014.4 |
| 10-10-2007 | 0 | 1 | -0.2 | -8.1 | 55 | 1014.1 |
| 10-11-2007 | 0 | 4 | -0.3 | -7.1 | 62 | 1009.4 |
| 10-12-2007 | 1 | 5 | 5.5 | -6.6 | 46 | 1004.6 |
| 10-13-2007 | 0 | 7 | 11.1 | -4.2 | 37 | 999.5 |
| 10-14-2007 | 0 | 7 | 7.0 | 0.7 | 65 | 1002.3 |
| 10-15-2007 | 0 | 5 | 4.9 | -1.2 | 66 | 1005.8 |
| 10-16-2007 | 0 | 7 | 0.7 | -5.2 | 67 | 1010.5 |
| 10-17-2007 | 0 | 3 | 4.2 | -3.1 | 62 | 1002.4 |
| 10-18-2007 | 0 | 10 | 12.3 | -0.2 | 45 | 993.4 |
| 10-19-2007 | 0 | 2 | 7.4 | 4.0 | 79 | 997.4 |
| 10-20-2007 | 0 | 2 | 5.1 | 0.6 | 73 | 1000.0 |
| 10-21-2007 | 0 | 4 | 6.8 | 3.0 | 77 | 998.5 |
| 10-22-2007 | 0 | 4 | 0.8 | -3.8 | 70 | 1003.0 |
| 10-23-2007 | 0 | 9 | -1.5 | -6.2 | 68 | 999.8 |
| 10-24-2007 | 0 | 5 | -0.3 | -1.7 | 90 | 996.7 |
| 10-25-2007 | 0 | 4 | -1.3 | -3.7 | 82 | 1008.1 |
| 10-26-2007 | 0 | 6 | -2.2 | -6.4 | 70 | 1011.0 |
| 10-27-2007 | 0 | 3 | -0.0 | -1.5 | 89 | 997.6 |
| 10-28-2007 | 0 | 6 | -1.7 | -6.0 | 65 | 999.7 |
| 10-29-2007 | 0 | 3 | -1.5 | -4.6 | 78 | 1004.3 |
| 10-30-2007 | 0 | 6 | 1.4 | 0.2 | 91 | 1007.9 |
| 10-31-2007 | 0 | 3 | 1.8 | -0.2 | 86 | 1012.1 |
| 11-01-2007 | 1 | 4 | 0.6 | -1.0 | 88 | 1007.9 |
| 11-02-2007 | 1 | 4 | -0.4 | -2.1 | 87 | 1000.9 |
| 11-03-2007 | 0 | 7 | -1.0 | -2.6 | 87 | 998.0 |
| 11-04-2007 | 0 | 1 | -0.6 | -2.8 | 85 | 994.5 |
| 11-05-2007 | 2 | 6 | 5.0 | -3.3 | 57 | 990.5 |
| 11-06-2007 | 0 | 6 | 0.8 | -3.9 | 70 | 1002.9 |
| 11-07-2007 | 0 | 7 | 3.1 | -3.8 | 60 | 991.7 |
| 11-08-2007 | 0 | 3 | 4.3 | 2.0 | 86 | 985.1 |
| 11-09-2007 | 0 | 4 | -7.1 | -9.9 | 78 | 1001.4 |
| 11-10-2007 | 0 | 2 | -13.1 | -17.0 | 70 | 1016.3 |
| 11-11-2007 | 0 | 5 | -12.9 | -16.3 | 73 | 1011.5 |
| 11-12-2007 | 0 | 4 | -10.1 | -14.6 | 69 | 1003.9 |
| 11-13-2007 | 0 | 6 | -12.7 | -15.6 | 78 | 1004.8 |
| 11-14-2007 | 0 | 5 | -10.8 | -13.6 | 77 | 1004.6 |
| 11-15-2007 | 0 | 6 | -6.0 | -8.6 | 79 | 1007.5 |
| 11-16-2007 | 0 | 4 | -4.9 | -7.3 | 81 | 1012.9 |
| 11-17-2007 | 0 | 6 | -1.0 | -2.9 | 86 | 1006.8 |
| 11-18-2007 | 0 | 3 | -0.7 | -4.0 | 77 | 1005.0 |
| 11-19-2007 | 0 | 8 | -0.9 | -5.3 | 71 | 997.2 |
| 11-20-2007 | 0 | 3 | -2.4 | -8.2 | 62 | 991.8 |
| 11-21-2007 | 0 | 4 | -5.2 | -12.4 | 53 | 998.6 |
| 11-22-2007 | 0 | 1 | -1.5 | -4.5 | 78 | 980.4 |
| 11-23-2007 | 0 | 7 | -14.9 | -19.0 | 68 | 1000.5 |
| 11-24-2007 | 0 | 6 | -9.6 | -14.4 | 65 | 1007.3 |
| 11-25-2007 | 0 | 3 | -5.8 | -13.4 | 51 | 1009.0 |
| 11-26-2007 | 0 | 4 | -8.7 | -13.2 | 67 | 1008.3 |
| 11-27-2007 | 0 | 6 | -11.1 | -13.2 | 83 | 1007.7 |
| 11-28-2007 | 0 | 6 | -15.4 | -17.0 | 86 | 1004.1 |
| 11-29-2007 | 0 | 2 | -10.5 | -12.1 | 86 | 1004.5 |
| 11-30-2007 | 0 | 8 | -6.9 | -7.5 | 94 | 1004.8 |
| 12-01-2007 | 0 | 5 | -5.6 | -7.4 | 86 | 1006.6 |
| 12-02-2007 | 0 | 9 | -9.2 | -10.1 | 93 | 1013.2 |
| 12-03-2007 | 0 | 17 | -7.2 | -8.0 | 93 | 1007.1 |
| 12-04-2007 | 1 | 3 | -6.5 | -7.6 | 91 | 1005.6 |
| 12-05-2007 | 0 | 12 | -6.4 | -7.7 | 89 | 1009.4 |
| 12-06-2007 | 0 | 1 | -6.3 | -7.7 | 89 | 1011.5 |
| 12-07-2007 | 0 | 3 | -10.1 | -11.1 | 91 | 1009.0 |
| 12-08-2007 | 0 | 2 | -15.7 | -17.1 | 88 | 1010.8 |
| 12-09-2007 | 0 | 1 | -17.1 | -18.8 | 84 | 1007.3 |
| 12-10-2007 | 0 | 6 | -14.6 | -16.1 | 86 | 1009.0 |
| 12-11-2007 | 0 | 3 | -15.1 | -17.0 | 83 | 1009.5 |
| 12-12-2007 | 0 | 7 | -11.9 | -14.1 | 81 | 1006.2 |
| 12-13-2007 | 0 | 5 | -9.4 | -11.7 | 82 | 1004.7 |
| 12-14-2007 | 0 | 5 | -11.4 | -10.2 | 85 | 1004.2 |
| 12-15-2007 | 0 | 1 | -12.0 | -15.3 | 75 | 1004.5 |
| 12-16-2007 | 0 | 5 | -9.7 | -14.0 | 68 | 1001.6 |
| 12-17-2007 | 0 | 2 | -9.8 | -14.4 | 65 | 995.0 |
| 12-18-2007 | 0 | 3 | -10.8 | -14.1 | 74 | 990.0 |
| 12-19-2007 | 0 | 4 | -6.8 | -11.2 | 67 | 988.0 |
| 12-20-2007 | 0 | 4 | -4.9 | -9.4 | 67 | 988.1 |
| 12-21-2007 | 0 | 4 | -6.1 | -8.1 | 84 | 992.5 |
| 12-22-2007 | 0 | 5 | -2.7 | -5.7 | 78 | 988.5 |
| 12-23-2007 | 0 | 4 | -5.0 | -9.0 | 70 | 988.3 |
| 12-24-2007 | 0 | 12 | -3.8 | -6.7 | 78 | 986.8 |
| 12-25-2007 | 0 | 2 | -16.7 | -20.9 | 67 | 1002.0 |
| 12-26-2007 | 0 | 3 | -22.9 | -26.1 | 72 | 1013.8 |
| 12-27-2007 | 0 | 4 | -16.3 | -18.5 | 81 | 1013.3 |
| 12-28-2007 | 0 | 9 | -13.0 | -15.7 | 77 | 1016.0 |
| 12-29-2007 | 0 | 2 | -13.6 | -15.7 | 81 | 1016.2 |
| 12-30-2007 | 0 | 4 | -16.4 | -20.3 | 69 | 1018.2 |
| 12-31-2007 | 0 | 6 | -17.0 | -20.4 | 73 | 1016.8 |
| 01-01-2008 | 0 | 5 | -11.2 | -15.7 | 66 | 1010.2 |
| 01-02-2008 | 0 | 9 | -14.1 | -20.1 | 57 | 1002.4 |
| 01-03-2008 | 0 | 8 | -22.5 | -24.6 | 81 | 1004.2 |
| 01-04-2008 | 0 | 8 | -32.8 | -35.3 | 76 | 1015.4 |
| 01-05-2008 | 1 | 4 | -26.9 | -30.0 | 73 | 1012.7 |
| 01-06-2008 | 0 | 9 | -17.1 | -19.5 | 79 | 1008.7 |
| 01-07-2008 | 0 | 9 | -17.8 | -19.6 | 84 | 1011.0 |
| 01-08-2008 | 0 | 4 | -22.9 | -24.6 | 84 | 1010.8 |
| 01-09-2008 | 0 | 4 | -17.8 | -19.3 | 86 | 1012.3 |
| 01-10-2008 | 0 | 7 | -17.2 | -19.0 | 84 | 1013.1 |
| 01-11-2008 | 0 | 6 | -26.0 | -27.7 | 85 | 1017.3 |
| 01-12-2008 | 1 | 10 | -27.4 | -29.3 | 82 | 1017.3 |
| 01-13-2008 | 3 | 2 | -26.2 | -28.1 | 82 | 1013.5 |
| 01-14-2008 | 1 | 5 | -21.4 | -23.1 | 84 | 1013.9 |
| 01-15-2008 | 0 | 7 | -16.9 | -18.0 | 89 | 1009.7 |
| 01-16-2008 | 1 | 4 | -20.9 | -22.5 | 86 | 1009.1 |
| 01-17-2008 | 0 | 10 | -30.8 | -33.0 | 78 | 1019.6 |
| 01-18-2008 | 0 | 6 | -30.3 | -32.7 | 77 | 1026.7 |
| 01-19-2008 | 0 | 3 | -22.7 | -25.2 | 77 | 1020.1 |
| 01-20-2008 | 0 | 5 | -15.5 | -17.2 | 85 | 1014.1 |
| 01-21-2008 | 0 | 3 | -13.8 | -15.5 | 85 | 1011.2 |
| 01-22-2008 | 0 | 4 | -13.4 | -15.6 | 81 | 1013.8 |
| 01-23-2008 | 0 | 4 | -13.6 | -15.8 | 81 | 1018.6 |
| 01-24-2008 | 1 | 6 | -15.9 | -18.1 | 81 | 1018.2 |
| 01-25-2008 | 0 | 4 | -18.0 | -20.7 | 78 | 1018.7 |
| 01-26-2008 | 0 | 6 | -24.2 | -26.3 | 81 | 1019.9 |
| 01-27-2008 | 0 | 6 | -22.7 | -25.0 | 79 | 1013.2 |
| 01-28-2008 | 0 | 11 | -22.9 | -25.6 | 76 | 1007.7 |
| 01-29-2008 | 0 | 6 | -24.8 | -27.2 | 78 | 1008.8 |
| 01-30-2008 | 1 | 5 | -23.9 | -26.2 | 79 | 1010.2 |
| 01-31-2008 | 1 | 9 | -20.7 | -23.6 | 75 | 1007.8 |
| 02-01-2008 | 1 | 13 | -16.6 | -18.5 | 83 | 1003.9 |
| 02-02-2008 | 0 | 6 | -20.6 | -22.4 | 84 | 1008.6 |
| 02-03-2008 | 1 | 1 | -18.6 | -20.5 | 83 | 1013.5 |
| 02-04-2008 | 0 | 11 | -20.1 | -22.0 | 83 | 1016.1 |
| 02-05-2008 | 0 | 12 | -20.4 | -22.7 | 80 | 1009.6 |
| 02-06-2008 | 0 | 12 | -13.9 | -17.0 | 75 | 1009.5 |
| 02-07-2008 | 0 | 7 | -10.9 | -13.2 | 81 | 1010.4 |
| 02-08-2008 | 0 | 12 | -14.8 | -16.9 | 82 | 1013.2 |
| 02-09-2008 | 0 | 9 | -18.4 | -20.5 | 81 | 1019.3 |
| 02-10-2008 | 0 | 4 | -19.7 | -22.3 | 77 | 1019.1 |
| 02-11-2008 | 0 | 10 | -14.6 | -18.4 | 70 | 1013.0 |
| 02-12-2008 | 1 | 9 | -14.1 | -17.2 | 75 | 1012.0 |
| 02-13-2008 | 0 | 11 | -18.9 | -22.0 | 75 | 1007.2 |
| 02-14-2008 | 0 | 9 | -20.4 | -23.4 | 75 | 1003.8 |
| 02-15-2008 | 0 | 12 | -18.5 | -21.0 | 79 | 1002.2 |
| 02-16-2008 | 1 | 7 | -13.5 | -15.9 | 80 | 1005.9 |
| 02-17-2008 | 0 | 10 | -12.7 | -16.2 | 73 | 1009.5 |
| 02-18-2008 | 0 | 11 | -9.5 | -12.2 | 78 | 1001.5 |
| 02-19-2008 | 0 | 15 | -18.7 | -20.8 | 82 | 1008.2 |
| 02-20-2008 | 1 | 7 | -16.5 | -20.3 | 70 | 999.2 |
| 02-21-2008 | 0 | 10 | -6.7 | -9.1 | 81 | 988.0 |
| 02-22-2008 | 0 | 6 | -4.4 | -7.4 | 77 | 995.6 |
| 02-23-2008 | 0 | 5 | -9.3 | -12.9 | 72 | 1005.8 |
| 02-24-2008 | 1 | 13 | -6.5 | -15.0 | 47 | 1007.7 |
| 02-25-2008 | 0 | 14 | -5.1 | -10.7 | 61 | 1003.4 |
| 02-26-2008 | 0 | 16 | -4.3 | -9.7 | 63 | 999.7 |
| 02-27-2008 | 0 | 14 | 0.5 | -5.6 | 63 | 993.6 |
| 02-28-2008 | 0 | 11 | -2.3 | -4.6 | 82 | 992.1 |
| 02-29-2008 | 0 | 9 | -2.5 | -6.1 | 74 | 998.7 |
| 03-01-2008 | 0 | 7 | -2.2 | -8.4 | 59 | 997.3 |
| 03-02-2008 | 1 | 11 | -2.9 | -6.8 | 72 | 1000.5 |
| 03-03-2008 | 1 | 15 | -5.8 | -7.7 | 86 | 1008.0 |
| 03-04-2008 | 0 | 18 | -6.8 | -9.5 | 82 | 1002.0 |
| 03-05-2008 | 1 | 13 | -5.6 | -8.9 | 79 | 995.5 |
| 03-06-2008 | 1 | 9 | 0.2 | -3.4 | 77 | 993.6 |
| 03-07-2008 | 0 | 9 | -2.0 | -6.1 | 71 | 1002.0 |
| 03-08-2008 | 1 | 5 | -0.1 | -1.1 | 92 | 994.6 |
| 03-09-2008 | 1 | 9 | 1.1 | -1.4 | 82 | 986.2 |
| 03-10-2008 | 1 | 10 | -2.6 | -5.1 | 81 | 994.7 |
| 03-11-2008 | 0 | 11 | -9.3 | -12.3 | 78 | 1007.3 |
| 03-12-2008 | 0 | 10 | -6.0 | -8.7 | 80 | 984.3 |
| 03-13-2008 | 0 | 14 | -2.7 | -6.5 | 73 | 991.6 |
| 03-14-2008 | 0 | 11 | -2.6 | -6.1 | 76 | 993.5 |
| 03-15-2008 | 1 | 13 | -1.7 | -4.9 | 79 | 985.8 |
| 03-16-2008 | 1 | 9 | -12.3 | -20.0 | 50 | 998.7 |
| 03-17-2008 | 2 | 5 | -5.9 | -11.4 | 65 | 990.0 |
| 03-18-2008 | 1 | 10 | 2.4 | -0.2 | 82 | 986.5 |
| 03-19-2008 | 1 | 6 | -0.1 | -1.9 | 87 | 990.0 |
| 03-20-2008 | 2 | 6 | -1.8 | -3.8 | 84 | 996.6 |
| 03-21-2008 | 1 | 13 | 1.2 | -1.9 | 80 | 997.2 |
| 03-22-2008 | 1 | 7 | 4.3 | -0.7 | 72 | 992.2 |
| 03-23-2008 | 1 | 12 | 7.6 | -0.1 | 61 | 986.5 |
| 03-24-2008 | 1 | 16 | 0.6 | -3.5 | 73 | 998.5 |
| 03-25-2008 | 0 | 10 | -1.5 | -6.7 | 68 | 1012.4 |
| 03-26-2008 | 0 | 15 | -1.0 | -8.2 | 60 | 1013.6 |
| 03-27-2008 | 0 | 7 | 1.3 | -7.5 | 52 | 1014.2 |
| 03-28-2008 | 0 | 11 | 3.9 | -5.7 | 51 | 1013.2 |
| 03-29-2008 | 0 | 9 | 2.7 | -5.8 | 53 | 1008.3 |
| 03-30-2008 | 1 | 9 | 3.6 | -4.4 | 56 | 1007.0 |
| 03-31-2008 | 0 | 12 | 3.5 | -4.7 | 56 | 1008.5 |
| 04-01-2008 | 0 | 11 | 3.9 | -5.9 | 49 | 1002.8 |
| 04-02-2008 | 0 | 7 | 5.1 | -4.3 | 53 | 994.0 |
| 04-03-2008 | 0 | 7 | 5.2 | -3.9 | 55 | 987.6 |
| 04-04-2008 | 0 | 16 | 6.1 | -0.1 | 65 | 985.0 |
| 04-05-2008 | 0 | 7 | 3.2 | -3.1 | 62 | 985.5 |
| 04-06-2008 | 0 | 9 | -6.5 | -13.5 | 54 | 1004.9 |
| 04-07-2008 | 0 | 8 | -3.4 | -12.2 | 49 | 1003.9 |
| 04-08-2008 | 1 | 7 | -0.3 | -7.0 | 58 | 999.4 |
| 04-09-2008 | 0 | 9 | -4.8 | -6.1 | 89 | 1000.0 |
| 04-10-2008 | 0 | 5 | -0.2 | -6.8 | 62 | 1001.0 |
| 04-11-2008 | 0 | 10 | 9.8 | 0.7 | 53 | 994.1 |
| 04-12-2008 | 0 | 7 | 13.0 | 3.6 | 55 | 990.8 |
| 04-13-2008 | 1 | 10 | 13.7 | 2.2 | 48 | 985.0 |
| 04-14-2008 | 1 | 10 | 12.5 | 0.7 | 49 | 974.5 |
| 04-15-2008 | 1 | 8 | -2.4 | -9.6 | 55 | 990.9 |
| 04-16-2008 | 1 | 7 | -2.3 | -3.9 | 88 | 981.5 |
| 04-17-2008 | 1 | 8 | -6.6 | -8.4 | 85 | 986.3 |
| 04-18-2008 | 2 | 9 | -8.6 | -13.1 | 67 | 1005.9 |
| 04-19-2008 | 0 | 10 | -0.4 | -8.3 | 54 | 1009.0 |
| 04-20-2008 | 0 | 8 | 4.8 | -3.1 | 57 | 1007.7 |
| 04-21-2008 | 0 | 12 | 7.3 | -2.6 | 52 | 1003.6 |
| 04-22-2008 | 2 | 9 | 12.0 | -3.0 | 38 | 998.1 |
| 04-23-2008 | 0 | 4 | 15.8 | 0.9 | 37 | 991.4 |
| 04-24-2008 | 0 | 15 | 16.7 | 6.7 | 53 | 992.4 |
| 04-25-2008 | 0 | 6 | 16.1 | 7.7 | 58 | 986.4 |
| 04-26-2008 | 0 | 9 | 6.9 | -1.6 | 58 | 993.0 |
| 04-27-2008 | 0 | 5 | 9.2 | -2.7 | 44 | 985.1 |
| 04-28-2008 | 0 | 7 | 2.7 | -6.2 | 53 | 984.5 |
| 04-29-2008 | 0 | 9 | 3.3 | -3.2 | 65 | 986.0 |
| 04-30-2008 | 0 | 9 | 1.8 | -0.2 | 86 | 983.3 |
| 05-01-2008 | 3 | 9 | 2.1 | -4.2 | 64 | 995.3 |
| 05-02-2008 | 1 | 7 | 4.2 | -0.1 | 75 | 994.5 |
| 05-03-2008 | 0 | 9 | 5.0 | 2.9 | 87 | 984.0 |
| 05-04-2008 | 1 | 6 | 6.2 | -0.3 | 65 | 989.7 |
| 05-05-2008 | 0 | 8 | 6.8 | -3.3 | 51 | 995.3 |
| 05-06-2008 | 1 | 11 | 9.1 | -2.3 | 46 | 998.8 |
| 05-07-2008 | 0 | 9 | 14.2 | -0.2 | 40 | 999.8 |
| 05-08-2008 | 2 | 10 | 15.5 | 0.7 | 38 | 993.9 |
| 05-09-2008 | 0 | 2 | 20.9 | 2.1 | 31 | 990.6 |
| 05-10-2008 | 0 | 9 | 22.5 | 6.3 | 38 | 987.3 |
| 05-11-2008 | 0 | 7 | 15.2 | 7.6 | 63 | 992.9 |
| 05-12-2008 | 2 | 17 | 14.5 | 3.4 | 49 | 994.2 |
| 05-13-2008 | 0 | 19 | 18.5 | 4.7 | 43 | 991.0 |
| 05-14-2008 | 0 | 13 | 19.5 | 4.4 | 38 | 991.9 |
| 05-15-2008 | 0 | 10 | 18.5 | 5.6 | 45 | 989.7 |
| 05-16-2008 | 2 | 23 | 20.6 | 6.3 | 41 | 990.1 |
| 05-17-2008 | 1 | 4 | 17.1 | 10.3 | 64 | 995.8 |
| 05-18-2008 | 0 | 9 | 16.3 | 3.2 | 44 | 999.7 |
| 05-19-2008 | 0 | 12 | 18.7 | 5.8 | 47 | 990.5 |
| 05-20-2008 | 0 | 11 | 13.7 | 5.1 | 58 | 989.4 |
| 05-21-2008 | 0 | 7 | 8.9 | 5.4 | 79 | 989.8 |
| 05-22-2008 | 0 | 8 | 11.4 | 0.4 | 50 | 990.0 |
| 05-23-2008 | 0 | 3 | 14.9 | 6.7 | 63 | 983.0 |
| 05-24-2008 | 0 | 1 | 7.2 | 1.1 | 66 | 991.5 |
| 05-25-2008 | 0 | 5 | 5.1 | -3.8 | 53 | 1000.5 |
| 05-26-2008 | 1 | 8 | 7.3 | -0.4 | 60 | 995.2 |
| 05-27-2008 | 0 | 2 | 12.7 | -1.2 | 42 | 996.9 |
| 05-28-2008 | 1 | 5 | 19.2 | 7.6 | 47 | 988.4 |
| 05-29-2008 | 0 | 6 | 20.9 | 7.8 | 49 | 988.0 |
| 05-30-2008 | 0 | 7 | 20.1 | 12.4 | 62 | 986.1 |
| 05-31-2008 | 0 | 2 | 26.0 | 14.1 | 50 | 984.8 |
| 06-01-2008 | 0 | 9 | 22.8 | 17.0 | 72 | 986.7 |
| 06-02-2008 | 3 | 8 | 19.4 | 10.1 | 58 | 993.0 |
| 06-03-2008 | 1 | 7 | 22.2 | 9.7 | 47 | 990.8 |
| 06-04-2008 | 4 | 1 | 20.0 | 10.5 | 55 | 990.3 |
| 06-05-2008 | 0 | 8 | 25.2 | 11.0 | 47 | 976.0 |
| 06-06-2008 | 1 | 7 | 14.4 | 10.8 | 79 | 982.7 |
| 06-07-2008 | 0 | 8 | 20.3 | 8.2 | 47 | 985.1 |
| 06-08-2008 | 1 | 5 | 21.9 | 11.4 | 54 | 981.5 |
| 06-09-2008 | 2 | 5 | 19.0 | 12.7 | 69 | 981.3 |
| 06-10-2008 | 2 | 9 | 11.6 | 10.1 | 90 | 982.5 |
| 06-11-2008 | 1 | 9 | 13.8 | 4.0 | 53 | 990.5 |
| 06-12-2008 | 0 | 7 | 14.9 | 8.6 | 68 | 985.2 |
| 06-13-2008 | 0 | 4 | 15.0 | 8.2 | 68 | 991.2 |
| 06-14-2008 | 0 | 8 | 15.1 | 10.1 | 75 | 990.0 |
| 06-15-2008 | 0 | 4 | 17.4 | 9.8 | 64 | 984.7 |
| 06-16-2008 | 0 | 11 | 17.3 | 11.9 | 73 | 978.6 |
| 06-17-2008 | 3 | 1 | 12.0 | 9.3 | 84 | 982.5 |
| 06-18-2008 | 1 | 4 | 13.3 | 12.1 | 92 | 983.8 |
| 06-19-2008 | 0 | 8 | 15.7 | 12.4 | 82 | 990.0 |
| 06-20-2008 | 5 | 4 | 19.6 | 15.3 | 77 | 990.6 |
| 06-21-2008 | 1 | 4 | 21.2 | 14.4 | 68 | 987.8 |
| 06-22-2008 | 1 | 3 | 21.9 | 16.2 | 72 | 984.1 |
| 06-23-2008 | 5 | 3 | 19.6 | 15.0 | 75 | 984.6 |
| 06-24-2008 | 0 | 8 | 18.3 | 10.7 | 62 | 988.0 |
| 06-25-2008 | 1 | 1 | 17.8 | 9.8 | 61 | 988.0 |
| 06-26-2008 | 0 | 7 | 18.3 | 9.7 | 60 | 988.2 |
| 06-27-2008 | 2 | 7 | 21.9 | 12.2 | 57 | 984.0 |
| 06-28-2008 | 0 | 4 | 20.5 | 14.3 | 70 | 978.5 |
| 06-29-2008 | 0 | 3 | 18.7 | 12.1 | 67 | 978.7 |
| 06-30-2008 | 1 | 4 | 19.4 | 14.1 | 72 | 980.7 |
| 07-01-2008 | 1 | 8 | 20.3 | 13.7 | 68 | 984.6 |
| 07-02-2008 | 2 | 5 | 20.8 | 11.6 | 60 | 985.7 |
| 07-03-2008 | 1 | 11 | 23.4 | 14.6 | 59 | 978.5 |
| 07-04-2008 | 1 | 4 | 20.9 | 13.8 | 65 | 975.2 |
| 07-05-2008 | 1 | 3 | 16.3 | 12.6 | 79 | 978.7 |
| 07-06-2008 | 3 | 5 | 16.9 | 12.3 | 74 | 977.4 |
| 07-07-2008 | 1 | 8 | 15.9 | 12.9 | 82 | 983.3 |
| 07-08-2008 | 3 | 9 | 16.5 | 9.7 | 66 | 991.6 |
| 07-09-2008 | 1 | 3 | 18.1 | 11.1 | 65 | 991.8 |
| 07-10-2008 | 0 | 4 | 23.2 | 13.5 | 55 | 988.7 |
| 07-11-2008 | 0 | 10 | 22.4 | 16.9 | 73 | 989.2 |
| 07-12-2008 | 1 | 1 | 20.3 | 15.9 | 77 | 986.3 |
| 07-13-2008 | 2 | 3 | 20.4 | 11.9 | 62 | 984.5 |
| 07-14-2008 | 2 | 3 | 22.9 | 14.2 | 61 | 982.2 |
| 07-15-2008 | 3 | 3 | 24.3 | 15.5 | 61 | 980.5 |
| 07-16-2008 | 1 | 8 | 24.9 | 16.0 | 59 | 981.0 |
| 07-17-2008 | 0 | 13 | 23.6 | 13.2 | 54 | 983.0 |
| 07-18-2008 | 0 | 7 | 24.9 | 15.4 | 58 | 980.5 |
| 07-19-2008 | 2 | 8 | 21.4 | 18.6 | 85 | 979.6 |
| 07-20-2008 | 3 | 5 | 21.1 | 18.9 | 88 | 982.2 |
| 07-21-2008 | 1 | 5 | 20.8 | 17.6 | 83 | 982.1 |
| 07-22-2008 | 1 | 2 | 21.1 | 17.5 | 80 | 981.9 |
| 07-23-2008 | 0 | 9 | 18.0 | 10.2 | 62 | 981.7 |
| 07-24-2008 | 0 | 6 | 15.6 | 11.2 | 76 | 984.3 |
| 07-25-2008 | 2 | 5 | 17.6 | 11.4 | 69 | 986.3 |
| 07-26-2008 | 0 | 10 | 20.9 | 11.2 | 55 | 987.8 |
| 07-27-2008 | 1 | 9 | 24.8 | 14.8 | 55 | 986.0 |
| 07-28-2008 | 4 | 6 | 25.8 | 16.8 | 58 | 986.2 |
| 07-29-2008 | 0 | 13 | 23.2 | 16.4 | 67 | 988.3 |
| 07-30-2008 | 3 | 8 | 25.4 | 15.5 | 56 | 987.9 |
| 07-31-2008 | 0 | 9 | 25.3 | 18.5 | 67 | 981.8 |
| 08-01-2008 | 1 | 13 | 22.1 | 13.4 | 60 | 958.1 |
| 08-02-2008 | 0 | 8 | 17.2 | 11.1 | 68 | 985.9 |
| 08-03-2008 | 0 | 5 | 19.5 | 11.7 | 63 | 980.0 |
| 08-04-2008 | 1 | 12 | 17.2 | 14.0 | 82 | 980.5 |
| 08-05-2008 | 2 | 10 | 20.3 | 11.5 | 59 | 988.5 |
| 08-06-2008 | 2 | 8 | 21.9 | 12.3 | 57 | 984.4 |
| 08-07-2008 | 0 | 8 | 17.7 | 7.8 | 56 | 989.1 |
| 08-08-2008 | 2 | 6 | 19.2 | 9.9 | 58 | 984.0 |
| 08-09-2008 | 2 | 9 | 25.1 | 13.3 | 52 | 978.0 |
| 08-10-2008 | 0 | 8 | 19.8 | 14.0 | 72 | 982.2 |
| 08-11-2008 | 3 | 13 | 15.6 | 10.7 | 75 | 993.7 |
| 08-12-2008 | 2 | 16 | 19.5 | 9.8 | 56 | 994.0 |
| 08-13-2008 | 1 | 12 | 20.4 | 11.9 | 61 | 992.3 |
| 08-14-2008 | 0 | 14 | 19.7 | 11.0 | 62 | 988.8 |
| 08-15-2008 | 0 | 11 | 19.8 | 10.7 | 60 | 981.5 |
| 08-16-2008 | 2 | 4 | 17.2 | 11.6 | 71 | 981.2 |
| 08-17-2008 | 0 | 10 | 15.4 | 12.9 | 85 | 982.1 |
| 08-18-2008 | 2 | 10 | 14.9 | 11.9 | 83 | 986.9 |
| 08-19-2008 | 2 | 10 | 15.2 | 11.1 | 77 | 987.7 |
| 08-20-2008 | 1 | 14 | 12.6 | 11.6 | 94 | 987.9 |
| 08-21-2008 | 0 | 3 | 10.2 | 5.4 | 76 | 991.8 |
| 08-22-2008 | 0 | 7 | 10.5 | 4.8 | 71 | 989.5 |
| 08-23-2008 | 1 | 7 | 10.3 | 5.3 | 74 | 988.2 |
| 08-24-2008 | 0 | 2 | 12.1 | 5.7 | 69 | 995.6 |
| 08-25-2008 | 3 | 13 | 14.4 | 6.7 | 62 | 987.3 |
| 08-26-2008 | 3 | 6 | 10.1 | 9.7 | 97 | 983.3 |
| 08-27-2008 | 3 | 11 | 12.7 | 6.2 | 67 | 995.8 |
| 08-28-2008 | 0 | 7 | 18.7 | 9.2 | 56 | 991.2 |
| 08-29-2008 | 0 | 3 | 21.1 | 11.3 | 56 | 984.6 |
| 08-30-2008 | 0 | 3 | 20.7 | 12.8 | 62 | 982.3 |
| 08-31-2008 | 2 | 10 | 17.1 | 11.4 | 72 | 991.2 |
| 09-01-2008 | 0 | 13 | 16.6 | 9.4 | 66 | 991.9 |
| 09-02-2008 | 1 | 7 | 16.4 | 9.0 | 66 | 987.4 |
| 09-03-2008 | 0 | 16 | 12.5 | 8.4 | 77 | 989.7 |
| 09-04-2008 | 2 | 5 | 11.8 | 3.3 | 62 | 996.2 |
| 09-05-2008 | 0 | 8 | 13.1 | 6.6 | 67 | 990.7 |
| 09-06-2008 | 1 | 4 | 12.2 | 6.7 | 72 | 991.4 |
| 09-07-2008 | 0 | 4 | 11.2 | 5.8 | 71 | 994.6 |
| 09-08-2008 | 1 | 11 | 14.3 | 7.0 | 65 | 987.7 |
| 09-09-2008 | 2 | 8 | 13.1 | 10.5 | 84 | 981.2 |
| 09-10-2008 | 1 | 11 | 15.3 | 9.4 | 69 | 983.2 |
| 09-11-2008 | 0 | 5 | 12.1 | 6.1 | 67 | 987.9 |
| 09-12-2008 | 1 | 8 | 9.1 | 4.3 | 74 | 995.9 |
| 09-13-2008 | 0 | 5 | 8.9 | 4.9 | 78 | 997.9 |
| 09-14-2008 | 0 | 1 | 10.3 | 3.4 | 65 | 992.7 |
| 09-15-2008 | 0 | 8 | 8.5 | 6.7 | 88 | 982.5 |
| 09-16-2008 | 1 | 7 | 6.3 | 0.7 | 68 | 989.4 |
| 09-17-2008 | 0 | 7 | 5.5 | 4.9 | 96 | 976.2 |
| 09-18-2008 | 3 | 8 | 4.6 | 2.8 | 89 | 989.0 |
| 09-19-2008 | 0 | 0 | 3.5 | 2.2 | 91 | 994.8 |
| 09-20-2008 | 0 | 4 | 5.0 | 3.2 | 88 | 1001.4 |
| 09-21-2008 | 0 | 6 | 6.2 | 2.3 | 78 | 1002.7 |
| 09-22-2008 | 1 | 2 | 6.7 | 1.2 | 70 | 1003.9 |
| 09-23-2008 | 2 | 5 | 3.8 | -0.1 | 77 | 1010.2 |
| 09-24-2008 | 2 | 7 | 5.5 | -0.3 | 68 | 1007.3 |
| 09-25-2008 | 0 | 7 | 6.4 | 0.2 | 68 | 997.1 |
| 09-26-2008 | 2 | 9 | 4.3 | -0.1 | 75 | 994.4 |
| 09-27-2008 | 0 | 1 | 7.0 | 0.4 | 65 | 990.7 |
| 09-28-2008 | 0 | 2 | 9.1 | -0.0 | 55 | 987.7 |
| 09-29-2008 | 0 | 6 | 2.9 | -3.0 | 65 | 997.1 |
| 09-30-2008 | 0 | 8 | 2.4 | -5.3 | 60 | 1000.5 |
| 10-01-2008 | 1 | 5 | 2.7 | -6.3 | 55 | 1004.4 |
| 10-02-2008 | 1 | 3 | 5.8 | -3.2 | 56 | 1003.5 |
| 10-03-2008 | 0 | 5 | 8.1 | -1.1 | 56 | 1001.7 |
| 10-04-2008 | 0 | 6 | 9.4 | -0.8 | 53 | 997.3 |
| 10-05-2008 | 1 | 6 | 11.1 | 1.5 | 52 | 996.6 |
| 10-06-2008 | 1 | 15 | 9.3 | 4.4 | 72 | 999.1 |
| 10-07-2008 | 2 | 7 | 11.1 | 3.4 | 61 | 992.8 |
| 10-08-2008 | 0 | 6 | 3.2 | -0.8 | 76 | 1000.8 |
| 10-09-2008 | 0 | 7 | 3.4 | -1.1 | 75 | 997.2 |
| 10-10-2008 | 1 | 2 | 9.5 | -0.5 | 54 | 992.0 |
| 10-11-2008 | 0 | 11 | 15.6 | 4.3 | 50 | 982.9 |
| 10-12-2008 | 0 | 2 | 4.7 | -1.3 | 65 | 992.9 |
| 10-13-2008 | 0 | 6 | 3.6 | -2.2 | 65 | 994.3 |
| 10-14-2008 | 0 | 6 | 7.3 | 0.2 | 62 | 991.5 |
| 10-15-2008 | 0 | 8 | 7.7 | 4.9 | 82 | 985.5 |
| 10-16-2008 | 0 | 8 | 6.0 | 1.6 | 73 | 995.9 |
| 10-17-2008 | 0 | 9 | 6.5 | 4.2 | 85 | 987.7 |
| 10-18-2008 | 1 | 5 | 2.4 | -0.6 | 80 | 993.0 |
| 10-19-2008 | 0 | 3 | -0.3 | -3.3 | 80 | 1000.2 |
| 10-20-2008 | 0 | 7 | -3.8 | -8.6 | 67 | 1010.6 |
| 10-21-2008 | 1 | 6 | -2.0 | -7.6 | 65 | 1010.1 |
| 10-22-2008 | 0 | 4 | 0.8 | -6.0 | 63 | 1004.2 |
| 10-23-2008 | 1 | 5 | 4.7 | -4.2 | 53 | 999.8 |
| 10-24-2008 | 0 | 4 | 2.0 | -3.8 | 66 | 1000.8 |
| 10-25-2008 | 0 | 8 | 1.0 | -3.9 | 71 | 1003.0 |
| 10-26-2008 | 0 | 14 | 4.3 | -1.7 | 68 | 1002.2 |
| 10-27-2008 | 1 | 9 | 4.2 | 2.0 | 85 | 998.5 |
| 10-28-2008 | 0 | 5 | 5.5 | 3.3 | 86 | 995.7 |
| 10-29-2008 | 0 | 6 | 3.7 | -0.7 | 73 | 1000.3 |
| 10-30-2008 | 0 | 6 | 5.3 | -1.0 | 65 | 1000.3 |
| 10-31-2008 | 0 | 3 | 3.4 | 0.0 | 78 | 1003.7 |
| 11-01-2008 | 0 | 4 | 4.8 | 0.0 | 71 | 998.5 |
| 11-02-2008 | 0 | 5 | 7.0 | -1.4 | 55 | 992.5 |
| 11-03-2008 | 0 | 5 | 5.2 | 2.9 | 86 | 992.4 |
| 11-04-2008 | 0 | 2 | 5.8 | 2.3 | 78 | 992.0 |
| 11-05-2008 | 0 | 7 | 6.3 | 0.7 | 69 | 988.8 |
| 11-06-2008 | 0 | 6 | 1.6 | -1.7 | 79 | 993.7 |
| 11-07-2008 | 0 | 5 | 7.6 | 4.4 | 80 | 987.2 |
| 11-08-2008 | 0 | 5 | 1.8 | 0.5 | 91 | 987.3 |
| 11-09-2008 | 0 | 9 | -5.5 | -9.2 | 72 | 1002.3 |
| 11-10-2008 | 1 | 8 | -4.1 | -6.4 | 82 | 1002.2 |
| 11-11-2008 | 0 | 3 | -3.5 | -5.1 | 87 | 1000.8 |
| 11-12-2008 | 1 | 7 | -2.1 | -2.9 | 93 | 996.8 |
| 11-13-2008 | 0 | 8 | -0.6 | -1.5 | 93 | 993.3 |
| 11-14-2008 | 2 | 4 | -0.3 | -0.9 | 95 | 995.3 |
| 11-15-2008 | 0 | 2 | -0.9 | -2.0 | 91 | 1005.7 |
| 11-16-2008 | 0 | 3 | -2.1 | -3.8 | 86 | 1008.3 |
| 11-17-2008 | 1 | 4 | -5.4 | -8.1 | 79 | 1005.6 |
| 11-18-2008 | 2 | 9 | -5.9 | -10.6 | 67 | 1005.6 |
| 11-19-2008 | 0 | 6 | -9.3 | -12.5 | 76 | 1004.5 |
| 11-20-2008 | 1 | 5 | -8.8 | -11.1 | 83 | 1001.7 |
| 11-21-2008 | 0 | 4 | -6.3 | -7.6 | 90 | 1003.9 |
| 11-22-2008 | 1 | 3 | -6.5 | -8.9 | 81 | 1002.6 |
| 11-23-2008 | 0 | 6 | -0.7 | -4.9 | 72 | 1000.6 |
| 11-24-2008 | 1 | 8 | 0.8 | -0.7 | 89 | 1010.6 |
| 11-25-2008 | 0 | 7 | -3.2 | -4.7 | 90 | 1008.4 |
| 11-26-2008 | 0 | 8 | -3.6 | -7.9 | 70 | 1002.9 |
| 11-27-2008 | 1 | 3 | -3.3 | -6.3 | 78 | 1003.2 |
| 11-28-2008 | 0 | 10 | 2.3 | -7.3 | 48 | 994.5 |
| 11-29-2008 | 0 | 5 | -2.7 | -5.9 | 76 | 995.1 |
| 11-30-2008 | 0 | 5 | -2.9 | -4.6 | 86 | 996.6 |
| 12-01-2008 | 0 | 5 | -18.4 | -21.7 | 73 | 1009.0 |
| 12-02-2008 | 0 | 10 | -28.6 | -31.3 | 76 | 1026.3 |
| 12-03-2008 | 0 | 3 | -24.1 | -27.2 | 73 | 1022.8 |
| 12-04-2008 | 0 | 5 | -15.5 | -20.5 | 63 | 1013.8 |
| 12-05-2008 | 1 | 6 | -7.7 | -11.0 | 75 | 1004.2 |
| 12-06-2008 | 0 | 6 | -2.5 | -4.2 | 86 | 999.8 |
| 12-07-2008 | 1 | 2 | -0.8 | -4.0 | 77 | 990.8 |
| 12-08-2008 | 0 | 7 | -2.2 | -3.0 | 93 | 992.8 |
| 12-09-2008 | 0 | 7 | -0.1 | -1.1 | 92 | 997.2 |
| 12-10-2008 | 0 | 3 | -2.2 | -4.8 | 80 | 998.7 |
| 12-11-2008 | 2 | 6 | -15.1 | -17.6 | 79 | 1014.3 |
| 12-12-2008 | 1 | 2 | -15.8 | -17.4 | 86 | 1012.5 |
| 12-13-2008 | 0 | 3 | -14.7 | -17.0 | 81 | 998.0 |
| 12-14-2008 | 0 | 2 | -7.4 | -10.4 | 77 | 990.9 |
| 12-15-2008 | 1 | 8 | -22.9 | -26.0 | 73 | 1004.7 |
| 12-16-2008 | 1 | 4 | -15.1 | -17.1 | 82 | 1001.3 |
| 12-17-2008 | 1 | 3 | -9.9 | -12.7 | 78 | 1000.9 |
| 12-18-2008 | 0 | 5 | -18.0 | -20.0 | 82 | 1015.5 |
| 12-19-2008 | 0 | 0 | -17.6 | -19.6 | 82 | 1018.7 |
| 12-20-2008 | 1 | 4 | -18.8 | -20.7 | 83 | 1016.7 |
| 12-21-2008 | 0 | 2 | -23.4 | -25.5 | 80 | 1012.6 |
| 12-22-2008 | 1 | 5 | -22.7 | -24.7 | 81 | 1007.4 |
| 12-23-2008 | 1 | 5 | -17.5 | -19.3 | 84 | 1005.2 |
| 12-24-2008 | 1 | 6 | -17.7 | -19.4 | 85 | 1005.6 |
| 12-25-2008 | 1 | 3 | -20.8 | -22.7 | 82 | 1006.8 |
| 12-26-2008 | 0 | 7 | -18.8 | -20.4 | 85 | 1011.5 |
| 12-27-2008 | 1 | 6 | -21.1 | -23.2 | 81 | 1011.4 |
| 12-28-2008 | 1 | 1 | -21.3 | -24.1 | 76 | 1005.3 |
| 12-29-2008 | 0 | 3 | -11.2 | -13.6 | 81 | 1000.3 |
| 12-30-2008 | 0 | 2 | -11.9 | -14.9 | 76 | 1005.1 |
| 12-31-2008 | 0 | 8 | -11.6 | -17.0 | 62 | 1005.5 |
| 01-01-2009 | 1 | 4 | -12.7 | -18.2 | 60 | 1006.8 |
| 01-03-2009 | 1 | 4 | -17.8 | -20.4 | 78 | 996.1 |
| 01-04-2009 | 0 | 8 | -11.4 | -14.2 | 77 | 1007.0 |
| 01-05-2009 | 0 | 4 | -11.5 | -13.9 | 80 | 1003.7 |
| 01-06-2009 | 0 | 5 | -17.1 | -20.2 | 74 | 1010.3 |
| 01-07-2009 | 1 | 2 | -13.2 | -17.2 | 70 | 997.8 |
| 01-08-2009 | 0 | 10 | -12.9 | -15.5 | 79 | 1008.6 |
| 01-09-2009 | 0 | 5 | -20.6 | -22.6 | 82 | 1012.4 |
| 01-10-2009 | 0 | 5 | -16.5 | -18.7 | 81 | 1003.4 |
| 01-11-2009 | 1 | 5 | -9.3 | -11.2 | 84 | 1005.4 |
| 01-12-2009 | 1 | 5 | -6.2 | -10.1 | 71 | 1006.4 |
| 01-13-2009 | 0 | 10 | -9.6 | -11.8 | 83 | 1005.5 |
| 01-14-2009 | 0 | 4 | -8.8 | -12.4 | 74 | 996.4 |
| 01-15-2009 | 0 | 13 | -11.9 | -14.5 | 79 | 994.2 |
| 01-16-2009 | 0 | 10 | -11.2 | -13.7 | 79 | 996.7 |
| 01-17-2009 | 0 | 4 | -14.2 | -15.8 | 86 | 997.3 |
| 01-18-2009 | 0 | 7 | -12.3 | -14.8 | 79 | 994.2 |
| 01-19-2009 | 0 | 3 | -14.5 | -16.7 | 81 | 997.6 |
| 01-20-2009 | 1 | 5 | -24.6 | -26.8 | 80 | 1017.0 |
| 01-21-2009 | 0 | 6 | -28.5 | -31.3 | 75 | 1030.7 |
| 01-22-2009 | 0 | 9 | -23.3 | -27.0 | 69 | 1025.9 |
| 01-23-2009 | 1 | 5 | -24.0 | -27.3 | 72 | 1011.7 |
| 01-24-2009 | 0 | 5 | -20.1 | -23.6 | 72 | 1006.2 |
| 01-25-2009 | 0 | 9 | -7.1 | -12.2 | 64 | 995.2 |
| 01-26-2009 | 1 | 6 | -8.3 | -9.9 | 87 | 990.6 |
| 01-27-2009 | 0 | 7 | -21.5 | -23.3 | 83 | 1000.7 |
| 01-28-2009 | 1 | 9 | -24.5 | -26.7 | 79 | 1001.7 |
| 01-29-2009 | 0 | 6 | -20.0 | -22.1 | 82 | 998.4 |
| 01-30-2009 | 1 | 4 | -19.9 | -21.7 | 84 | 999.9 |
| 01-31-2009 | 0 | 8 | -28.1 | -30.3 | 80 | 1011.5 |
| 02-01-2009 | 0 | 5 | -26.7 | -28.8 | 80 | 1002.1 |
| 02-03-2009 | 1 | 10 | -15.5 | -17.2 | 85 | 982.8 |
| 02-04-2009 | 0 | 2 | -7.3 | -9.9 | 80 | 985.5 |
| 02-05-2009 | 1 | 4 | -8.6 | -11.4 | 78 | 992.2 |
| 02-06-2009 | 0 | 8 | -4.8 | -8.2 | 74 | 993.3 |
| 02-07-2009 | 1 | 2 | -5.3 | -7.2 | 85 | 995.8 |
| 02-08-2009 | 0 | 3 | -6.5 | -7.8 | 89 | 990.6 |
| 02-09-2009 | 1 | 6 | -22.6 | -26.4 | 69 | 999.2 |
| 02-10-2009 | 0 | 8 | -25.2 | -27.8 | 76 | 994.4 |
| 02-11-2009 | 0 | 7 | -29.0 | -31.4 | 77 | 1005.2 |
| 02-12-2009 | 0 | 8 | -33.8 | -36.8 | 72 | 1008.1 |
| 03-01-2009 | 0 | 8 | -15.1 | -18.5 | 74 | 1007.9 |
| 03-03-2009 | 0 | 11 | -13.6 | -16.2 | 80 | 1002.4 |
| 03-04-2009 | 1 | 11 | -11.8 | -15.2 | 75 | 1001.5 |
| 03-05-2009 | 1 | 10 | -7.8 | -11.9 | 72 | 995.1 |
| 03-06-2009 | 1 | 11 | -7.9 | -10.6 | 79 | 995.7 |
| 03-07-2009 | 1 | 9 | -7.3 | -12.6 | 63 | 1001.2 |
| 03-08-2009 | 0 | 14 | -9.5 | -14.3 | 68 | 1000.4 |
| 03-09-2009 | 0 | 8 | -8.9 | -10.8 | 85 | 1001.5 |
| 03-10-2009 | 0 | 11 | -13.2 | -18.0 | 66 | 1011.5 |
| 03-11-2009 | 0 | 9 | -12.7 | -16.7 | 70 | 1016.1 |
| 03-12-2009 | 1 | 10 | -5.3 | -11.6 | 58 | 995.8 |
| 03-13-2009 | 0 | 9 | 0.9 | -1.0 | 87 | 984.3 |
| 03-14-2009 | 1 | 6 | -1.0 | -2.1 | 91 | 993.4 |
| 03-15-2009 | 1 | 10 | -4.2 | -7.8 | 74 | 986.0 |
| 03-16-2009 | 1 | 10 | -9.2 | -13.9 | 67 | 984.0 |
| 03-17-2009 | 1 | 13 | -13.1 | -17.7 | 66 | 994.3 |
| 03-18-2009 | 0 | 6 | -12.0 | -15.2 | 76 | 993.4 |
| 03-19-2009 | 0 | 5 | -10.2 | -12.4 | 82 | 997.1 |
| 03-20-2009 | 2 | 8 | -12.1 | -15.9 | 74 | 1006.0 |
| 03-21-2009 | 0 | 2 | -12.6 | -16.0 | 76 | 1002.9 |
| 03-22-2009 | 0 | 7 | -9.1 | -13.6 | 70 | 997.8 |
| 03-23-2009 | 0 | 5 | -0.0 | -6.2 | 66 | 993.2 |
| 03-24-2009 | 1 | 10 | -0.4 | -3.2 | 80 | 1000.2 |
| 03-25-2009 | 0 | 3 | -0.7 | -3.6 | 81 | 1008.3 |
| 03-26-2009 | 1 | 6 | 2.1 | -3.5 | 67 | 1003.3 |
| 03-27-2009 | 1 | 6 | 3.8 | -2.7 | 64 | 999.8 |
| 03-28-2009 | 1 | 4 | 3.0 | -4.4 | 59 | 999.7 |
| 03-29-2009 | 0 | 2 | 5.5 | -2.3 | 59 | 997.6 |
| 03-30-2009 | 0 | 10 | 4.5 | -1.4 | 66 | 1000.4 |
| 03-31-2009 | 0 | 11 | 2.7 | -3.9 | 62 | 1000.8 |
| 04-01-2009 | 0 | 21 | 1.6 | 0.1 | 90 | 995.9 |
| 04-03-2009 | 0 | 11 | 7.6 | -0.4 | 58 | 991.1 |
| 04-04-2009 | 1 | 9 | 9.0 | 3.6 | 69 | 989.8 |
| 04-05-2009 | 1 | 9 | 3.9 | -1.6 | 68 | 991.5 |
| 04-06-2009 | 2 | 18 | 5.3 | 0.5 | 72 | 983.0 |
| 04-07-2009 | 0 | 12 | 1.3 | -2.2 | 78 | 992.7 |
| 04-08-2009 | 0 | 8 | 3.2 | -4.8 | 57 | 996.4 |
| 04-09-2009 | 0 | 4 | 6.9 | -1.7 | 57 | 992.2 |
| 04-10-2009 | 1 | 7 | 0.8 | -8.2 | 53 | 1002.8 |
| 04-11-2009 | 2 | 3 | 2.9 | -6.8 | 50 | 998.6 |
| 04-12-2009 | 2 | 9 | 5.3 | -2.6 | 58 | 994.7 |
| 04-13-2009 | 3 | 5 | 6.6 | -1.7 | 57 | 996.3 |
| 04-14-2009 | 0 | 5 | 5.4 | 1.8 | 78 | 992.8 |
| 04-15-2009 | 2 | 9 | 2.6 | -4.4 | 61 | 992.6 |
| 04-16-2009 | 1 | 12 | 3.4 | -0.9 | 75 | 994.7 |
| 04-17-2009 | 0 | 8 | 3.6 | -1.4 | 72 | 995.2 |
| 04-18-2009 | 0 | 6 | 8.4 | -2.0 | 51 | 998.5 |
| 04-19-2009 | 1 | 4 | 3.4 | -7.9 | 45 | 1004.0 |
| 04-20-2009 | 3 | 10 | 6.6 | -10.6 | 30 | 995.2 |
| 04-21-2009 | 1 | 14 | 11.0 | -6.8 | 29 | 990.5 |
| 04-22-2009 | 1 | 15 | 6.9 | 0.4 | 69 | 997.7 |
| 04-23-2009 | 2 | 19 | 8.7 | -0.3 | 57 | 1002.9 |
| 04-24-2009 | 0 | 14 | 11.0 | 2.5 | 59 | 998.6 |
| 04-25-2009 | 3 | 15 | 5.6 | 4.2 | 91 | 991.9 |
| 04-26-2009 | 0 | 6 | 5.2 | 3.7 | 90 | 991.5 |
| 04-27-2009 | 0 | 12 | 7.8 | -0.4 | 58 | 996.2 |
| 04-28-2009 | 2 | 12 | 8.6 | 1.5 | 65 | 990.5 |
| 04-29-2009 | 1 | 5 | 7.1 | 0.9 | 65 | 989.1 |
| 04-30-2009 | 0 | 12 | 11.0 | 5.1 | 67 | 987.3 |
| 05-01-2009 | 0 | 11 | 12.2 | 6.6 | 69 | 989.2 |
| 05-03-2009 | 0 | 11 | 11.0 | 5.1 | 69 | 977.2 |
| 05-04-2009 | 5 | 15 | 5.7 | 0.7 | 71 | 990.2 |
| 05-05-2009 | 3 | 7 | 5.2 | -3.4 | 55 | 999.3 |
| 05-06-2009 | 2 | 13 | 5.8 | -0.9 | 64 | 999.2 |
| 05-07-2009 | 0 | 12 | 8.4 | -6.9 | 35 | 1002.9 |
| 05-08-2009 | 1 | 6 | 12.1 | -2.9 | 36 | 998.5 |
| 05-09-2009 | 1 | 9 | 16.0 | 3.1 | 42 | 996.2 |
| 05-10-2009 | 1 | 5 | 18.0 | 0.4 | 32 | 995.6 |
| 05-11-2009 | 0 | 6 | 19.9 | 2.6 | 34 | 994.6 |
| 05-12-2009 | 2 | 10 | 21.8 | 4.5 | 35 | 990.5 |
| 05-13-2009 | 0 | 6 | 15.3 | 2.1 | 42 | 992.7 |
| 05-14-2009 | 2 | 11 | 11.3 | -1.2 | 43 | 998.5 |
| 05-15-2009 | 0 | 10 | 14.6 | 3.0 | 48 | 995.8 |
| 05-16-2009 | 0 | 7 | 19.9 | 1.6 | 32 | 990.7 |
| 05-17-2009 | 2 | 9 | 19.7 | 9.0 | 52 | 985.3 |
| 05-18-2009 | 0 | 13 | 14.3 | 7.9 | 70 | 979.2 |
| 05-19-2009 | 0 | 2 | 12.6 | 1.4 | 50 | 988.8 |
| 05-20-2009 | 1 | 6 | 17.1 | 4.4 | 43 | 985.8 |
| 05-21-2009 | 0 | 5 | 15.1 | 7.1 | 62 | 979.4 |
| 05-22-2009 | 2 | 6 | 5.4 | 1.0 | 74 | 997.4 |
| 05-23-2009 | 0 | 2 | 5.2 | 4.5 | 95 | 993.3 |
| 05-24-2009 | 0 | 6 | 5.5 | 4.6 | 94 | 985.1 |
| 05-25-2009 | 2 | 14 | 5.6 | -2.2 | 58 | 998.8 |
| 05-26-2009 | 2 | 10 | 6.8 | 5.0 | 89 | 1000.5 |
| 05-27-2009 | 3 | 4 | 10.6 | 2.3 | 61 | 1001.9 |
| 05-28-2009 | 1 | 8 | 17.2 | 3.2 | 40 | 997.6 |
| 05-29-2009 | 0 | 9 | 23.0 | 7.8 | 42 | 988.7 |
| 05-30-2009 | 0 | 6 | 21.5 | 11.8 | 56 | 982.8 |
| 05-31-2009 | 1 | 4 | 16.2 | 13.6 | 85 | 980.3 |
| 06-01-2009 | 2 | 9 | 19.4 | 12.1 | 66 | 978.9 |
| 06-03-2009 | 3 | 9 | 15.4 | 9.6 | 69 | 987.2 |
| 06-04-2009 | 1 | 6 | 12.9 | 5.7 | 65 | 995.2 |
| 06-05-2009 | 0 | 11 | 15.3 | 4.6 | 52 | 996.2 |
| 06-06-2009 | 0 | 5 | 11.2 | 8.4 | 83 | 993.0 |
| 06-07-2009 | 1 | 4 | 17.0 | 6.9 | 54 | 991.7 |
| 06-08-2009 | 1 | 15 | 20.8 | 11.5 | 58 | 990.6 |
| 06-09-2009 | 0 | 8 | 20.1 | 12.9 | 65 | 992.0 |
| 06-10-2009 | 1 | 8 | 20.2 | 14.6 | 71 | 990.4 |
| 06-11-2009 | 0 | 12 | 19.0 | 14.9 | 77 | 984.9 |
| 06-12-2009 | 0 | 6 | 9.2 | 5.7 | 79 | 988.7 |
| 06-13-2009 | 1 | 7 | 7.5 | 5.1 | 85 | 990.5 |
| 06-14-2009 | 0 | 4 | 10.5 | 7.2 | 80 | 990.5 |
| 06-15-2009 | 3 | 6 | 13.8 | 7.6 | 69 | 987.3 |
| 06-16-2009 | 4 | 6 | 10.8 | 7.9 | 83 | 987.1 |
| 06-17-2009 | 2 | 5 | 9.7 | 4.3 | 71 | 993.1 |
| 06-18-2009 | 3 | 9 | 11.1 | 8.2 | 83 | 990.5 |
| 06-19-2009 | 0 | 8 | 15.2 | 5.4 | 55 | 990.4 |
| 06-20-2009 | 1 | 8 | 14.0 | 4.5 | 56 | 991.2 |
| 06-21-2009 | 3 | 5 | 15.9 | 6.9 | 58 | 989.0 |
| 06-22-2009 | 0 | 10 | 15.4 | 9.0 | 67 | 987.5 |
| 06-23-2009 | 2 | 6 | 17.3 | 9.4 | 63 | 982.8 |
| 06-24-2009 | 0 | 6 | 16.0 | 8.4 | 64 | 982.5 |
| 06-25-2009 | 0 | 5 | 14.2 | 11.2 | 83 | 985.3 |
| 06-26-2009 | 0 | 5 | 11.9 | 9.1 | 84 | 991.0 |
| 06-27-2009 | 1 | 6 | 12.7 | 9.0 | 79 | 989.6 |
| 06-28-2009 | 4 | 6 | 11.1 | 8.3 | 83 | 987.9 |
| 06-29-2009 | 2 | 7 | 12.4 | 7.9 | 77 | 989.2 |
| 06-30-2009 | 2 | 12 | 18.7 | 8.6 | 54 | 989.4 |
| 07-01-2009 | 0 | 6 | 13.8 | 6.4 | 62 | 989.7 |
| 07-03-2009 | 1 | 5 | 14.8 | 6.7 | 63 | 984.7 |
| 07-04-2009 | 2 | 5 | 20.8 | 11.0 | 55 | 984.4 |
| 07-05-2009 | 0 | 8 | 21.6 | 13.4 | 61 | 985.8 |
| 07-06-2009 | 2 | 10 | 21.7 | 12.9 | 60 | 989.2 |
| 07-07-2009 | 0 | 8 | 22.8 | 14.7 | 63 | 990.0 |
| 07-08-2009 | 1 | 3 | 18.9 | 15.2 | 80 | 986.5 |
| 07-09-2009 | 2 | 8 | 20.9 | 14.0 | 68 | 984.9 |
| 07-10-2009 | 4 | 13 | 23.0 | 15.5 | 64 | 982.5 |
| 07-11-2009 | 1 | 5 | 19.9 | 12.5 | 65 | 985.3 |
| 07-12-2009 | 1 | 8 | 20.1 | 15.2 | 74 | 977.9 |
| 07-13-2009 | 1 | 13 | 20.0 | 11.9 | 62 | 981.2 |
| 07-14-2009 | 1 | 7 | 21.3 | 13.4 | 62 | 982.2 |
| 07-15-2009 | 2 | 9 | 21.7 | 14.8 | 67 | 981.0 |
| 07-16-2009 | 4 | 11 | 21.4 | 15.2 | 70 | 980.0 |
| 07-17-2009 | 4 | 3 | 20.0 | 15.1 | 75 | 979.6 |
| 07-18-2009 | 2 | 5 | 20.2 | 15.7 | 76 | 981.6 |
| 07-19-2009 | 1 | 5 | 20.4 | 15.5 | 75 | 982.4 |
| 07-20-2009 | 5 | 8 | 20.3 | 16.4 | 80 | 983.8 |
| 07-21-2009 | 5 | 10 | 19.7 | 14.5 | 74 | 979.5 |
| 07-22-2009 | 2 | 8 | 11.9 | 8.9 | 81 | 982.7 |
| 07-23-2009 | 2 | 7 | 13.4 | 9.6 | 79 | 986.0 |
| 07-24-2009 | 1 | 8 | 15.1 | 10.8 | 77 | 989.5 |
| 07-25-2009 | 2 | 5 | 16.0 | 14.3 | 90 | 989.5 |
| 07-26-2009 | 1 | 5 | 18.3 | 13.9 | 78 | 986.3 |
| 07-27-2009 | 4 | 11 | 19.7 | 12.9 | 67 | 980.2 |
| 07-28-2009 | 2 | 21 | 20.6 | 13.1 | 65 | 979.3 |
| 07-29-2009 | 2 | 5 | 18.8 | 14.3 | 76 | 981.1 |
| 07-30-2009 | 5 | 15 | 18.0 | 15.3 | 86 | 982.4 |
| 07-31-2009 | 6 | 7 | 16.5 | 11.8 | 76 | 986.9 |
| 08-01-2009 | 2 | 14 | 18.4 | 12.6 | 72 | 985.7 |
| 08-03-2009 | 2 | 10 | 18.8 | 15.6 | 83 | 983.3 |
| 08-04-2009 | 1 | 14 | 18.3 | 14.7 | 80 | 982.5 |
| 08-05-2009 | 1 | 11 | 16.0 | 13.8 | 87 | 982.3 |
| 08-06-2009 | 4 | 12 | 14.6 | 12.1 | 86 | 989.8 |
| 08-07-2009 | 0 | 7 | 16.9 | 11.2 | 72 | 991.1 |
| 08-08-2009 | 1 | 14 | 21.8 | 14.3 | 64 | 983.9 |
| 08-09-2009 | 0 | 11 | 19.4 | 14.6 | 74 | 981.1 |
| 08-10-2009 | 1 | 12 | 20.1 | 12.7 | 64 | 980.8 |
| 08-11-2009 | 4 | 18 | 16.4 | 11.2 | 73 | 982.3 |
| 08-12-2009 | 2 | 8 | 13.1 | 9.5 | 79 | 990.2 |
| 08-13-2009 | 1 | 15 | 12.0 | 9.2 | 84 | 997.7 |
| 08-14-2009 | 0 | 9 | 13.3 | 7.5 | 71 | 1002.9 |
| 08-15-2009 | 2 | 8 | 14.1 | 7.2 | 66 | 1001.2 |
| 08-16-2009 | 1 | 14 | 15.5 | 9.9 | 72 | 997.1 |
| 08-17-2009 | 3 | 14 | 17.0 | 9.9 | 66 | 995.5 |
| 08-18-2009 | 1 | 6 | 17.4 | 9.8 | 65 | 992.4 |
| 08-19-2009 | 3 | 8 | 16.8 | 10.6 | 70 | 989.6 |
| 08-20-2009 | 1 | 7 | 17.1 | 10.4 | 68 | 988.8 |
| 08-21-2009 | 3 | 10 | 15.7 | 12.7 | 83 | 989.2 |
| 08-22-2009 | 1 | 8 | 16.2 | 11.3 | 74 | 991.0 |
| 08-23-2009 | 0 | 15 | 15.7 | 11.2 | 76 | 995.6 |
| 08-24-2009 | 1 | 14 | 16.2 | 10.3 | 72 | 996.1 |
| 08-25-2009 | 2 | 10 | 16.6 | 11.6 | 75 | 995.6 |
| 08-26-2009 | 3 | 10 | 18.1 | 11.0 | 67 | 992.1 |
| 08-27-2009 | 1 | 9 | 18.5 | 11.7 | 68 | 988.2 |
| 08-28-2009 | 4 | 23 | 18.2 | 12.9 | 72 | 986.1 |
| 08-29-2009 | 0 | 16 | 16.9 | 13.0 | 80 | 981.4 |
| 08-30-2009 | 1 | 6 | 17.4 | 13.0 | 77 | 986.5 |
| 08-31-2009 | 3 | 10 | 18.9 | 12.9 | 70 | 985.1 |
| 09-01-2009 | 2 | 7 | 8.2 | 6.8 | 91 | 991.5 |
| 09-03-2009 | 5 | 9 | 10.4 | 5.0 | 70 | 999.3 |
| 09-04-2009 | 1 | 13 | 9.9 | 1.8 | 60 | 998.3 |
| 09-05-2009 | 1 | 8 | 10.1 | 2.5 | 62 | 997.8 |
| 09-06-2009 | 0 | 10 | 10.0 | 3.1 | 65 | 996.9 |
| 09-07-2009 | 3 | 7 | 10.1 | 4.4 | 71 | 997.6 |
| 09-08-2009 | 3 | 13 | 11.4 | 5.6 | 70 | 999.7 |
| 09-09-2009 | 0 | 6 | 13.0 | 6.2 | 67 | 1002.5 |
| 09-10-2009 | 2 | 8 | 13.7 | 6.8 | 66 | 997.5 |
| 09-11-2009 | 1 | 10 | 13.8 | 8.2 | 71 | 995.5 |
| 09-12-2009 | 1 | 13 | 15.5 | 8.0 | 63 | 995.7 |
| 09-13-2009 | 0 | 9 | 14.7 | 7.5 | 65 | 997.5 |
| 09-14-2009 | 0 | 16 | 12.4 | 5.4 | 64 | 992.5 |
| 09-15-2009 | 0 | 4 | 8.6 | 2.0 | 66 | 990.1 |
| 09-16-2009 | 0 | 10 | 11.9 | 6.3 | 72 | 983.5 |
| 09-17-2009 | 1 | 11 | 6.9 | 3.9 | 81 | 991.2 |
| 09-18-2009 | 3 | 20 | 6.0 | -0.9 | 63 | 1008.5 |
| 09-19-2009 | 1 | 5 | 7.8 | 0.2 | 62 | 1003.3 |
| 09-20-2009 | 1 | 6 | 13.3 | 5.2 | 61 | 992.3 |
| 09-21-2009 | 2 | 5 | 15.1 | 6.9 | 60 | 987.8 |
| 09-22-2009 | 1 | 14 | 11.5 | 9.0 | 84 | 984.9 |
| 09-23-2009 | 3 | 8 | 8.1 | 0.7 | 61 | 996.7 |
| 09-24-2009 | 2 | 12 | 11.8 | 4.3 | 60 | 997.2 |
| 09-25-2009 | 2 | 9 | 14.5 | 3.8 | 49 | 992.9 |
| 09-26-2009 | 1 | 4 | 17.0 | 5.2 | 47 | 989.6 |
| 09-27-2009 | 1 | 3 | 14.5 | 5.2 | 56 | 991.9 |
| 09-28-2009 | 1 | 13 | 12.1 | 5.6 | 65 | 990.1 |
| 09-29-2009 | 1 | 9 | 9.9 | 3.6 | 67 | 999.4 |
| 09-30-2009 | 1 | 15 | 10.9 | 4.0 | 66 | 999.7 |
| 10-01-2009 | 0 | 12 | 11.7 | 3.5 | 62 | 997.3 |
| 10-03-2009 | 0 | 6 | 13.6 | 8.7 | 74 | 994.8 |
| 10-04-2009 | 0 | 8 | 8.0 | 5.9 | 87 | 1003.1 |
| 10-05-2009 | 0 | 13 | 9.4 | 3.2 | 68 | 999.3 |
| 10-06-2009 | 3 | 16 | 5.1 | 0.6 | 74 | 1005.3 |
| 10-07-2009 | 3 | 5 | 5.9 | 0.4 | 71 | 998.2 |
| 10-08-2009 | 1 | 9 | 7.3 | 0.9 | 68 | 993.5 |
| 10-09-2009 | 1 | 10 | 8.6 | 1.2 | 64 | 997.2 |
| 10-10-2009 | 0 | 7 | 8.6 | 1.2 | 64 | 1000.5 |
| 10-11-2009 | 2 | 6 | 10.1 | 3.3 | 64 | 996.2 |
| 10-12-2009 | 0 | 5 | 3.9 | 0.3 | 77 | 1006.1 |
| 10-13-2009 | 2 | 11 | 0.7 | -4.4 | 70 | 1012.9 |
| 10-14-2009 | 1 | 7 | 2.5 | -5.8 | 55 | 1010.7 |
| 10-15-2009 | 2 | 6 | 8.7 | 1.9 | 63 | 996.5 |
| 10-16-2009 | 0 | 8 | 2.5 | 0.2 | 84 | 997.7 |
| 10-17-2009 | 0 | 2 | 2.2 | -2.4 | 70 | 1001.2 |
| 10-18-2009 | 0 | 3 | 5.8 | 1.7 | 74 | 996.4 |
| 10-19-2009 | 2 | 7 | 3.3 | -1.1 | 73 | 998.6 |
| 10-20-2009 | 0 | 3 | 6.5 | 4.5 | 87 | 995.1 |
| 10-21-2009 | 0 | 8 | 9.1 | 3.7 | 68 | 989.0 |
| 10-22-2009 | 1 | 5 | 1.1 | -2.1 | 79 | 992.4 |
| 10-23-2009 | 0 | 5 | -4.3 | -9.7 | 63 | 1004.3 |
| 10-24-2009 | 0 | 4 | -2.9 | -6.6 | 73 | 1001.3 |
| 10-25-2009 | 1 | 4 | -0.3 | -2.2 | 86 | 989.9 |
| 10-26-2009 | 0 | 7 | -5.2 | -8.3 | 76 | 995.4 |
| 10-27-2009 | 0 | 8 | -10.0 | -12.9 | 77 | 1003.9 |
| 10-28-2009 | 3 | 7 | -8.7 | -11.7 | 77 | 1002.7 |
| 10-29-2009 | 0 | 12 | -9.3 | -11.8 | 81 | 1006.4 |
| 10-30-2009 | 1 | 8 | -5.8 | -8.0 | 82 | 1015.1 |
| 10-31-2009 | 0 | 3 | -4.8 | -7.9 | 77 | 1012.5 |
| 11-01-2009 | 0 | 6 | -0.8 | -6.4 | 67 | 1001.5 |
| 11-03-2009 | 1 | 10 | 1.8 | 1.5 | 98 | 995.7 |
| 11-04-2009 | 0 | 9 | 1.0 | 0.2 | 93 | 985.8 |
| 11-05-2009 | 1 | 8 | -7.2 | -9.5 | 81 | 994.8 |
| 11-06-2009 | 1 | 6 | -15.9 | -19.0 | 75 | 1009.3 |
| 11-07-2009 | 0 | 5 | -16.7 | -19.9 | 73 | 1010.2 |
| 11-08-2009 | 0 | 7 | -17.2 | -20.0 | 76 | 1012.5 |
| 11-09-2009 | 2 | 7 | -17.3 | -21.1 | 70 | 1015.2 |
| 11-10-2009 | 4 | 9 | -20.9 | -24.7 | 69 | 1016.8 |
| 11-11-2009 | 2 | 7 | -16.4 | -19.8 | 72 | 1012.6 |
| 11-12-2009 | 1 | 7 | -15.8 | -19.6 | 70 | 1013.1 |
| 11-13-2009 | 0 | 0 | -17.2 | -20.8 | 71 | 1020.2 |
| 11-14-2009 | 0 | 8 | -19.7 | -23.3 | 70 | 1025.9 |
| 11-15-2009 | 1 | 9 | -12.6 | -16.5 | 69 | 1021.5 |
| 11-16-2009 | 0 | 3 | -6.6 | -10.4 | 71 | 1014.2 |
| 11-17-2009 | 1 | 5 | -9.2 | -12.9 | 72 | 1006.6 |
| 11-18-2009 | 0 | 8 | -7.9 | -9.5 | 86 | 1012.8 |
| 11-19-2009 | 0 | 17 | -5.8 | -7.5 | 87 | 1012.9 |
| 11-20-2009 | 1 | 4 | -5.5 | -11.2 | 60 | 1002.7 |
| 11-21-2009 | 0 | 6 | -6.8 | -11.1 | 69 | 994.5 |
| 11-22-2009 | 0 | 11 | -0.7 | -2.5 | 86 | 986.4 |
| 11-23-2009 | 0 | 5 | 0.6 | -2.4 | 79 | 987.7 |
| 11-24-2009 | 3 | 5 | -7.4 | -10.8 | 74 | 1001.2 |
| 11-25-2009 | 1 | 11 | -4.4 | -5.9 | 88 | 1002.6 |
| 11-26-2009 | 0 | 5 | 0.3 | -1.9 | 84 | 1001.9 |
| 11-27-2009 | 0 | 4 | 0.6 | -2.8 | 77 | 1002.8 |
| 11-28-2009 | 1 | 3 | -3.8 | -6.8 | 77 | 1001.1 |
| 11-29-2009 | 0 | 4 | -2.8 | -4.7 | 85 | 1000.8 |
| 11-30-2009 | 0 | 10 | 0.2 | -2.2 | 83 | 1005.3 |
| 12-01-2009 | 0 | 9 | -0.9 | -2.5 | 88 | 1000.9 |
| 12-03-2009 | 2 | 3 | -14.4 | -22.7 | 49 | 1006.7 |
| 12-04-2009 | 1 | 4 | -4.2 | -11.7 | 54 | 990.1 |
| 12-05-2009 | 0 | 3 | -3.5 | -4.9 | 89 | 989.2 |
| 12-06-2009 | 1 | 7 | -10.6 | -13.2 | 79 | 998.5 |
| 12-07-2009 | 0 | 5 | -20.7 | -23.4 | 77 | 1003.0 |
| 12-08-2009 | 1 | 5 | -30.2 | -33.3 | 72 | 1009.0 |
| 12-09-2009 | 1 | 7 | -27.1 | -30.4 | 71 | 1007.3 |
| 12-10-2009 | 2 | 4 | -18.6 | -22.2 | 70 | 1000.9 |
| 12-11-2009 | 1 | 4 | -19.3 | -23.0 | 69 | 1006.8 |
| 12-12-2009 | 2 | 1 | -16.0 | -20.5 | 65 | 1004.7 |
| 12-13-2009 | 1 | 2 | -19.8 | -24.2 | 65 | 1011.3 |
| 12-14-2009 | 0 | 7 | -19.1 | -23.2 | 67 | 1011.7 |
| 12-15-2009 | 0 | 11 | -14.9 | -20.5 | 59 | 1006.2 |
| 12-16-2009 | 3 | 4 | -12.1 | -18.1 | 57 | 1001.1 |
| 12-17-2009 | 0 | 5 | -13.6 | -17.4 | 71 | 1009.9 |
| 12-18-2009 | 0 | 4 | -16.2 | -18.7 | 79 | 1009.5 |
| 12-19-2009 | 0 | 3 | -11.8 | -13.7 | 83 | 1000.8 |
| 12-20-2009 | 0 | 7 | -7.2 | -8.6 | 88 | 992.6 |
| 12-21-2009 | 0 | 4 | -19.8 | -22.3 | 77 | 1000.8 |
| 12-22-2009 | 5 | 12 | -33.0 | -36.0 | 72 | 1009.8 |
| 12-23-2009 | 0 | 7 | -35.7 | -38.9 | 69 | 1025.3 |
| 12-24-2009 | 0 | 7 | -28.3 | -31.3 | 73 | 1007.9 |
| 12-25-2009 | 1 | 5 | -22.1 | -25.4 | 72 | 1008.8 |
| 12-26-2009 | 0 | 4 | -10.9 | -16.0 | 63 | 994.3 |
| 12-27-2009 | 1 | 5 | -14.3 | -17.1 | 77 | 994.7 |
| 12-28-2009 | 0 | 9 | -27.8 | -30.4 | 76 | 1009.4 |
| 12-29-2009 | 1 | 6 | -14.0 | -17.9 | 70 | 997.7 |
| 12-30-2009 | 0 | 8 | -7.9 | -10.7 | 77 | 990.5 |
| 12-31-2009 | 0 | 6 | -30.3 | -33.8 | 68 | 1002.2 |
| 01-01-2010 | 0 | 11 | -26.8 | -30.2 | 70 | 1000.6 |
| 01-02-2010 | 0 | 19 | -30.5 | -34.7 | 63 | 1009.7 |
| 01-03-2010 | 0 | 11 | -25.3 | -28.8 | 69 | 1001.8 |
| 01-04-2010 | 0 | 8 | -21.2 | -25.6 | 65 | 1002.7 |
| 01-05-2010 | 0 | 11 | -21.0 | -27.0 | 56 | 999.8 |
| 01-06-2010 | 0 | 11 | -30.4 | -34.5 | 64 | 999.4 |
| 01-07-2010 | 2 | 15 | -34.7 | -38.7 | 63 | 1005.4 |
| 01-08-2010 | 0 | 5 | -37.0 | -41.0 | 64 | 1019.9 |
| 01-09-2010 | 0 | 19 | -33.7 | -38.0 | 62 | 1024.3 |
| 01-10-2010 | 1 | 9 | -25.3 | -29.7 | 63 | 1020.5 |
| 01-11-2010 | 1 | 11 | -10.6 | -14.6 | 70 | 1001.5 |
| 01-12-2010 | 2 | 8 | -14.6 | -17.0 | 79 | 995.4 |
| 01-13-2010 | 0 | 8 | -20.5 | -23.4 | 75 | 997.4 |
| 01-14-2010 | 0 | 8 | -9.5 | -12.4 | 77 | 987.3 |
| 01-15-2010 | 0 | 10 | -15.7 | -19.3 | 71 | 994.5 |
| 01-16-2010 | 1 | 8 | -24.8 | -28.0 | 72 | 1005.6 |
| 01-17-2010 | 2 | 7 | -31.5 | -34.5 | 71 | 1008.6 |
| 01-18-2010 | 1 | 11 | -36.1 | -39.6 | 67 | 1028.3 |
| 01-19-2010 | 1 | 12 | -35.0 | -38.9 | 65 | 1042.6 |
| 01-20-2010 | 1 | 9 | -29.9 | -33.3 | 70 | 1037.4 |
| 01-21-2010 | 1 | 15 | -27.7 | -31.1 | 70 | 1027.0 |
| 01-22-2010 | 0 | 15 | -26.1 | -29.5 | 71 | 1014.5 |
| 01-23-2010 | 1 | 3 | -26.5 | -30.0 | 70 | 1016.1 |
| 01-24-2010 | 0 | 10 | -24.8 | -28.2 | 71 | 1008.2 |
| 01-25-2010 | 1 | 16 | -17.7 | -21.7 | 68 | 1001.8 |
| 01-26-2010 | 0 | 5 | -26.4 | -30.2 | 67 | 1015.7 |
| 01-27-2010 | 3 | 15 | -32.7 | -37.0 | 62 | 1021.7 |
| 01-28-2010 | 1 | 8 | -29.1 | -35.5 | 51 | 1018.9 |
| 01-29-2010 | 2 | 15 | -25.7 | -32.5 | 50 | 1011.2 |
| 01-30-2010 | 0 | 4 | -21.8 | -29.8 | 45 | 1011.3 |
| 01-31-2010 | 0 | 2 | -23.5 | -29.1 | 57 | 1014.5 |
| 02-01-2010 | 3 | 18 | -26.8 | -30.5 | 69 | 1016.0 |
| 02-02-2010 | 2 | 13 | -25.9 | -30.0 | 66 | 1011.0 |
| 02-03-2010 | 0 | 17 | -24.3 | -29.3 | 61 | 1005.4 |
| 02-04-2010 | 1 | 11 | -25.7 | -31.2 | 57 | 999.0 |
| 02-05-2010 | 0 | 12 | -28.7 | -32.5 | 67 | 998.7 |
| 02-06-2010 | 0 | 12 | -30.4 | -33.8 | 70 | 1004.4 |
| 02-07-2010 | 0 | 8 | -31.9 | -35.6 | 66 | 1015.6 |
| 02-08-2010 | 0 | 13 | -31.8 | -35.8 | 65 | 1018.5 |
| 02-09-2010 | 0 | 16 | -32.2 | -36.0 | 66 | 1024.1 |
| 02-10-2010 | 0 | 8 | -30.1 | -34.6 | 62 | 1026.3 |
| 02-11-2010 | 0 | 11 | -29.6 | -34.1 | 62 | 1023.6 |
| 02-12-2010 | 1 | 12 | -29.5 | -34.2 | 62 | 1022.6 |
| 02-13-2010 | 2 | 4 | -27.0 | -31.6 | 62 | 1015.0 |
| 02-14-2010 | 1 | 11 | -15.8 | -20.3 | 65 | 1005.2 |
| 02-15-2010 | 1 | 11 | -13.7 | -18.1 | 66 | 1010.3 |
| 02-16-2010 | 0 | 17 | -10.9 | -17.9 | 53 | 1007.9 |
| 02-17-2010 | 0 | 18 | -8.2 | -16.9 | 46 | 996.3 |
| 02-18-2010 | 0 | 22 | -3.7 | -7.1 | 75 | 979.7 |
| 02-19-2010 | 0 | 12 | -19.7 | -24.6 | 63 | 998.2 |
| 02-20-2010 | 0 | 17 | -15.6 | -21.2 | 59 | 994.7 |
| 02-21-2010 | 1 | 6 | -15.1 | -19.9 | 63 | 987.7 |
| 02-22-2010 | 0 | 17 | -27.5 | -32.7 | 59 | 1000.9 |
| 02-23-2010 | 0 | 7 | -29.7 | -34.4 | 60 | 1007.3 |
| 02-24-2010 | 0 | 13 | -29.4 | -34.3 | 60 | 1006.0 |
| 02-25-2010 | 1 | 11 | -25.1 | -31.0 | 55 | 1003.2 |
| 02-26-2010 | 0 | 18 | -21.5 | -29.5 | 47 | 1002.8 |
| 02-27-2010 | 0 | 14 | -16.8 | -21.2 | 66 | 995.3 |
| 02-28-2010 | 1 | 12 | -15.9 | -20.3 | 66 | 999.5 |
| 03-01-2010 | 0 | 16 | -13.8 | -18.1 | 67 | 995.5 |
| 03-02-2010 | 0 | 15 | -22.7 | -26.9 | 65 | 1014.6 |
| 03-03-2010 | 0 | 17 | -21.5 | -26.2 | 63 | 1012.6 |
| 03-04-2010 | 0 | 15 | -16.5 | -20.5 | 69 | 1004.0 |
| 03-05-2010 | 0 | 7 | -17.9 | -21.6 | 71 | 1012.5 |
| 03-06-2010 | 0 | 9 | -15.8 | -21.5 | 60 | 1014.0 |
| 03-07-2010 | 3 | 8 | -13.6 | -19.4 | 59 | 1011.3 |
| 03-08-2010 | 0 | 20 | -8.6 | -15.2 | 56 | 1004.7 |
| 03-09-2010 | 0 | 14 | -1.8 | -6.7 | 68 | 992.5 |
| 03-10-2010 | 0 | 12 | -4.6 | -7.6 | 77 | 989.7 |
| 03-11-2010 | 0 | 9 | -9.2 | -13.3 | 70 | 991.4 |
| 03-12-2010 | 0 | 15 | -6.6 | -10.0 | 74 | 988.8 |
| 03-13-2010 | 0 | 8 | -8.8 | -13.5 | 65 | 1009.7 |
| 03-14-2010 | 0 | 13 | -4.9 | -13.9 | 45 | 1004.4 |
| 03-15-2010 | 0 | 23 | -3.0 | -7.6 | 68 | 990.1 |
| 03-16-2010 | 0 | 19 | 0.3 | -3.9 | 71 | 994.4 |
| 03-17-2010 | 1 | 15 | 0.1 | -3.2 | 78 | 981.7 |
| 03-18-2010 | 0 | 10 | -3.2 | -7.2 | 72 | 990.6 |
| 03-19-2010 | 0 | 18 | -4.4 | -14.7 | 41 | 998.8 |
| 03-20-2010 | 0 | 10 | -7.4 | -11.4 | 70 | 994.4 |
| 03-21-2010 | 0 | 11 | -9.9 | -15.0 | 63 | 1005.5 |
| 03-22-2010 | 0 | 19 | -9.4 | -14.9 | 63 | 1003.7 |
| 03-23-2010 | 0 | 12 | -6.9 | -13.0 | 61 | 1006.1 |
| 03-24-2010 | 1 | 9 | 0.9 | -5.8 | 59 | 1000.6 |
| 03-25-2010 | 1 | 10 | -2.5 | -9.4 | 58 | 1004.2 |
| 03-26-2010 | 0 | 6 | 0.9 | -6.4 | 57 | 991.0 |
| 03-27-2010 | 0 | 12 | -4.3 | -10.4 | 60 | 992.1 |
| 03-28-2010 | 0 | 8 | -6.5 | -10.6 | 69 | 1001.9 |
| 03-29-2010 | 0 | 7 | -5.6 | -10.7 | 64 | 1001.8 |
| 03-30-2010 | 0 | 12 | -6.8 | -12.5 | 61 | 1012.7 |
| 03-31-2010 | 0 | 8 | -3.0 | -9.0 | 60 | 1006.5 |
| 04-01-2010 | 1 | 11 | 0.4 | -8.5 | 51 | 998.8 |
| 04-02-2010 | 0 | 9 | 0.4 | -5.1 | 65 | 998.1 |
| 04-03-2010 | 0 | 8 | -0.2 | -5.0 | 69 | 1001.8 |
| 04-04-2010 | 1 | 4 | 0.9 | -4.7 | 66 | 1003.5 |
| 04-05-2010 | 2 | 9 | 1.5 | -2.9 | 72 | 1000.5 |
| 04-06-2010 | 2 | 10 | -4.5 | -12.4 | 52 | 1001.5 |
| 04-07-2010 | 1 | 12 | -7.6 | -14.1 | 56 | 1006.0 |
| 04-08-2010 | 0 | 3 | -5.6 | -11.4 | 62 | 1008.7 |
| 04-09-2010 | 0 | 9 | -7.3 | -17.5 | 44 | 1014.0 |
| 04-10-2010 | 1 | 5 | -7.6 | -17.4 | 44 | 1014.2 |
| 04-11-2010 | 0 | 4 | -6.5 | -15.9 | 45 | 1012.8 |
| 04-12-2010 | 0 | 14 | -2.6 | -12.5 | 46 | 1003.7 |
| 04-13-2010 | 1 | 5 | 1.6 | -4.3 | 67 | 996.5 |
| 04-14-2010 | 0 | 9 | 1.7 | 0.2 | 89 | 996.8 |
| 04-15-2010 | 0 | 4 | 2.6 | -0.4 | 81 | 989.3 |
| 04-16-2010 | 0 | 13 | 2.3 | -1.2 | 77 | 990.8 |
| 04-17-2010 | 1 | 4 | 3.2 | -2.3 | 68 | 1003.7 |
| 04-18-2010 | 0 | 6 | 4.4 | -1.5 | 67 | 1005.0 |
| 04-19-2010 | 0 | 10 | 5.0 | 1.3 | 77 | 1007.4 |
| 04-20-2010 | 0 | 15 | 8.9 | 1.5 | 61 | 1004.5 |
| 04-21-2010 | 0 | 16 | 10.3 | 1.0 | 54 | 996.8 |
| 04-22-2010 | 0 | 10 | 7.3 | -2.7 | 50 | 1003.2 |
| 04-23-2010 | 0 | 8 | 4.3 | -4.5 | 54 | 1015.7 |
| 04-24-2010 | 0 | 5 | 7.7 | -2.8 | 50 | 1015.2 |
| 04-25-2010 | 0 | 9 | 12.7 | 0.4 | 45 | 1005.4 |
| 04-26-2010 | 1 | 3 | 14.9 | -0.1 | 42 | 997.3 |
| 04-27-2010 | 0 | 7 | 19.9 | 3.7 | 36 | 987.4 |
| 04-28-2010 | 0 | 11 | 13.7 | 3.0 | 51 | 996.3 |
| 04-29-2010 | 0 | 11 | 13.1 | 4.8 | 61 | 990.0 |
| 04-30-2010 | 0 | 6 | 6.0 | -2.8 | 55 | 994.7 |
| 05-01-2010 | 0 | 8 | 12.6 | 0.0 | 43 | 993.1 |
| 05-02-2010 | 0 | 3 | 9.6 | -0.2 | 51 | 993.2 |
| 05-03-2010 | 0 | 8 | 11.2 | 1.3 | 53 | 991.5 |
| 05-04-2010 | 0 | 9 | 4.7 | 1.5 | 80 | 991.6 |
| 05-05-2010 | 0 | 7 | 7.0 | 2.0 | 72 | 994.9 |
| 05-06-2010 | 0 | 6 | 7.9 | -1.4 | 54 | 998.0 |
| 05-07-2010 | 2 | 6 | 9.0 | -1.8 | 48 | 999.6 |
| 05-08-2010 | 2 | 4 | 12.3 | 1.0 | 55 | 991.0 |
| 05-09-2010 | 4 | 4 | 7.5 | -3.0 | 50 | 997.5 |
| 05-10-2010 | 0 | 9 | 6.0 | -5.9 | 43 | 998.1 |
| 05-11-2010 | 0 | 10 | 2.0 | -7.4 | 50 | 999.7 |
| 05-12-2010 | 1 | 9 | 2.9 | 0.6 | 85 | 994.5 |
| 05-13-2010 | 1 | 8 | 7.6 | -3.7 | 46 | 995.8 |
| 05-14-2010 | 0 | 8 | 10.6 | -0.5 | 47 | 1001.6 |
| 05-15-2010 | 1 | 8 | 12.6 | 0.7 | 47 | 999.4 |
| 05-16-2010 | 1 | 2 | 17.2 | 3.7 | 42 | 990.2 |
| 05-17-2010 | 1 | 8 | 11.6 | 6.0 | 69 | 987.7 |
| 05-18-2010 | 0 | 7 | 5.3 | -1.9 | 60 | 993.4 |
| 05-19-2010 | 1 | 7 | 0.6 | -4.1 | 71 | 995.7 |
| 05-20-2010 | 0 | 12 | 3.8 | -5.4 | 52 | 992.8 |
| 05-21-2010 | 1 | 9 | 4.7 | -4.2 | 54 | 989.3 |
| 05-22-2010 | 1 | 5 | 6.6 | -3.1 | 51 | 991.1 |
| 05-23-2010 | 0 | 8 | 9.8 | 2.1 | 60 | 985.4 |
| 05-24-2010 | 0 | 9 | 11.5 | 2.2 | 55 | 993.4 |
| 05-25-2010 | 0 | 7 | 12.8 | -0.0 | 44 | 995.6 |
| 05-26-2010 | 1 | 8 | 17.6 | 3.0 | 39 | 990.7 |
| 05-27-2010 | 0 | 6 | 21.5 | 7.3 | 43 | 986.4 |
| 05-28-2010 | 1 | 8 | 19.4 | 13.3 | 69 | 986.3 |
| 05-29-2010 | 0 | 3 | 19.6 | 9.1 | 54 | 989.9 |
| 05-30-2010 | 0 | 4 | 21.1 | 10.2 | 52 | 986.0 |
| 05-31-2010 | 0 | 0 | 13.9 | 6.9 | 64 | 990.5 |
| 06-01-2010 | 0 | 7 | 10.2 | 2.9 | 61 | 997.8 |
| 06-02-2010 | 1 | 7 | 17.0 | 3.8 | 44 | 995.8 |
| 06-03-2010 | 3 | 6 | 18.1 | 6.8 | 48 | 996.7 |
| 06-04-2010 | 0 | 6 | 17.2 | 11.2 | 70 | 992.1 |
| 06-05-2010 | 0 | 3 | 15.2 | 5.7 | 57 | 995.2 |
| 06-06-2010 | 1 | 5 | 15.2 | 1.2 | 41 | 997.2 |
| 06-07-2010 | 0 | 8 | 20.2 | 5.0 | 40 | 991.3 |
| 06-08-2010 | 1 | 12 | 23.7 | 8.8 | 40 | 985.3 |
| 06-09-2010 | 0 | 11 | 20.1 | 13.1 | 67 | 983.4 |
| 06-10-2010 | 0 | 8 | 16.6 | 6.7 | 55 | 988.5 |
| 06-11-2010 | 1 | 7 | 21.4 | 8.0 | 44 | 983.6 |
| 06-12-2010 | 0 | 3 | 16.5 | 10.0 | 66 | 985.1 |
| 06-13-2010 | 1 | 4 | 16.8 | 5.7 | 49 | 992.4 |
| 06-14-2010 | 0 | 3 | 21.5 | 10.6 | 52 | 987.6 |
| 06-15-2010 | 0 | 5 | 27.0 | 13.0 | 43 | 983.9 |
| 06-16-2010 | 1 | 14 | 22.4 | 13.1 | 56 | 986.9 |
| 06-17-2010 | 2 | 15 | 18.9 | 8.2 | 51 | 992.5 |
| 06-18-2010 | 1 | 3 | 19.9 | 10.1 | 55 | 989.2 |
| 06-19-2010 | 0 | 10 | 22.2 | 9.7 | 49 | 986.1 |
| 06-20-2010 | 1 | 6 | 27.0 | 15.2 | 50 | 981.1 |
| 06-21-2010 | 1 | 4 | 20.8 | 17.1 | 80 | 975.8 |
| 06-22-2010 | 2 | 10 | 11.9 | 7.7 | 76 | 985.3 |
| 06-23-2010 | 1 | 8 | 12.4 | 7.2 | 72 | 991.7 |
| 06-24-2010 | 0 | 8 | 14.5 | 6.8 | 62 | 990.3 |
| 06-25-2010 | 1 | 5 | 15.4 | 11.1 | 77 | 988.6 |
| 06-26-2010 | 0 | 6 | 17.0 | 10.1 | 66 | 991.4 |
| 06-27-2010 | 0 | 6 | 18.1 | 8.1 | 54 | 989.5 |
| 06-28-2010 | 0 | 7 | 14.8 | 11.8 | 83 | 989.0 |
| 06-29-2010 | 2 | 8 | 14.7 | 12.7 | 88 | 990.2 |
| 06-30-2010 | 0 | 11 | 16.0 | 14.2 | 88 | 987.5 |
| 07-01-2010 | 3 | 5 | 19.0 | 15.1 | 79 | 986.3 |
| 07-02-2010 | 0 | 7 | 17.7 | 14.0 | 79 | 988.3 |
| 07-03-2010 | 0 | 13 | 15.6 | 9.5 | 69 | 994.2 |
| 07-04-2010 | 0 | 7 | 19.2 | 9.6 | 54 | 994.2 |
| 07-05-2010 | 0 | 10 | 20.9 | 11.5 | 58 | 986.9 |
| 07-06-2010 | 0 | 6 | 21.2 | 13.8 | 65 | 981.3 |
| 07-07-2010 | 4 | 7 | 17.6 | 11.5 | 69 | 983.2 |
| 07-08-2010 | 1 | 3 | 15.7 | 8.5 | 65 | 987.2 |
| 07-09-2010 | 0 | 5 | 13.8 | 11.6 | 87 | 983.0 |
| 07-10-2010 | 2 | 4 | 14.1 | 11.1 | 83 | 981.5 |
| 07-11-2010 | 2 | 4 | 15.6 | 11.5 | 77 | 983.6 |
| 07-12-2010 | 1 | 11 | 16.3 | 11.4 | 74 | 984.1 |
| 07-13-2010 | 2 | 8 | 17.0 | 12.7 | 77 | 985.6 |
| 07-14-2010 | 4 | 6 | 21.0 | 13.1 | 64 | 987.6 |
| 07-15-2010 | 2 | 8 | 22.5 | 14.5 | 64 | 987.2 |
| 07-16-2010 | 1 | 12 | 23.6 | 16.0 | 65 | 984.6 |
| 07-17-2010 | 1 | 4 | 23.9 | 18.5 | 73 | 978.9 |
| 07-18-2010 | 2 | 6 | 21.5 | 18.5 | 83 | 977.5 |
| 07-19-2010 | 0 | 7 | 16.9 | 13.4 | 81 | 983.7 |
| 07-20-2010 | 1 | 10 | 16.8 | 11.4 | 72 | 986.8 |
| 07-21-2010 | 0 | 8 | 14.8 | 10.9 | 78 | 988.2 |
| 07-22-2010 | 4 | 13 | 15.6 | 11.4 | 77 | 988.5 |
| 07-23-2010 | 0 | 6 | 13.9 | 12.6 | 92 | 991.6 |
| 07-24-2010 | 2 | 8 | 17.0 | 12.9 | 78 | 994.2 |
| 07-25-2010 | 1 | 7 | 21.3 | 15.0 | 69 | 986.9 |
| 07-26-2010 | 0 | 10 | 20.9 | 14.5 | 69 | 982.9 |
| 07-27-2010 | 0 | 7 | 11.1 | 9.8 | 91 | 988.9 |
| 07-28-2010 | 1 | 6 | 10.8 | 8.2 | 84 | 997.9 |
| 07-29-2010 | 1 | 5 | 11.9 | 7.9 | 77 | 999.2 |
| 07-30-2010 | 0 | 9 | 15.1 | 8.1 | 66 | 996.2 |
| 07-31-2010 | 0 | 0 | 17.3 | 11.4 | 70 | 993.0 |
| 08-01-2010 | 2 | 11 | 15.8 | 8.2 | 62 | 994.2 |
| 08-02-2010 | 0 | 8 | 13.5 | 7.8 | 70 | 998.4 |
| 08-03-2010 | 0 | 7 | 17.2 | 8.3 | 57 | 998.0 |
| 08-04-2010 | 1 | 13 | 19.7 | 11.9 | 63 | 993.3 |
| 08-05-2010 | 1 | 14 | 20.5 | 13.9 | 69 | 994.5 |
| 08-06-2010 | 1 | 6 | 17.5 | 9.3 | 62 | 997.6 |
| 08-07-2010 | 1 | 10 | 21.0 | 14.4 | 67 | 992.7 |
| 08-08-2010 | 0 | 3 | 21.1 | 16.9 | 79 | 980.9 |
| 08-09-2010 | 3 | 10 | 16.4 | 11.5 | 74 | 984.0 |
| 08-10-2010 | 0 | 6 | 14.7 | 9.1 | 70 | 988.7 |
| 08-11-2010 | 0 | 2 | 12.0 | 9.1 | 83 | 994.5 |
| 08-12-2010 | 4 | 9 | 14.4 | 7.1 | 65 | 999.1 |
| 08-13-2010 | 0 | 12 | 16.8 | 8.7 | 62 | 993.9 |
| 08-14-2010 | 0 | 5 | 19.0 | 12.3 | 69 | 989.3 |
| 08-15-2010 | 2 | 9 | 14.2 | 9.8 | 75 | 993.1 |
| 08-16-2010 | 2 | 15 | 12.8 | 7.3 | 70 | 996.6 |
| 08-17-2010 | 0 | 9 | 15.3 | 9.6 | 72 | 994.5 |
| 08-18-2010 | 0 | 8 | 18.1 | 9.1 | 60 | 984.3 |
| 08-19-2010 | 1 | 10 | 12.7 | 5.4 | 63 | 995.1 |
| 08-20-2010 | 0 | 14 | 18.8 | 8.8 | 55 | 992.8 |
| 08-21-2010 | 3 | 6 | 23.3 | 12.3 | 53 | 983.2 |
| 08-22-2010 | 0 | 9 | 26.5 | 12.5 | 44 | 976.2 |
| 08-23-2010 | 1 | 9 | 17.9 | 12.2 | 69 | 980.2 |
| 08-24-2010 | 1 | 9 | 12.4 | 5.0 | 63 | 997.7 |
| 08-25-2010 | 2 | 4 | 15.9 | 5.9 | 54 | 993.5 |
| 08-26-2010 | 0 | 9 | 18.9 | 8.4 | 55 | 991.8 |
| 08-27-2010 | 4 | 10 | 18.1 | 11.7 | 69 | 996.1 |
| 08-28-2010 | 0 | 6 | 18.5 | 11.8 | 69 | 992.7 |
| 08-29-2010 | 0 | 6 | 21.8 | 12.4 | 57 | 990.6 |
| 08-30-2010 | 1 | 8 | 20.1 | 11.6 | 60 | 993.1 |
| 08-31-2010 | 0 | 10 | 21.4 | 13.2 | 63 | 993.4 |
| 09-01-2010 | 1 | 11 | 21.8 | 15.6 | 70 | 986.9 |
| 09-02-2010 | 0 | 3 | 13.5 | 9.6 | 77 | 993.5 |
| 09-03-2010 | 2 | 8 | 8.0 | 2.4 | 69 | 1008.1 |
| 09-04-2010 | 0 | 2 | 9.3 | 2.4 | 64 | 1007.9 |
| 09-05-2010 | 2 | 6 | 15.6 | 3.5 | 46 | 1000.2 |
| 09-06-2010 | 2 | 10 | 21.2 | 6.9 | 41 | 992.1 |
| 09-07-2010 | 1 | 4 | 18.9 | 9.2 | 57 | 989.0 |
| 09-08-2010 | 3 | 9 | 22.1 | 5.8 | 38 | 975.0 |
| 09-09-2010 | 1 | 10 | 10.7 | 1.4 | 53 | 987.0 |
| 09-10-2010 | 2 | 5 | 10.5 | 1.4 | 54 | 989.7 |
| 09-11-2010 | 1 | 7 | 7.9 | 4.7 | 81 | 984.9 |
| 09-12-2010 | 1 | 7 | 3.6 | -2.5 | 65 | 999.8 |
| 09-13-2010 | 2 | 8 | 4.3 | -4.6 | 56 | 1002.3 |
| 09-14-2010 | 2 | 5 | 7.7 | 0.1 | 61 | 997.7 |
| 09-15-2010 | 0 | 8 | 4.9 | 1.9 | 81 | 996.2 |
| 09-16-2010 | 1 | 7 | 10.5 | -1.8 | 44 | 994.8 |
| 09-17-2010 | 0 | 4 | 10.0 | 5.9 | 77 | 994.2 |
| 09-18-2010 | 0 | 9 | 3.8 | -0.6 | 76 | 1004.7 |
| 09-19-2010 | 0 | 6 | 2.4 | -1.6 | 77 | 1011.0 |
| 09-20-2010 | 0 | 10 | 5.1 | -2.1 | 64 | 1011.6 |
| 09-21-2010 | 1 | 15 | 10.2 | 3.0 | 65 | 1006.2 |
| 09-22-2010 | 1 | 5 | 12.2 | 3.5 | 59 | 996.7 |
| 09-23-2010 | 0 | 10 | 9.7 | 3.3 | 65 | 997.4 |
| 09-24-2010 | 0 | 11 | 8.5 | 1.6 | 63 | 1003.1 |
| 09-25-2010 | 0 | 5 | 10.1 | 1.6 | 58 | 997.8 |
| 09-26-2010 | 2 | 11 | 10.5 | 3.6 | 65 | 989.9 |
| 09-27-2010 | 1 | 15 | 14.7 | 5.1 | 60 | 989.0 |
| 09-28-2010 | 0 | 8 | 11.6 | 7.0 | 76 | 992.4 |
| 09-29-2010 | 0 | 7 | 9.2 | 7.5 | 89 | 997.2 |
| 09-30-2010 | 0 | 11 | 13.1 | 5.5 | 62 | 996.5 |
| 10-01-2010 | 0 | 7 | 17.4 | 3.3 | 40 | 993.5 |
| 10-02-2010 | 1 | 8 | 19.1 | 4.0 | 37 | 990.1 |
| 10-03-2010 | 0 | 9 | 12.2 | 7.4 | 75 | 991.3 |
| 10-04-2010 | 0 | 8 | 5.3 | -1.4 | 62 | 995.8 |
| 10-05-2010 | 1 | 8 | 2.0 | -3.5 | 68 | 998.9 |
| 10-06-2010 | 2 | 8 | 2.9 | -3.6 | 64 | 995.8 |
| 10-07-2010 | 0 | 9 | 3.6 | -1.5 | 72 | 997.7 |
| 10-08-2010 | 3 | 5 | 3.8 | 2.9 | 93 | 996.6 |
| 10-09-2010 | 0 | 1 | 6.1 | 4.7 | 90 | 995.5 |
| 10-10-2010 | 1 | 10 | 7.1 | 3.7 | 79 | 995.2 |
| 10-11-2010 | 3 | 11 | 7.8 | 2.4 | 69 | 999.0 |
| 10-12-2010 | 2 | 10 | 7.0 | -0.2 | 63 | 1002.5 |
| 10-13-2010 | 2 | 12 | 7.7 | 0.5 | 62 | 996.5 |
| 10-14-2010 | 1 | 13 | 8.5 | 2.6 | 67 | 995.2 |
| 10-15-2010 | 0 | 12 | 9.1 | 2.8 | 67 | 991.8 |
| 10-16-2010 | 1 | 9 | 4.9 | 2.4 | 84 | 994.4 |
| 10-17-2010 | 0 | 7 | 5.1 | -1.7 | 62 | 999.7 |
| 10-18-2010 | 1 | 9 | 6.7 | 5.0 | 89 | 993.0 |
| 10-19-2010 | 1 | 7 | 2.3 | -2.7 | 69 | 1001.2 |
| 10-20-2010 | 1 | 9 | 0.8 | -4.0 | 69 | 1004.7 |
| 10-21-2010 | 3 | 8 | 0.5 | -4.8 | 68 | 1006.9 |
| 10-22-2010 | 2 | 7 | 2.3 | -3.1 | 67 | 1013.8 |
| 10-23-2010 | 1 | 11 | 0.4 | -3.8 | 74 | 1015.1 |
| 10-24-2010 | 1 | 10 | 0.6 | -2.7 | 79 | 1013.0 |
| 10-25-2010 | 0 | 12 | 0.1 | -3.5 | 79 | 1011.3 |
| 10-26-2010 | 1 | 8 | 0.9 | -3.3 | 75 | 1010.4 |
| 10-27-2010 | 0 | 14 | 1.3 | -3.0 | 75 | 1004.4 |
| 10-28-2010 | 3 | 13 | 2.7 | -4.0 | 65 | 1001.3 |
| 10-29-2010 | 0 | 15 | 2.0 | -4.2 | 65 | 1001.5 |
| 10-30-2010 | 0 | 10 | 2.5 | -2.7 | 68 | 1004.8 |
| 10-31-2010 | 0 | 0 | 7.5 | -1.8 | 52 | 1002.8 |
| 11-01-2010 | 0 | 9 | 6.1 | 0.7 | 71 | 1001.1 |
| 11-02-2010 | 2 | 13 | 8.8 | -1.3 | 51 | 998.2 |
| 11-03-2010 | 1 | 6 | 4.8 | 0.9 | 77 | 995.1 |
| 11-04-2010 | 0 | 4 | 1.8 | -0.1 | 87 | 1004.3 |
| 11-05-2010 | 0 | 4 | 3.0 | -0.8 | 76 | 1005.5 |
| 11-06-2010 | 0 | 14 | 3.1 | -0.8 | 76 | 999.6 |
| 11-07-2010 | 2 | 6 | 5.5 | 0.4 | 72 | 990.0 |
| 11-08-2010 | 2 | 8 | 3.2 | 0.4 | 82 | 993.2 |
| 11-09-2010 | 2 | 10 | 0.4 | -2.9 | 78 | 1008.7 |
| 11-10-2010 | 0 | 10 | 1.2 | -2.4 | 78 | 1005.1 |
| 11-11-2010 | 1 | 4 | -3.5 | -7.3 | 73 | 1008.6 |
| 11-12-2010 | 0 | 12 | -3.8 | -6.2 | 81 | 1003.8 |
| 11-13-2010 | 1 | 9 | 2.7 | -2.7 | 68 | 997.1 |
| 11-14-2010 | 2 | 3 | 6.9 | -1.1 | 58 | 995.8 |
| 11-15-2010 | 1 | 10 | 5.5 | 0.8 | 74 | 983.8 |
| 11-16-2010 | 0 | 10 | 2.5 | 0.5 | 86 | 986.8 |
| 11-17-2010 | 0 | 11 | 1.2 | -0.5 | 88 | 988.1 |
| 11-18-2010 | 1 | 7 | -2.1 | -4.9 | 79 | 991.7 |
| 11-19-2010 | 0 | 14 | -5.4 | -7.9 | 80 | 999.5 |
| 11-20-2010 | 0 | 3 | -2.6 | -6.3 | 74 | 993.6 |
| 11-21-2010 | 0 | 4 | -6.2 | -10.6 | 68 | 991.0 |
| 11-22-2010 | 0 | 16 | -15.6 | -19.1 | 72 | 1007.0 |
| 11-23-2010 | 0 | 7 | -13.6 | -16.8 | 74 | 1000.5 |
| 11-24-2010 | 0 | 9 | -8.6 | -11.1 | 80 | 995.2 |
| 11-25-2010 | 1 | 12 | -9.9 | -13.8 | 70 | 1003.8 |
| 11-26-2010 | 1 | 2 | -4.8 | -5.9 | 91 | 1000.7 |
| 11-27-2010 | 2 | 7 | -0.7 | -2.3 | 88 | 994.1 |
| 11-28-2010 | 0 | 9 | -3.8 | -7.2 | 75 | 994.0 |
| 11-29-2010 | 0 | 17 | 0.1 | -1.3 | 90 | 980.2 |
| 11-30-2010 | 0 | 12 | -13.2 | -16.4 | 74 | 993.2 |
| 12-01-2010 | 0 | 13 | -12.0 | -15.5 | 73 | 994.0 |
| 12-02-2010 | 0 | 5 | -5.7 | -8.1 | 81 | 974.4 |
| 12-03-2010 | 2 | 7 | -20.3 | -23.4 | 74 | 995.9 |
| 12-04-2010 | 0 | 1 | -15.9 | -19.7 | 70 | 1014.3 |
| 12-05-2010 | 0 | 7 | -11.0 | -14.9 | 70 | 1019.0 |
| 12-06-2010 | 0 | 12 | -5.8 | -11.1 | 63 | 1006.4 |
| 12-07-2010 | 0 | 5 | -3.5 | -12.3 | 46 | 995.5 |
| 12-08-2010 | 1 | 14 | -9.1 | -12.1 | 76 | 995.3 |
| 12-09-2010 | 0 | 7 | -23.2 | -25.5 | 79 | 1010.5 |
| 12-10-2010 | 0 | 12 | -15.9 | -17.5 | 86 | 999.0 |
| 12-11-2010 | 3 | 4 | -24.0 | -26.1 | 81 | 1012.6 |
| 12-12-2010 | 0 | 12 | -29.1 | -31.5 | 77 | 1017.3 |
| 12-13-2010 | 0 | 7 | -23.2 | -25.8 | 76 | 1020.6 |
| 12-14-2010 | 0 | 7 | -21.1 | -24.0 | 75 | 1016.9 |
| 12-15-2010 | 2 | 7 | -17.5 | -20.7 | 74 | 1005.6 |
| 12-16-2010 | 1 | 11 | -12.8 | -17.4 | 66 | 993.0 |
| 12-17-2010 | 0 | 21 | -6.2 | -8.5 | 82 | 992.7 |
| 12-18-2010 | 0 | 11 | -7.2 | -8.7 | 87 | 991.4 |
| 12-19-2010 | 0 | 12 | -24.8 | -26.8 | 81 | 1002.2 |
| 12-20-2010 | 1 | 13 | -32.0 | -35.0 | 72 | 1010.2 |
| 12-21-2010 | 0 | 12 | -31.4 | -33.9 | 76 | 1013.5 |
| 12-22-2010 | 0 | 2 | -35.3 | -38.3 | 71 | 1017.2 |
| 12-23-2010 | 1 | 5 | -25.7 | -29.0 | 72 | 1010.1 |
| 12-24-2010 | 3 | 14 | -8.1 | -10.2 | 82 | 992.3 |
| 12-25-2010 | 1 | 11 | -21.8 | -24.4 | 77 | 1002.4 |
| 12-26-2010 | 1 | 6 | -21.1 | -23.6 | 78 | 1002.9 |
| 12-27-2010 | 0 | 15 | -21.4 | -24.2 | 76 | 1005.9 |
| 12-28-2010 | 0 | 6 | -24.9 | -27.9 | 73 | 1021.5 |
| 12-29-2010 | 0 | 9 | -19.8 | -22.4 | 77 | 1009.6 |
| 12-30-2010 | 0 | 7 | -16.6 | -19.3 | 77 | 1001.2 |
| 12-31-2010 | 1 | 7 | -24.3 | -27.0 | 76 | 1014.8 |
| 01-01-2011 | 1 | 15 | -28.0 | -30.4 | 77 | 1024.1 |
| 01-02-2011 | 1 | 7 | -32.9 | -35.2 | 78 | 1027.0 |
| 01-03-2011 | 1 | 10 | -35.5 | -38.4 | 72 | 1028.2 |
| 01-04-2011 | 0 | 12 | -33.9 | -36.8 | 72 | 1028.6 |
| 01-05-2011 | 1 | 12 | -34.9 | -38.0 | 70 | 1028.4 |
| 01-06-2011 | 1 | 11 | -34.9 | -37.5 | 75 | 1027.5 |
| 01-07-2011 | 0 | 5 | -33.9 | -37.0 | 71 | 1029.4 |
| 01-08-2011 | 0 | 11 | -31.7 | -35.0 | 70 | 1026.7 |
| 01-09-2011 | 1 | 15 | -29.7 | -33.0 | 71 | 1020.2 |
| 01-10-2011 | 2 | 12 | -25.9 | -29.1 | 72 | 1009.5 |
| 01-11-2011 | 0 | 13 | -20.1 | -22.9 | 76 | 999.7 |
| 01-12-2011 | 0 | 8 | -22.7 | -24.8 | 81 | 1009.8 |
| 01-13-2011 | 1 | 4 | -13.1 | -15.5 | 80 | 1011.0 |
| 01-14-2011 | 1 | 8 | -11.3 | -15.3 | 69 | 1014.6 |
| 01-15-2011 | 1 | 13 | -15.4 | -18.4 | 76 | 1015.0 |
| 01-16-2011 | 0 | 4 | -19.2 | -21.0 | 84 | 1011.8 |
| 01-17-2011 | 2 | 10 | -21.6 | -23.6 | 82 | 1006.8 |
| 01-18-2011 | 0 | 14 | -25.1 | -27.6 | 77 | 1013.5 |
| 01-19-2011 | 1 | 8 | -28.0 | -30.2 | 79 | 1017.4 |
| 01-20-2011 | 0 | 10 | -26.4 | -28.7 | 78 | 1017.7 |
| 01-21-2011 | 1 | 7 | -26.0 | -28.6 | 77 | 1017.7 |
| 01-22-2011 | 1 | 12 | -27.0 | -29.6 | 76 | 1016.3 |
| 01-23-2011 | 1 | 5 | -27.4 | -30.0 | 76 | 1014.0 |
| 01-24-2011 | 3 | 7 | -27.1 | -29.8 | 75 | 1013.8 |
| 01-25-2011 | 0 | 9 | -26.0 | -28.6 | 76 | 1022.3 |
| 01-26-2011 | 1 | 8 | -22.8 | -26.0 | 73 | 1024.5 |
| 01-27-2011 | 1 | 8 | -14.7 | -18.9 | 68 | 1018.4 |
| 01-28-2011 | 2 | 12 | -15.4 | -20.4 | 63 | 1008.8 |
| 01-29-2011 | 0 | 10 | -12.8 | -18.8 | 58 | 998.7 |
| 01-30-2011 | 0 | 12 | -5.1 | -9.9 | 66 | 992.6 |
| 01-31-2011 | 0 | 14 | -10.4 | -14.0 | 72 | 1000.3 |
| 02-01-2011 | 1 | 18 | -10.1 | -13.7 | 73 | 997.0 |
| 02-02-2011 | 0 | 14 | -10.4 | -12.6 | 82 | 997.7 |
| 02-03-2011 | 0 | 14 | -5.6 | -6.9 | 89 | 983.5 |
| 02-04-2011 | 1 | 7 | -13.6 | -16.7 | 75 | 997.6 |
| 02-05-2011 | 0 | 7 | -11.9 | -14.1 | 82 | 992.5 |
| 02-06-2011 | 1 | 6 | -11.9 | -13.7 | 85 | 997.8 |
| 02-07-2011 | 1 | 13 | -14.9 | -16.9 | 83 | 1000.3 |
| 02-08-2011 | 0 | 15 | -12.3 | -15.5 | 76 | 995.7 |
| 02-09-2011 | 0 | 7 | -5.4 | -6.3 | 92 | 997.9 |
| 02-10-2011 | 0 | 6 | -11.7 | -13.7 | 84 | 1009.3 |
| 02-11-2011 | 0 | 12 | -10.4 | -13.8 | 75 | 998.3 |
| 02-12-2011 | 1 | 11 | -8.5 | -10.9 | 81 | 997.4 |
| 02-13-2011 | 0 | 3 | -10.1 | -12.0 | 85 | 994.4 |
| 02-14-2011 | 0 | 5 | -18.3 | -19.8 | 86 | 1002.0 |
| 02-15-2011 | 0 | 12 | -13.2 | -16.2 | 76 | 999.5 |
| 02-16-2011 | 2 | 9 | -5.6 | -7.8 | 83 | 997.7 |
| 02-17-2011 | 1 | 11 | -2.0 | -7.2 | 66 | 989.4 |
| 02-18-2011 | 2 | 5 | -11.3 | -17.8 | 54 | 995.9 |
| 02-19-2011 | 0 | 6 | -18.3 | -22.4 | 68 | 1001.1 |
| 02-20-2011 | 0 | 10 | -18.0 | -20.8 | 77 | 1001.7 |
| 02-21-2011 | 0 | 4 | -22.0 | -25.3 | 72 | 1007.7 |
| 02-22-2011 | 1 | 5 | -27.2 | -30.1 | 74 | 1008.6 |
| 02-23-2011 | 0 | 4 | -22.7 | -26.7 | 67 | 1004.7 |
| 02-24-2011 | 0 | 13 | -21.8 | -26.0 | 66 | 1017.1 |
| 02-25-2011 | 1 | 15 | -23.1 | -26.6 | 72 | 1022.9 |
| 02-26-2011 | 0 | 11 | -18.8 | -21.8 | 75 | 1022.1 |
| 02-27-2011 | 2 | 7 | -20.4 | -23.5 | 74 | 1021.4 |
| 02-28-2011 | 1 | 10 | -20.0 | -23.5 | 71 | 1018.3 |
| 03-01-2011 | 2 | 11 | -18.5 | -22.5 | 71 | 1012.7 |
| 03-02-2011 | 1 | 11 | -17.1 | -20.7 | 71 | 1003.7 |
| 03-03-2011 | 0 | 5 | -16.3 | -19.4 | 75 | 1004.6 |
| 03-04-2011 | 1 | 7 | -16.0 | -20.2 | 69 | 1005.6 |
| 03-05-2011 | 0 | 12 | -13.9 | -18.7 | 65 | 1004.6 |
| 03-06-2011 | 1 | 4 | -8.2 | -13.1 | 65 | 1007.3 |
| 03-07-2011 | 0 | 3 | -3.8 | -9.5 | 61 | 1006.2 |
| 03-08-2011 | 1 | 5 | -2.3 | -5.7 | 75 | 1009.3 |
| 03-09-2011 | 1 | 13 | -1.3 | -5.9 | 72 | 996.0 |
| 03-10-2011 | 2 | 11 | -9.8 | -13.3 | 73 | 993.6 |
| 03-11-2011 | 0 | 5 | -12.7 | -15.6 | 77 | 1004.9 |
| 03-12-2011 | 2 | 8 | -14.6 | -19.0 | 67 | 1021.8 |
| 03-13-2011 | 0 | 6 | -10.6 | -16.2 | 63 | 1020.7 |
| 03-14-2011 | 3 | 8 | -5.3 | -11.9 | 57 | 1003.0 |
| 03-15-2011 | 2 | 16 | -0.9 | -5.0 | 72 | 988.8 |
| 03-16-2011 | 1 | 4 | -5.2 | -7.8 | 79 | 1005.9 |
| 03-17-2011 | 1 | 6 | -5.3 | -7.0 | 86 | 1000.6 |
| 03-18-2011 | 1 | 16 | -10.7 | -14.8 | 71 | 1014.1 |
| 03-19-2011 | 3 | 7 | -11.1 | -13.6 | 80 | 1019.2 |
| 03-20-2011 | 1 | 7 | -9.3 | -11.8 | 81 | 1024.1 |
| 03-21-2011 | 3 | 7 | -9.5 | -11.5 | 85 | 1022.5 |
| 03-22-2011 | 2 | 5 | -8.1 | -12.2 | 73 | 1015.4 |
| 03-23-2011 | 0 | 8 | -6.6 | -11.3 | 70 | 1009.0 |
| 03-24-2011 | 0 | 4 | -2.6 | -10.4 | 56 | 1000.7 |
| 03-25-2011 | 0 | 5 | 2.0 | -4.7 | 65 | 992.4 |
| 03-26-2011 | 1 | 6 | 3.0 | -0.6 | 77 | 994.6 |
| 03-27-2011 | 1 | 2 | -0.9 | -8.1 | 55 | 1005.0 |
| 03-28-2011 | 0 | 10 | 0.5 | -3.6 | 74 | 999.1 |
| 03-29-2011 | 1 | 8 | -6.2 | -10.3 | 71 | 1012.0 |
| 03-30-2011 | 2 | 9 | -4.9 | -12.3 | 55 | 1010.3 |
| 03-31-2011 | 1 | 14 | -3.1 | -8.6 | 63 | 1003.2 |
| 04-01-2011 | 0 | 5 | 3.3 | -3.2 | 62 | 997.5 |
| 04-02-2011 | 1 | 1 | 2.7 | -0.6 | 80 | 991.5 |
| 04-03-2011 | 1 | 10 | -1.8 | -3.5 | 86 | 994.7 |
| 04-04-2011 | 4 | 8 | -4.1 | -7.2 | 77 | 1005.1 |
| 04-05-2011 | 0 | 3 | 0.7 | -1.0 | 87 | 998.7 |
| 04-06-2011 | 1 | 2 | 2.5 | 0.1 | 83 | 1003.5 |
| 04-07-2011 | 3 | 3 | 3.2 | -0.1 | 79 | 997.5 |
| 04-08-2011 | 0 | 5 | 3.3 | -0.8 | 76 | 1000.3 |
| 04-09-2011 | 0 | 9 | 5.3 | -0.9 | 66 | 992.7 |
| 04-10-2011 | 1 | 3 | 8.0 | 0.3 | 61 | 988.0 |
| 04-11-2011 | 1 | 6 | 8.9 | 2.9 | 67 | 992.2 |
| 04-12-2011 | 0 | 8 | 12.5 | 2.7 | 56 | 994.6 |
| 04-13-2011 | 0 | 6 | 14.2 | 4.5 | 55 | 996.1 |
| 04-14-2011 | 0 | 5 | 14.1 | 5.2 | 59 | 994.1 |
| 04-15-2011 | 2 | 7 | 13.8 | 5.8 | 62 | 993.1 |
| 04-16-2011 | 0 | 3 | 16.7 | 4.6 | 50 | 991.4 |
| 04-17-2011 | 0 | 10 | 8.8 | 2.8 | 69 | 1000.0 |
| 04-18-2011 | 2 | 10 | 10.9 | -1.7 | 45 | 1001.2 |
| 04-19-2011 | 3 | 10 | 13.7 | 1.5 | 45 | 992.6 |
| 04-20-2011 | 2 | 8 | 10.7 | 3.0 | 61 | 997.7 |
| 04-21-2011 | 0 | 5 | 12.8 | 2.3 | 52 | 999.3 |
| 04-22-2011 | 0 | 8 | 20.7 | 4.1 | 35 | 989.3 |
| 04-23-2011 | 0 | 3 | 9.5 | -1.4 | 47 | 991.5 |
| 04-24-2011 | 0 | 7 | 4.5 | -4.1 | 54 | 998.8 |
| 04-25-2011 | 1 | 12 | 9.1 | -4.4 | 41 | 991.7 |
| 04-26-2011 | 1 | 11 | 8.2 | 3.2 | 72 | 990.3 |
| 04-27-2011 | 1 | 9 | 7.2 | 2.9 | 75 | 986.8 |
| 04-28-2011 | 1 | 21 | 4.2 | -0.8 | 72 | 997.4 |
| 04-29-2011 | 2 | 3 | 7.7 | -0.9 | 58 | 998.7 |
| 04-30-2011 | 0 | 2 | 9.4 | -2.6 | 48 | 996.5 |
| 05-01-2011 | 1 | 10 | 12.5 | 0.3 | 46 | 999.7 |
| 05-02-2011 | 1 | 3 | 8.8 | 0.7 | 62 | 1001.7 |
| 05-03-2011 | 1 | 5 | 6.7 | -3.8 | 51 | 1000.7 |
| 05-04-2011 | 2 | 11 | 8.2 | -4.8 | 42 | 993.7 |
| 05-05-2011 | 2 | 13 | 4.8 | -2.3 | 62 | 991.8 |
| 05-06-2011 | 3 | 8 | 6.4 | 0.8 | 69 | 993.2 |
| 05-07-2011 | 2 | 7 | 11.3 | -2.1 | 41 | 993.4 |
| 05-08-2011 | 0 | 9 | 3.3 | -4.7 | 57 | 995.0 |
| 05-09-2011 | 0 | 6 | 2.3 | -7.4 | 48 | 1002.2 |
| 05-10-2011 | 2 | 10 | 9.5 | -6.2 | 33 | 1002.4 |
| 05-11-2011 | 1 | 16 | 14.1 | -5.5 | 26 | 995.8 |
| 05-12-2011 | 4 | 14 | 18.4 | -5.6 | 20 | 990.7 |
| 05-13-2011 | 1 | 10 | 19.3 | 1.2 | 35 | 986.8 |
| 05-14-2011 | 2 | 6 | 10.2 | -1.4 | 48 | 990.7 |
| 05-15-2011 | 1 | 7 | 13.4 | 5.8 | 61 | 986.2 |
| 05-16-2011 | 4 | 10 | 7.2 | -4.1 | 47 | 999.7 |
| 05-17-2011 | 1 | 11 | 6.3 | -6.1 | 43 | 1004.1 |
| 05-18-2011 | 1 | 13 | 8.9 | -3.7 | 43 | 1003.7 |
| 05-19-2011 | 0 | 6 | 12.1 | -2.5 | 40 | 999.5 |
| 05-20-2011 | 2 | 4 | 18.7 | 1.1 | 34 | 990.7 |
| 05-21-2011 | 0 | 5 | 15.5 | 8.6 | 66 | 986.3 |
| 05-22-2011 | 1 | 5 | 13.9 | 9.2 | 74 | 987.1 |
| 05-23-2011 | 2 | 8 | 12.9 | 5.0 | 62 | 990.2 |
| 05-24-2011 | 0 | 7 | 14.2 | 3.2 | 50 | 990.0 |
| 05-25-2011 | 1 | 1 | 19.1 | 6.8 | 47 | 983.3 |
| 05-26-2011 | 1 | 14 | 18.8 | 8.1 | 51 | 979.4 |
| 05-27-2011 | 2 | 3 | 12.2 | 4.4 | 59 | 988.9 |
| 05-28-2011 | 1 | 6 | 14.5 | 9.2 | 72 | 990.9 |
| 05-29-2011 | 0 | 2 | 17.4 | 11.4 | 70 | 994.7 |
| 05-30-2011 | 2 | 13 | 22.6 | 9.4 | 45 | 990.4 |
| 05-31-2011 | 0 | 2 | 22.2 | 9.8 | 51 | 984.3 |
| 06-01-2011 | 0 | 11 | 16.2 | 11.6 | 75 | 985.8 |
| 06-02-2011 | 2 | 9 | 21.3 | 12.8 | 60 | 987.5 |
| 06-03-2011 | 0 | 10 | 20.9 | 13.8 | 65 | 985.5 |
| 06-04-2011 | 1 | 6 | 19.5 | 14.0 | 71 | 984.0 |
| 06-05-2011 | 2 | 8 | 20.3 | 13.1 | 65 | 985.3 |
| 06-06-2011 | 1 | 11 | 18.2 | 12.8 | 71 | 988.2 |
| 06-07-2011 | 0 | 7 | 20.5 | 12.2 | 62 | 990.6 |
| 06-08-2011 | 3 | 10 | 22.2 | 13.6 | 62 | 989.2 |
| 06-09-2011 | 2 | 7 | 23.1 | 15.9 | 66 | 985.2 |
| 06-10-2011 | 0 | 11 | 22.5 | 15.6 | 67 | 981.0 |
| 06-11-2011 | 0 | 10 | 22.9 | 15.4 | 63 | 979.3 |
| 06-12-2011 | 2 | 2 | 21.1 | 16.1 | 74 | 979.7 |
| 06-13-2011 | 3 | 10 | 22.8 | 14.3 | 61 | 980.3 |
| 06-14-2011 | 2 | 9 | 20.8 | 11.6 | 56 | 985.9 |
| 06-15-2011 | 0 | 13 | 17.4 | 8.3 | 57 | 988.8 |
| 06-16-2011 | 1 | 5 | 17.2 | 5.7 | 51 | 990.8 |
| 06-17-2011 | 3 | 5 | 18.9 | 8.4 | 54 | 989.2 |
| 06-18-2011 | 1 | 10 | 22.5 | 11.5 | 52 | 986.1 |
| 06-19-2011 | 0 | 8 | 23.7 | 13.4 | 55 | 978.3 |
| 06-20-2011 | 1 | 12 | 21.1 | 12.0 | 59 | 976.9 |
| 06-21-2011 | 2 | 18 | 19.0 | 10.5 | 61 | 979.1 |
| 06-22-2011 | 1 | 6 | 15.9 | 12.8 | 81 | 979.5 |
| 06-23-2011 | 1 | 20 | 16.9 | 13.1 | 79 | 982.4 |
| 06-24-2011 | 6 | 22 | 19.3 | 14.8 | 76 | 987.1 |
| 06-25-2011 | 6 | 10 | 18.2 | 10.6 | 64 | 987.8 |
| 06-26-2011 | 2 | 9 | 17.0 | 9.9 | 64 | 987.6 |
| 06-27-2011 | 1 | 8 | 18.6 | 10.2 | 60 | 986.6 |
| 06-28-2011 | 0 | 4 | 20.4 | 10.0 | 54 | 985.8 |
| 06-29-2011 | 0 | 8 | 22.6 | 12.0 | 55 | 986.3 |
| 06-30-2011 | 1 | 8 | 24.3 | 14.2 | 55 | 980.1 |
| 07-01-2011 | 2 | 6 | 21.0 | 16.3 | 76 | 974.0 |
| 07-02-2011 | 1 | 5 | 14.7 | 10.4 | 77 | 981.2 |
| 07-03-2011 | 0 | 1 | 12.3 | 9.7 | 84 | 988.7 |
| 07-04-2011 | 2 | 8 | 13.2 | 6.7 | 68 | 997.3 |
| 07-05-2011 | 3 | 3 | 17.5 | 7.3 | 53 | 994.1 |
| 07-06-2011 | 2 | 14 | 20.4 | 12.0 | 60 | 990.8 |
| 07-07-2011 | 0 | 11 | 15.8 | 6.7 | 56 | 997.3 |
| 07-08-2011 | 0 | 10 | 15.9 | 6.0 | 55 | 997.2 |
| 07-09-2011 | 0 | 2 | 16.9 | 10.1 | 66 | 989.2 |
| 07-10-2011 | 0 | 9 | 17.9 | 9.6 | 60 | 989.5 |
| 07-11-2011 | 2 | 9 | 22.0 | 15.3 | 67 | 979.4 |
| 07-12-2011 | 2 | 9 | 22.9 | 16.9 | 70 | 977.5 |
| 07-13-2011 | 0 | 10 | 21.9 | 12.6 | 57 | 976.0 |
| 07-14-2011 | 0 | 8 | 16.8 | 8.2 | 59 | 987.1 |
| 07-15-2011 | 1 | 11 | 19.1 | 9.5 | 57 | 987.3 |
| 07-16-2011 | 0 | 6 | 16.3 | 12.1 | 77 | 989.3 |
| 07-17-2011 | 0 | 3 | 19.6 | 11.7 | 64 | 988.7 |
| 07-18-2011 | 0 | 14 | 21.2 | 11.6 | 57 | 988.1 |
| 07-19-2011 | 1 | 7 | 21.3 | 11.6 | 58 | 986.8 |
| 07-20-2011 | 1 | 13 | 22.6 | 12.6 | 56 | 982.0 |
| 07-21-2011 | 2 | 8 | 16.7 | 11.7 | 72 | 985.4 |
| 07-22-2011 | 1 | 9 | 16.3 | 7.1 | 57 | 990.6 |
| 07-23-2011 | 0 | 11 | 16.5 | 7.8 | 59 | 988.2 |
| 07-24-2011 | 1 | 6 | 18.7 | 10.5 | 60 | 988.0 |
| 07-25-2011 | 0 | 20 | 19.6 | 14.0 | 73 | 988.3 |
| 07-26-2011 | 0 | 7 | 17.5 | 8.4 | 57 | 996.5 |
| 07-27-2011 | 0 | 9 | 19.1 | 12.9 | 69 | 991.0 |
| 07-28-2011 | 3 | 7 | 16.6 | 9.3 | 65 | 988.4 |
| 07-29-2011 | 2 | 9 | 14.2 | 8.2 | 69 | 989.5 |
| 07-30-2011 | 2 | 13 | 16.4 | 8.0 | 59 | 987.4 |
| 07-31-2011 | 1 | 9 | 21.0 | 12.0 | 58 | 980.2 |
| 08-01-2011 | 3 | 16 | 26.0 | 15.3 | 54 | 977.0 |
| 08-02-2011 | 5 | 17 | 21.6 | 12.8 | 60 | 982.0 |
| 08-03-2011 | 1 | 9 | 20.7 | 16.4 | 78 | 978.5 |
| 08-04-2011 | 1 | 10 | 16.2 | 11.6 | 76 | 981.9 |
| 08-05-2011 | 1 | 13 | 13.8 | 9.3 | 76 | 985.7 |
| 08-06-2011 | 1 | 7 | 12.8 | 8.0 | 75 | 990.2 |
| 08-07-2011 | 1 | 3 | 14.9 | 7.1 | 62 | 984.7 |
| 08-08-2011 | 8 | 8 | 15.1 | 12.6 | 85 | 980.5 |
| 08-09-2011 | 1 | 8 | 14.1 | 11.8 | 86 | 988.6 |
| 08-10-2011 | 0 | 9 | 17.7 | 9.9 | 64 | 994.4 |
| 08-11-2011 | 6 | 7 | 17.7 | 13.4 | 76 | 994.1 |
| 08-12-2011 | 2 | 7 | 18.0 | 12.8 | 74 | 995.0 |
| 08-13-2011 | 1 | 9 | 18.9 | 11.9 | 67 | 996.0 |
| 08-14-2011 | 2 | 11 | 19.7 | 12.1 | 65 | 996.1 |
| 08-15-2011 | 1 | 14 | 20.4 | 12.3 | 64 | 993.9 |
| 08-16-2011 | 1 | 7 | 21.9 | 11.4 | 55 | 985.6 |
| 08-17-2011 | 2 | 16 | 18.0 | 13.5 | 75 | 984.0 |
| 08-18-2011 | 0 | 6 | 13.6 | 9.3 | 78 | 983.6 |
| 08-19-2011 | 0 | 12 | 12.7 | 9.5 | 82 | 984.5 |
| 08-20-2011 | 0 | 10 | 11.2 | 10.1 | 93 | 985.3 |
| 08-21-2011 | 1 | 5 | 13.5 | 7.7 | 71 | 992.8 |
| 08-22-2011 | 4 | 6 | 14.8 | 8.3 | 67 | 991.0 |
| 08-23-2011 | 1 | 6 | 15.9 | 6.9 | 57 | 991.5 |
| 08-24-2011 | 2 | 4 | 20.9 | 11.3 | 57 | 979.1 |
| 08-25-2011 | 3 | 4 | 15.3 | 13.1 | 86 | 981.5 |
| 08-26-2011 | 0 | 3 | 12.4 | 3.9 | 59 | 988.3 |
| 08-27-2011 | 1 | 3 | 10.4 | 5.8 | 75 | 992.1 |
| 08-28-2011 | 2 | 7 | 10.5 | 7.5 | 83 | 993.1 |
| 08-29-2011 | 1 | 8 | 12.7 | 4.4 | 61 | 992.4 |
| 08-30-2011 | 1 | 6 | 16.0 | 6.7 | 57 | 989.7 |
| 08-31-2011 | 6 | 7 | 15.9 | 11.8 | 77 | 993.0 |
| 09-01-2011 | 5 | 6 | 18.4 | 12.4 | 72 | 988.8 |
| 09-02-2011 | 2 | 11 | 8.3 | 4.8 | 80 | 998.0 |
| 09-03-2011 | 1 | 3 | 15.7 | 5.4 | 53 | 997.0 |
| 09-04-2011 | 0 | 7 | 11.8 | 8.6 | 82 | 1000.3 |
| 09-05-2011 | 1 | 8 | 11.1 | 3.6 | 63 | 1004.5 |
| 09-06-2011 | 5 | 12 | 10.8 | 2.2 | 58 | 1001.9 |
| 09-07-2011 | 2 | 11 | 12.1 | 1.4 | 51 | 995.4 |
| 09-08-2011 | 3 | 6 | 13.0 | 4.6 | 61 | 987.8 |
| 09-09-2011 | 0 | 5 | 17.8 | 5.8 | 47 | 981.9 |
| 09-10-2011 | 0 | 2 | 12.6 | 7.8 | 73 | 988.2 |
| 09-11-2011 | 1 | 6 | 9.2 | 3.9 | 72 | 998.8 |
| 09-12-2011 | 0 | 14 | 8.8 | 2.2 | 67 | 1003.1 |
| 09-13-2011 | 3 | 12 | 10.1 | 0.1 | 54 | 1003.3 |
| 09-14-2011 | 2 | 8 | 10.8 | -0.3 | 48 | 1000.4 |
| 09-15-2011 | 1 | 5 | 12.4 | -0.2 | 44 | 996.0 |
| 09-16-2011 | 0 | 11 | 11.5 | 1.1 | 53 | 994.5 |
| 09-17-2011 | 2 | 5 | 11.9 | 0.4 | 51 | 995.8 |
| 09-18-2011 | 1 | 7 | 11.7 | 0.9 | 53 | 996.7 |
| 09-19-2011 | 3 | 6 | 13.4 | 2.2 | 50 | 995.1 |
| 09-20-2011 | 2 | 17 | 17.5 | 5.2 | 46 | 989.7 |
| 09-21-2011 | 2 | 7 | 17.6 | 8.3 | 57 | 990.1 |
| 09-22-2011 | 0 | 9 | 11.9 | 8.8 | 81 | 993.3 |
| 09-23-2011 | 0 | 13 | 12.3 | 5.1 | 62 | 992.8 |
| 09-24-2011 | 0 | 6 | 9.6 | 5.6 | 78 | 988.2 |
| 09-25-2011 | 2 | 4 | 4.3 | 2.6 | 88 | 998.3 |
| 09-26-2011 | 1 | 9 | 5.1 | 1.7 | 81 | 1007.6 |
| 09-27-2011 | 0 | 13 | 8.3 | 1.3 | 66 | 1004.8 |
| 09-28-2011 | 4 | 6 | 9.4 | 1.7 | 62 | 998.9 |
| 09-29-2011 | 4 | 14 | 11.8 | 3.8 | 63 | 997.7 |
| 09-30-2011 | 0 | 18 | 8.9 | 1.4 | 62 | 1005.0 |
| 10-01-2011 | 0 | 7 | 10.2 | 1.5 | 58 | 1001.4 |
| 10-02-2011 | 1 | 6 | 13.6 | 3.0 | 51 | 994.9 |
| 10-03-2011 | 3 | 9 | 14.8 | 5.3 | 55 | 989.3 |
| 10-04-2011 | 1 | 10 | 11.9 | 11.1 | 95 | 987.4 |
| 10-05-2011 | 2 | 7 | 12.6 | 7.6 | 74 | 986.7 |
| 10-06-2011 | 0 | 3 | 8.5 | 4.0 | 74 | 992.2 |
| 10-07-2011 | 1 | 10 | 8.7 | 3.5 | 70 | 996.5 |
| 10-08-2011 | 1 | 3 | 11.7 | 4.9 | 65 | 992.7 |
| 10-09-2011 | 1 | 13 | 5.2 | 4.2 | 93 | 1001.4 |
| 10-10-2011 | 1 | 7 | 3.3 | 2.8 | 96 | 1009.4 |
| 10-11-2011 | 0 | 4 | 8.3 | 3.2 | 72 | 1006.6 |
| 10-12-2011 | 0 | 4 | 11.4 | 5.8 | 69 | 1006.2 |
| 10-13-2011 | 1 | 3 | 9.9 | 5.4 | 76 | 1004.9 |
| 10-14-2011 | 3 | 5 | 9.3 | 3.5 | 72 | 1001.3 |
| 10-15-2011 | 0 | 6 | 8.8 | 3.1 | 71 | 998.5 |
| 10-16-2011 | 1 | 4 | 11.7 | 2.8 | 57 | 995.5 |
| 10-17-2011 | 3 | 13 | 13.8 | 4.1 | 53 | 991.2 |
| 10-18-2011 | 4 | 12 | 4.0 | 0.8 | 80 | 997.0 |
| 10-19-2011 | 2 | 7 | 2.5 | -3.0 | 67 | 1003.3 |
| 10-20-2011 | 0 | 9 | 2.4 | 1.1 | 91 | 1005.2 |
| 10-21-2011 | 1 | 12 | 3.9 | 1.1 | 82 | 1009.2 |
| 10-22-2011 | 1 | 6 | 2.3 | -2.3 | 73 | 1006.4 |
| 10-23-2011 | 0 | 4 | 1.1 | -2.8 | 76 | 1002.4 |
| 10-24-2011 | 3 | 11 | 2.7 | -1.7 | 75 | 1000.0 |
| 10-25-2011 | 0 | 10 | 7.0 | 2.6 | 75 | 996.3 |
| 10-26-2011 | 3 | 13 | 8.2 | 3.9 | 74 | 993.7 |
| 10-27-2011 | 0 | 12 | 4.5 | 2.0 | 83 | 984.4 |
| 10-28-2011 | 2 | 11 | 0.3 | -2.3 | 82 | 992.7 |
| 10-29-2011 | 0 | 3 | -1.4 | -3.0 | 88 | 992.3 |
| 10-30-2011 | 0 | 9 | -5.4 | -7.4 | 84 | 997.2 |
| 10-31-2011 | 2 | 9 | -3.6 | -5.7 | 84 | 997.3 |
| 11-01-2011 | 0 | 6 | -1.6 | -3.6 | 85 | 994.1 |
| 11-02-2011 | 0 | 5 | -4.4 | -7.4 | 77 | 1001.8 |
| 11-03-2011 | 1 | 6 | 1.2 | -4.2 | 66 | 997.6 |
| 11-04-2011 | 0 | 2 | 1.9 | -3.1 | 69 | 996.1 |
| 11-05-2011 | 1 | 3 | 3.2 | -2.4 | 67 | 996.0 |
| 11-06-2011 | 0 | 8 | 2.0 | -3.2 | 69 | 993.2 |
| 11-07-2011 | 0 | 7 | -5.3 | -7.8 | 81 | 998.9 |
| 11-08-2011 | 2 | 6 | -5.5 | -6.9 | 88 | 995.3 |
| 11-09-2011 | 1 | 10 | -12.2 | -17.4 | 62 | 1006.4 |
| 11-10-2011 | 2 | 8 | -11.1 | -14.3 | 74 | 1004.0 |
| 11-11-2011 | 0 | 5 | -13.6 | -15.9 | 81 | 1008.5 |
| 11-12-2011 | 0 | 3 | -6.6 | -10.0 | 74 | 998.1 |
| 11-13-2011 | 0 | 0 | -3.2 | -7.1 | 72 | 989.9 |
| 11-14-2011 | 1 | 8 | -15.1 | -17.6 | 79 | 998.1 |
| 11-15-2011 | 0 | 5 | -12.4 | -14.0 | 86 | 994.3 |
